# Targeted Genome Mining—From Compound Discovery to Biosynthetic Pathway Elucidation

**DOI:** 10.3390/microorganisms8122034

**Published:** 2020-12-19

**Authors:** Nils Gummerlich, Yuriy Rebets, Constanze Paulus, Josef Zapp, Andriy Luzhetskyy

**Affiliations:** 1 Department of Pharmaceutical Biotechnology, Saarland University, Campus C2.3, 66123 Saarbrücken, Germany; nils.gummerlich@gmx.de (N.G.); yurko.rebets@gmail.com (Y.R.); constanzepaulus@gmail.com (C.P.); 2 Department of Pharmaceutical Biology, Saarland University, Campus C2.3, 66123 Saarbrücken, Germany; j.zapp@mx.uni-saarland.de; 3 Actinobacteria Metabolic Engineering Group, Helmholtz-Institute for Pharmaceutical Research Saarland (HIPS), Campus E8.1, 66123 Saarbrücken, Germany

**Keywords:** angucyclinone, heterologous expression, natural products, Actinobacteria, biosynthesis

## Abstract

Natural products are an important source of novel investigational compounds in drug discovery. Especially in the field of antibiotics, Actinobacteria have been proven to be a reliable source for lead structures. The discovery of these natural products with activity- and structure-guided screenings has been impeded by the constant rediscovery of previously identified compounds. Additionally, a large discrepancy between produced natural products and biosynthetic potential in Actinobacteria, including representatives of the order *Pseudonocardiales*, has been revealed using genome sequencing. To turn this genomic potential into novel natural products, we used an approach including the in-silico pre-selection of unique biosynthetic gene clusters followed by their systematic heterologous expression. As a proof of concept, fifteen *Saccharothrix* *espanaensis* genomic library clones covering predicted biosynthetic gene clusters were chosen for expression in two heterologous hosts, *Streptomyces* *lividans* and *Streptomyces* *albus*. As a result, two novel natural products, an unusual angucyclinone pentangumycin and a new type II polyketide synthase shunt product SEK90, were identified. After purification and structure elucidation, the biosynthetic pathways leading to the formation of pentangumycin and SEK90 were deduced using mutational analysis of the biosynthetic gene cluster and feeding experiments with ^13^C-labelled precursors.

## 1. Introduction

Actinobacteria, especially *Streptomyces*, represent one of the most important sources of natural products [1]. During recent decades, their discovery in Actinobacteria was significantly slowed due to re-isolation of known compounds in activity- and structure-guided screenings [2,3]. As an early solution for the rediscovery issue, natural product databases have been created. Metabolic profiles recorded with high performance liquid chromatography coupled with high-resolution mass spectrometry (LC-HRMS) can be compared with information stored in these databases. This process, known as dereplication, is problematic due to it being time consuming and error prone [4]. An additional problem of this “compound first” approach is the need for laborious studies in order to identify the biosynthetic gene clusters (BGCs) responsible for the production of the isolated compounds. This information is essential to elucidate the biosynthetic pathway, which helps researchers to increase and modify the production of identified natural products [5]. In contrast, the “cluster to compound” approach aims to obtain new natural products after cloning and expression of a BGC of interest in heterologous hosts. This approach became increasingly reasonable due to the low cost of next-generation sequencing [6], which has resulted in a flood of genomic information [7]. It has been shown that *Streptomyces* contain an average of 21.9 BGCs per genome, with 40–48% of these BGCs being unique [8,9]. The “cluster to compound” approach aims to tap into this genomic potential and offers decisive advantages over the “compound first” approach. First, genome analysis and BGC prediction tools such as antiSMASH [10] offer automated cluster homology comparisons within hours. In turn, this allows cataloguing and prioritization of BGCs with unique features. Second, the utilization of optimized heterologous hosts and the expression of BGCs from genomic libraries offer advantages over the use of native producing strains [11,12,13,14]. Furthermore, the produced compounds can be identified by a simple comparison of the metabolic profiles from the heterologous host with and without the expressed BGCs [11].

**Figure 1:**
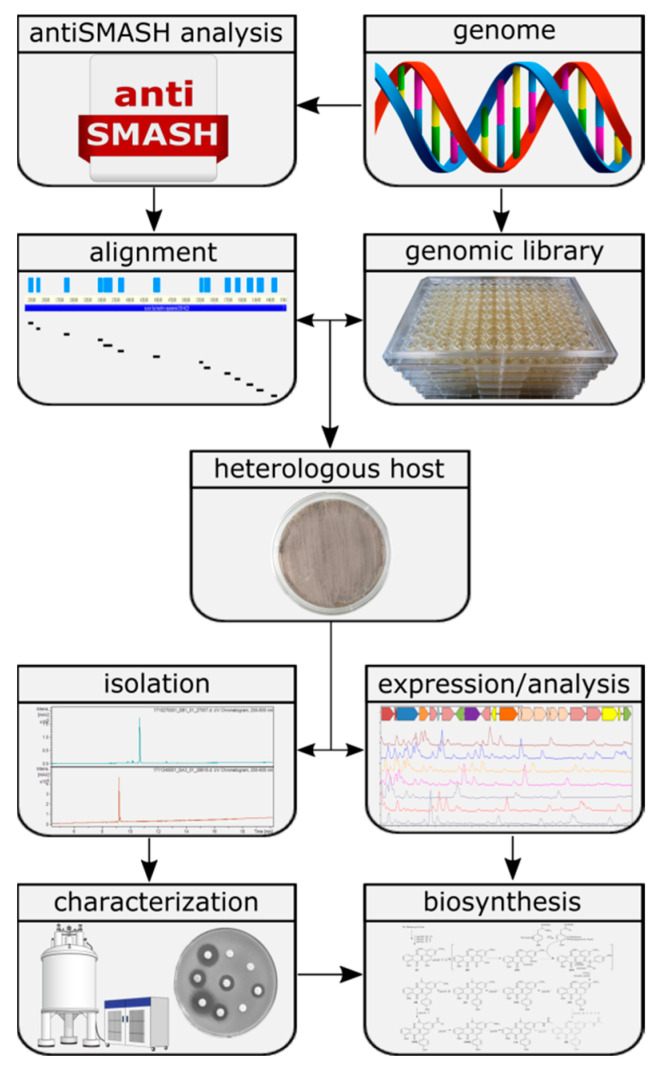
Workflow for the identification of novel natural products using genome mining.

The aim of the presented study was to assess the untapped potential for the production of secondary metabolites hidden in the genomes of Actinobacteria and to identify their biosynthetic pathways [8]. To achieve this goal, a widely applicable workflow was developed (Figure 1). This workflow includes the analysis of genome sequences utilizing algorithms such as antiSMASH [15] and PRISM [16], the construction of a sequenced genomic library, in silico pre-selection and expression of the selected BGCs in optimized heterologous hosts followed by the identification, isolation and characterization of natural products and their biosynthetic pathways. To challenge the applicability of the designed approach, we aimed to express BGCs from a strain of Actinobacteria that is more distantly related to commonly used heterologous *Streptomyces* hosts. The large-scale analysis by Doroghazi and co-authors revealed that the order of *Pseudonocardiales* has an average of 19.8 BGCs per genome [8]. The strain *Saccharothrix espanaensis*, discovered by Labeda and Lechevalier in 1989 [17], belongs to the order of *Pseudonocardiales*, harboring all of the desired elements to prove the potential of our approach. This strain has a 9.3 Mbp genome containing at least 31 antiSMASH-predicted BGCs [18]. Due to the strain being poorly genetically tractable, any genetic manipulations are almost impossible. Furthermore, only a single group of polysaccharide natural products, saccharomicins [19], is known to be produced by *S. espanaensis*.

As proof of concept, we report the discovery of two novel natural products which were obtained by applying the proposed genome mining approach. Neither of the isolated compounds were detectable in the metabolic profile of *S. espanaensis*. Additionally, we were able to elucidate the biosynthesis of both compounds using the available information about the BGC architecture, mutational analysis and feeding experiments with labelled precursors.

## 2. Materials and Methods

### 2.1. General Experimental Procedures

All oligonucleotides, plasmids and strains used in this work can be found in Tables S1 and S2. Standard procedures for *Escherichia coli* (transformation and plasmid preparations) were performed as described by Sambrook [20]. All BACs of the genomic library of *S. espanaensis* were isolated with the BACMAX^TM^ DNA purification kit (Lucigen, Middleton, WI, USA). All *E. coli* cultures were cultivated at 37 °C at 180 rpm in LB-Lennox (5 g yeast extract, 10 g tryptone, 5 g NaCl and 1000 mL H_2_O_dest_; pH = 7.2). For DNA fragment amplification, Dream Taq polymerase (Thermo Fisher Scientific, Waltham, MA, USA) was used. DNA fragments were purified from agarose gels using the QIAquick Gel Extraction Kit (Qiagen, Germany). Intergeneric conjugation between *Streptomyces* and *E. coli* was performed as described by Kieser [21] on MS agar (20 g soya flour, 20 g agar, 20 g mannitol and 1000 mL H_2_O with nalidixic acid 30 μg/mL for donor suppression) plates using *E. coli* PUB307 as donor strain. Standard DNA manipulations (ligation, polymerase chain reactions and endonuclease digestion) were performed according to the corresponding manufacturer’s protocol. All *Streptomyces* were cultivated in 15 mL TSB (17 g Tryptone, 3 g Peptone, 5 g NaCl, 2.5 g K_2_HPO_4_, 2.5 g glucose and 1000 mL H_2_O_dest_; pH = 7.2) in a 100-mL flask (4 baffles; 5 g glass beads), inoculated from spores of the corresponding *Streptomyces* from an MS plate. After 24 h for *S. albus* and 72 h for *S. lividans*, 50 mL of the corresponding production medium (SG medium (20 g glucose, 5 g yeast extract, 10 g bactosoytone, 2 g CaCO_3_ and 1000 mL H_2_O_dest_; pH = 7.2); DNPM medium (dextrin 40 g, soytone 7.5 g, fresh yeasts 5 g, MOPS 21 g and 1000 mL H_2_O_dest_; pH = 6.8); TSB medium; SGG medium (10 g starch, 10 g glycerol, 2.5 g corn steep solids, 5 g Peptone, 2 g yeast extract, 1 g NaCl, 3 g CaCO_3_ and 1000 mL H_2_O_dest_, pH = 7.2)) was inoculated with 1 mL of the seed culture in a 500-mL flask (3 baffles, 10 g glass beads) and incubated for 72–164 h at 180 rpm and 29 °C. Production optimization steps were carried out by adjustment of different parameters (growth time, pH, medium, extracting solvent, addition of XAD -16 Amberlite (2 g/100 mL) after 72 h). The optimization steps were monitored using HPLC-MS. When needed, media were supplemented with the following antibiotics: apramycin 50 μg/mL, hygromycin 50 μg/mL, kanamycin 100 μg/mL and nalidixic acid 30 μg/mL.

All HPLC-MS spectra were recorded on a Dionex Ultimate 3000 (Thermo Fisher Scientific, Waltham, MA, USA) coupled with an AmaZon ETD SL speed, Apollo II ESI (Bruker, Billerica, MA, USA) on a Waters BEH C18 column (100 × 2.1 mm, 1.7 µm) with an 18-min linear gradient from 5% acetonitrile (0.1% formic acid) to 95% acetonitrile (0.1% formic acid). The mass spectra were recorded in centroid mode (200 to 2000 *m*/*z*) at a scan rate of 2 Hz. Prior to analysis, all samples were dissolved in methanol and centrifuged for 10 min at 4 °C and 15,000 rpm. The data were analyzed using Bruker Compass Data Analysis 4.2. High-resolution masses were recorded on a Dionex Ultimate 3000 RSLC HPLC (Thermo Fisher Scientific) coupled with an LTQ Orbitrap (Thermo Fisher Scientific) on a BEH C18 100 × 2.1 mm, 1.7-µm column (Waters) with an 18-min linear gradient from 5% acetonitrile (0.1% formic acid) to 95% acetonitrile (0.1% formic acid). Data analysis was carried out using Xcalibur 3.0. Preparative HPLC was carried out using a Waters Autopurification System equipped with a Waters 2545 Binary Gradient module, a Waters System Fluidics Organizer (SFO), a Waters 2998 Photodiode Array Detector (PAD), a Waters 2767 Sample Manager and a Waters SQ-Detector-2 on Nucleodur C18 Htec 250/21-C18 5 μm column (Macherey-Nagel, Düren, Germany). Data analysis was carried out using MassLynx. Structural elucidation was carried out using nuclear magnetic resonance (NMR) technology. The purified compounds were solved in 300 μL deuterated solvent (DMSO-d_6_, MeOD-d_4_, CDCl_3_) and measured in a corresponding 5-mm Shigemi tube (DEUTERO GMBH; Kastellaun, Germany). NMR data (^1^H, HH-COSY; TOCSY; HMBC, HSQC, ^13^C) were acquired either on a Bruker Ascend 700 spectrometer equipped with a 5-mm TXA Cryoprobe or a Bruker Avance 500 spectrometer equipped with a 5-mm BBO Probe at 300 K (Bruker, BioSpin, GmbH, Rheinstetten, Germany). The data were analyzed using Bruker’s TopSpin 3.5a software.

### 2.2. In-Silico Analysis of Gene Clusters

The genome of *Saccharothrix espanaensis* was analyzed with antiSMASH [15] and the Geneious software. Identified BGCs were mapped to the BAC library. A detailed analysis of 1E5′s BGC was performed with BLAST [22].

### 2.3. Extraction, Dereplication and Isolation

The biomass was separated from the production medium (SG medium (20 g glucose, 5 g yeast extract, 10 g bactosoytone, 2 g CaCO_3_ and 1000 mL H_2_O_dest_; pH = 7.2), 172 h, 29 °C, 180 rpm, pH = 7.2, 1% XAD-16 after 24 h) by centrifugation. Then, 20 mL of the supernatant was extracted with 20 mL ethyl acetate followed by 20 mL butanol for 20 min using a Laboshake LS500 (C. Gerhardt GmbH, Königswinter, Germany) at 160 rpm. The biomass was extracted with a 1:1 mixture of acetone and methanol for 60 min. The solvents were evaporated until dry with a rotary evaporator (150 rpm, 60 °C, 240 mbar for ethyl acetate or 25 mbar for butanol) or under a nitrogen stream at 40 °C. The extracts were analyzed using LC-MS and LC-HRMS. Singular peaks were compared to the databases Dictionary of Natural Products, version 27.1 (CRC Press, Cleveland, OH, USA), Sci-Finder (CAS, Columbus, OH, USA) and Chemspider (Royal Society of Chemistry, Raleigh, NC, USA). For production, 15 L of SG medium inoculated with the corresponding recombinant strain was cultivated. The extracts were evaporated to dryness and dissolved in 10 mL methanol. To purify single compounds, size exclusion chromatography (SEC) was performed. The column was packed with 600 mL Sephadex LH-20 resin (GE Healthcare Europe GmbH, 79,111 Freiburg, Germany) solved in methanol. At a flow rate of 10 mL per 15 min, a fraction collector was used to separate the extract within 24 h. To identify fractions containing the novel substances, every 4th fraction was analyzed by HPLC-MS. Fractions with the corresponding mass were combined and evaporated. Prior to NMR analysis, a preparative HPLC was performed. Preparative HPLC information can be found in Tables S3 and S4. NMR data of pentangumycin (**1**) and SEK90 (**2**) can be found in Table 1.

### 2.4. Modification of BACs

All constructed single knockout BACs were obtained by homologous recombination, as previously described by Gust [14] and Myronovskyi [23], and confirmed by PCR. The recombinant BACs were transformed into *E. coli* PUB307 and conjugated into *S. lividans* ΔYA6. All recombinant strains were cultivated, and their metabolome was analyzed as described above.

### 2.5. Construction of Plasmids

For the construction of the plasmids pTOS_A3_R1 and pTOS_A3_R2, we amplified penR1 and penR2 using PCR and used the ordered oligonucleotides to introduce the sequence of the A3 promotor [24] and a KPNI restriction site in the 3′ oligonucleotide and a HindIII restriction site in the 5′ oligonucleotide. The amplified PCR product and the integrative pTOS plasmid were cut using HindIII and KPNI and ligated by the T4 ligase. The obtained DNA fragment was transformed into *E. coli* PUB307 and conjugated into *S. lividans* ΔYA6. The backbone of pTOS was removed from the genome by expression of pUWL_DRE containing the Dre recombinase.

#### Synthesis of 1E5_CMP1 and 1E5_CMP2

For the synthesis of 1E5_CMP1 and 1E5_CMP2, 6-benzyl-4-Hydroxy-2-pyrone (Wuxi AppTec, Saint Paul, MN, USA) was used under the conditions described by de March. The pyrone was mixed (2:1) with either butyraldehyde or formaldehyde in 3 mL ethanol containing 10 µL piperidine and 10 µL glacial acetic acid. The reaction was performed at room temperature for 24 h. All compounds were purified using the Waters Autopurification System and confirmed by NMR.

Feeding experiments. To prove the incorporation from L-Phenylalanine, L-Tyrosine and L-Tryptophan, a culture of either *S. lividans* ΔYA6_1E5 or *S. lividans* ΔYA6_1E5_A3_R1 was prepared as described above. Thereafter, 10 mg of the labelled amino acid (either L-Phenylalanin, L-Tyrosine or L-Tryptophan) was supplemented in 5 steps: 2 mg after 24, 48, 72, 96 and 120 h. After 164 h of incubation, the supernatant of the production cultures was extracted and the incorporation was quantified with LC-MS.

## 3. Results and Discussion

### 3.1. Selection of Putative Biosynthetic Gene Clusters

Initially, the biosynthetic potential of *S. espanaensis* (txid1179773) was assessed. An antiSMASH analysis of its genome predicted the presence of 31 BGCs, while the only known products are saccharomicins (Table S5) [19]. The BGC of saccharomicins was identified as cluster number 28 [18]. It was originally cloned as a cosmid clone by Berner et al., while attempts at its complete expression in heterologous hosts had failed. Nevertheless, its aglycon was successfully expressed and its biosynthesis was elucidated [25,26]. A bacterial artificial chromosome (BAC) library of *S. espanaensis* was constructed using the pSMART-BAC-S vector as a backbone with an average inserted fragments size of 100 kb. The library was end-sequenced, and the sequences were mapped to the genome of *S. espanaensis*. This allowed the alignment of predicted BGCs to the clones of the genomic library. To minimize the laboratory effort and reduce the putative rediscovery rate of natural products in the described approach, BGCs were prioritized by their class and predicted products. Eight BGCs were not fully covered by the genomic library and, therefore, could not be included into the laboratory approach. The remaining 23 BGCs of the BAC library were composed of three BGCs that were predicted to produce geosmin, melanin and bacteriocin and were, therefore, excluded. Three BGCs shared more than 75% similarity to clusters of assigned products and were, therefore, not used for heterologous expression. Finally, a predicted terpene cluster lacking its synthase and a lantipeptide BGC were excluded as well. Thereafter, 15 remaining BACs covering 17 BGCs were selected for heterologous expression in *S. lividans* ΔYA6 and *S. albus* J1074 (Table S5). Among the 17 BGCs, two were terpene clusters, five were either type I or type II polyketide synthase (PKS) clusters, five were non-ribosomal peptide synthetase (NRPS) clusters, two were type I PKS/NRPS hybrid clusters, one was a lantipeptide cluster, one was an aminoglycoside cluster and one was the polysaccharide cluster encoding saccharomicins BGC.

### 3.2. Success Rate of the Expression System

The chosen BACs were conjugated into *S. lividans* ΔYA6 and *S. albus* J1074. Every further-used transconjugant was tested with colony polymerase chain reaction (PCR) for the presence of the conjugated BAC. Verified transconjugants were cultivated in different production media and metabolites were extracted using different solvents. Their metabolic profiles were analyzed with LC-MS and compared to the metabolic profiles of the empty heterologous hosts. Singular peaks were identified and assumed to be the products, shunt products or intermediates of the expressed BGC. Extracts containing singular peaks were further analyzed by LC-HRMS, and the obtained exact masses were compared to masses in common natural product databases (Dictionary of Natural Products (http://dnp.chemnetbase.com CRC Press, Cleveland, OH, USA), Supernatural [http://bioinf-applied.charite.de/supernatural_new/index.php Charite University of Medicine, Berlin, Germany], StreptomeDB [http://132.230.56.4/streptomedb/ University of Freiburg, Freiburg, Germany) [27,28].

The recombinant strain *S. lividans* ΔYA6_3C18, containing a 115-kb insert of the *S. espanaensis* genome*,* revealed a singular peak (Figure S1) with a mass of 308.1987 Da (*m*/*z* 309.2060 [M+H]^+^, R_t_ = 11.5 min). BAC clone 3C18 carries a type I PKS cluster similar to lavendiols BGC [29] and an unknown NRPS BGC. The low production rate of the produced compound rendered isolation, structure- and biosynthetic elucidation unfeasible. The expression of 3C18 was unsuccessful in *S. albus* J1074.

**Figure 2:**
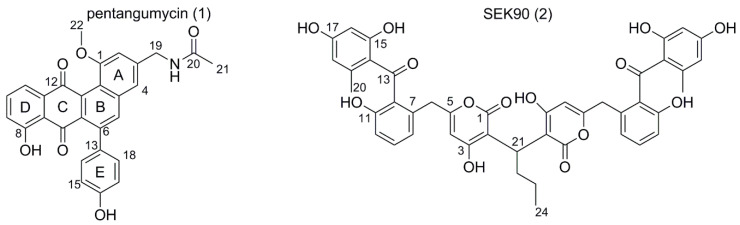
Structural formulas of pentangumycin (**1**) and SEK90 (**2**).

The recombinant strain *S. lividans* ΔYA6_1E5, containing a 116-kb insert from the genome of *S. espanaensis*, produced two singular peaks with masses of 467.1368 (*m*/*z* 468.1438 [M+H]^+^, R_t_ = 9.1 min, further designated as pentangumycin (**1**)) and 790.2261 Da (*m*/*z* 791.2339 [M+H]^+^, R_t_ = 10.6 min; further designated as SEK90 (**2**)), as shown in Figure 2. *S. albus* J1074_1E5 produced compound **1** (Figure S2), but its low production titer rendered further work infeasible. Both compounds **1** and **2** have not been observed in extracts obtained from *S. espanaensis* (Figure S3).

In the case of the remaining 13 BAC clones, no production was observed under the tested conditions described in Section 2.1. An overall success rate of 11% (corresponding to 2 out of 17 BGCs, BAC_3C18 and BAC_1E5) was observed when expressing BGCs from *Pseudonocardiales* in *Streptomyces* hosts. In similar experiments with BGCs from a *Streptomyces* strain, a success rate of 35% (6 out of 17 BGCs) was observed (unpublished data). The generally low expression rate can be explained by a variety of factors: Firstly, differences in the regulatory network of strains can cause major shifts of natural product production. Prominent examples include the mutations in the genes *rpoB rpoB* (C1298T, S443L) and *rpsL* (A262G, K88E) in the strains *S. coelicolor* and *S. lividans*, which activated and increased the production of natural products in both strains [12]. Secondly, codon bias of different strains can be restrictive for the expression of BGCs in heterologous hosts. In fact, it has been shown to be one of the most important factors for the expression of prokaryotic genes [30,31]. In *Escherichia coli*, the heterologous expression of genes was increased by expanding the intracellular concentration of rare tRNA [32]. In *S. lividans*, the expression of a transglutaminase was increased by 73.6%, when using a codon optimized gene for expression [33]. Thirdly, promotors and ribosomal binding sites not derived from the native strain can be unfitting for the polymerase of the host strain and, therefore, disrupt the expression of BGCs. The utilization of strong promotors can activate the expression of BGCs in native and host strains. Salas et al. successfully activated the expression of an NRPS and PKS–NRPS hybrid BGC by introducing the strong and constitutive *erm*E*p promotor in front of the NRPS (*sshg_00313*) and PKS–NRPS (*sshg_05713)* genes. The activation of the NRPS BGC was confirmed by the production of a blue pigment. Furthermore, they identified the natural products 6-epi-alteramid A and B, which were produced by the PKS–NRPS BGC [34]. Fourthly, the toxicity of either expressed proteins or produced natural products is an additional issue to keep in mind when expressing BGCs in heterologous hosts. For example, a mutated version of an outer membrane protein (OmpA) of *E. coli* K12 caused a toxic lysis of the cell when expressed [35]. Furthermore, the toxicity of avermectin was shown to be a restrictive factor in its own production in *S. avermitilis*. After the introduction of multiple copies of the ABC transporter AvtAB, the avermectin production increased from 3.3 to 4.8 g/L [36]. Finally, environmental factors can drastically influence the production of natural products. A well-studied example is the production of jadomycin, since it was only produced after the strain was either treated with a heat shock or the production medium was supplemented with ethanol [37]. Furthermore, it has been proven that the addition of sugars [38] and sub-toxic concentrations of antibiotics can activate and strongly alter the expression of BGCs in bacteria [39,40].

### 3.3. Isolation of Pentangumycin and SEK90

Ultimately, 3.5 mg of pentangumycin **1** and 14 mg of SEK90 **2** were isolated from 15 L of a *S. lividans* ΔYA6_1E5 production culture. Both compounds were purified using size exclusion chromatography and preparative HPLC. Through analysis of LC-HRMS data and NMR data (^1^H-NMR, HSQC, HMBC, ^1^H-^1^H-COSY, ^13^C-NMR), the structures of both compounds were elucidated (Figure 2). NMR data and the detailed data analysis are presented in Table 1 and Figures S4–S50. All chemical shifts observed for **1** were within expected ranges as calculated for its structure. The connection between rings E and B was observed through HMBC correlations between H-5 and C-13 and between H-14/H-18 and C-6 (Figure 2, Figure S4). Compound **1** is a member of the angucyclinone group containing a unique fifth phenol ring attached through a direct carbon–carbon bond at position C-6. Additional ring systems, typically derived from amino acids, have previously been observed in the angucycline family of aromatic polyketides. While such structures have been found in several jadomycins and urdamycins, their position and type of connection are different [41,42,43]. Additionally, formicamycins and fasamycins, which are examples of anthracycline antibiotics, carry a similar structural motif with the fifth aromatic ring at position C-7 of ring B [44]. Furthermore, **1** has a methylated hydroxy group at position C-1 in ring A, a modification that can be found only in a few angucyclines, such as the chlorocyclinones [45]. In addition, the drastic structural feature of **1** is the presence of an aminated and, subsequently, the acetylated methyl group at position C-19, which has not been previously reported.

The biological activity of **1** and **2** was tested. Both compounds showed no significant antimicrobial and anticancer activity. In a CAS assay, compound **2** showed low iron-binding siderophore activity [46].

The NMR spectra of **2** contained less carbon and proton signals than anticipated regarding its observed exact mass. The ^1^H-NMR signal of H-21 (CH) indicated the presence of a mirror plane, since all other integrated proton signals were a multiple of one (2–6). Another strong indicator was the low number of carbon peaks observed in the ^13^C-NMR (24 peaks in ^13^C-NMR), which only sums up to the corresponding exact mass of **2** with an unreasonable number of incorporated heteroatoms. After the identification of the mirror plane, the monomer SEK43 (Figure S51) could easily be elucidated [47]. The protons H-16, H-18 and H-20 as well as H-6, H-8, H-9 and H-10 showed all necessary HMBC and ^1^H–^1^H–COSY correlations for the elucidation of their corresponding aromatic system. The connecting ketone moiety was elucidated through the correlations of H-18 and H-10 to C-13 and the carbon shift of C-13 (199.9 ppm). The pyrone moiety of SEK43 was elucidated through correlations between H-6 to C-5 and C-4, H-4 to C-1, C-2, C-4 and C-6, H-21 to C-1 and C-3 and H-22 to C-2. The connecting butyl group showed all expected ^1^H–^1^H–COSY and HMBC correlations (Figure S28). The putative axial stereochemistry of **2** can be assumed to be a racemic mixture due to its spontaneous formation as described below.

### 3.4. Both **1** and **2** Are the Result of Type II PKS Gene Cluster Expression

**Figure 3:**
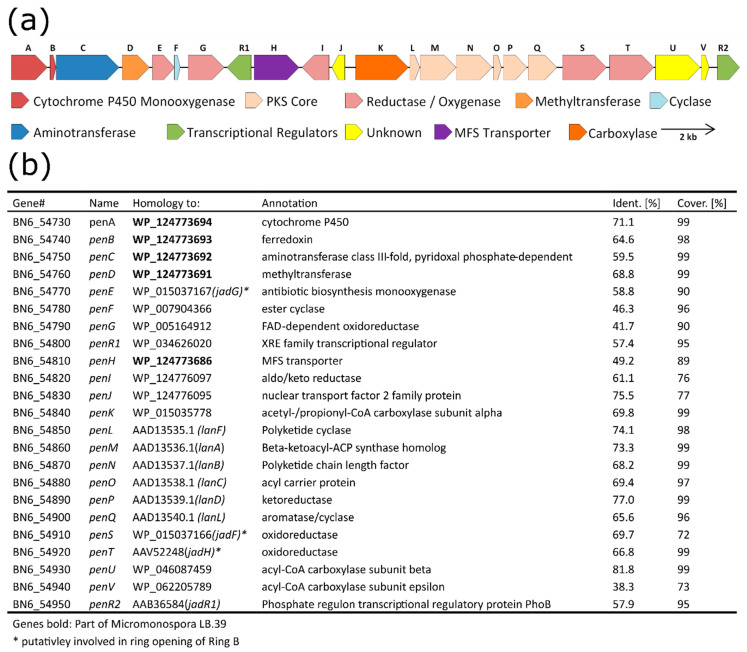
Schematic representation of the biosynthetic gene cluster of pentangumycin (**a**) and a BLAST analysis of the *pen* genes (**b**).

The antiSMASH analysis of the sequence of BAC 1E5 revealed the presence of three putative BGCs: a terpene cluster, a type-II PKS and a PKS–NRPS hybrid. From a structural perspective, it is obvious that neither **1** nor **2** are products of the terpene BGC and that **1** is a product of the type II polyketide synthase. However, **2** cannot easily be associated with the type II PKS or the PKS–NRPS hybrid. Even though the SEK43 moiety of **2** is a known shunt product of type II polyketide synthases, the connection through a butyl group has never been reported. To verify the biosynthetic origins of **1** and **2**, we aimed to identify the core genes of the corresponding BGCs and deleted them from BAC 1E5. A BLAST analysis of the type II PKS BGC showed that gene BN6_54860 is coding for a β-ketoacyl-synthase (Figure 3) and gene BN6_55110 is encoding a type I polyketide synthase in the PKS–NRPS BGC. Both genes were deleted separately in frame from BAC 1E5. The resulting BACs, 1E5Δ*54860* and 1E5Δ*55110*, were conjugated into *S. lividans* ΔYA6, and the metabolic profiles of both recombinant strains were analyzed. The production in the strain carrying mutant BAC 1E5Δ*55110* remained unchanged, whereas the deletion of gene BN6_54860 resulted in the loss of production of **1** and **2** (Figure S52). This clearly proves the origin of both compounds to be the type II PKS gene cluster.

### 3.5. Detailed Analysis and Border Prediction of the Biosynthetic Gene Cluster of Pentangumycin

The putative borders of pentangumycin’s BGC (MIBiG accession number: BGC0002070) are defined by the genes *BN6_54730* (further as *penA*) on the left edge and *BN6_54950* (further as *penR2*) on the right (Figure 3a). To predict their functions, individual pen genes were analyzed using BLASTx. Detailed results are illustrated in Figure 3b. The core of the *pen* cluster is formed by genes *penM, penN* and *penO*, coding for the minimal polyketide synthase. Together with two cyclase genes, *penL* and *penF*, the cyclase/aromatase *penQ* and the ketoreductase *penP* are forming a minimal set of genes required for the biosynthesis of the angucyclinone backbone. The polyketide core region is surrounded by seven genes putatively involved in oxidative or reductive reactions. The gene *penA* encodes a cytochrome P450 enzyme and is adjacent to *penB*, which encodes a ferredoxin protein. Both genes have previously been described as a functional unit [48,49,50]. The genes *penE*, *penG*, *penI*, *penS* and *penT* encode a monooxygenase (*penE*), three oxidoreductases (*penG, penI* and *penS*) and an aldo-/ketoreductase (*penT*). Except for PenE, PenS and PenT, the function of these enzymes cannot be predicted from the BLAST analysis. *penE*, *penS* and *penT* encode orthologues of JadG, JadF and JanH, respectively, which are three enzymes putatively involved in the oxidative opening of ring B of jadomycin [51]. Other genes such as *penC* (encoding putative aminotransferase) and *penD* (encoding putative methyltransferase) are also supposed to be involved in post-PKS modifications of **1**. *penK*, *penU* and *penV* encode α-, β- and ε-subunits of a carboxyltransferase, respectively. The function of these enzymes in the biosynthesis of **1** is unclear. Two regulatory genes—*penR1*, encoding a putative transcriptional regulator with the predicted helix-turn-helix motif at the N-terminus, and *penR2*, coding for a SARP protein—are present in the cluster [52]. PenR1 most probably controls the transcription of the outward-oriented *penH* (Figure 3), similar to the landomycins lanK, lanJ regulatory system [53,54,55]. The gene *penH* encodes a putative major facilitator superfamily transporter [56]. In turn, PenR2 shows a high degree of similarity compared to many well-studied SARP proteins from angucycline biosynthetic gene clusters, including JadR1 from *S. venezuelae* [52]. Due to the similar structures of **1** and landomycin and jadomycin [41,57], we aimed to identify homologues to genes involved in the biosynthetic pathways of both aforementioned compounds within the *pen* gene cluster. The PKS core genes *penL*, *penN*, *penO*, *penP* and *penQ* showed a significant similarity towards the landomycin biosynthetic genes *lanL*, *lanA*, *lanB*, *lanC* and *lanD*, respectively (Figure 3b). The oxygenase *penE* and the oxidoreductases *penS* and *penT* showed a remarkable homology to the oxidase *jadG* and the oxidoreductases *jadF* and *jadH*, respectively [58]. Additionally, a similarity between individual genes including *penA*, *penB*, *penC*, *penD* and *penH* including their organization and the putative biosynthetic gene cluster with unknown product from *Micromonospora sp.* LB39 was observed (Figure 3b and Figure S53).

We aimed to confirm the predicted borders (*penA* and *penR2*) of pentangumycins biosynthetic gene cluster. Therefore, two BACs (1E5_DEL_LEFT and 1E5_DEL_RIGHT) were designed. BAC 1E5_DEL_LEFT was constructed through deletion of 44.1 kb covering the left flanking region of the *pen* cluster upstream from the gene *penA* (Figure 3a), including the entire terpene cluster. The construction of 1E5_DEL_RIGHT was carried out in a similar way by deleting a 36.3-kb large region downstream to the *penR2* gene, including the entire predicted PKS–NRPS gene cluster. The constructed BACs were conjugated into *S. lividans* ΔYA6, and the production of **1** was analyzed. The recombinant strains carrying the mutated BACs still produced 1. Deletions of the terminal genes *penA* and *penR2* caused complete cessation of pentangumycin biosynthesis in the recombinant strains carrying corresponding BAC clones 1E5Δ*penA* and 1E5Δ*penR2*. The metabolic profiles of *S. lividans* transconjugants showed that both genes are required for production of **1**. These findings confirm that all genes necessary for the production of **1** are within the predicted borders of the *pen* gene cluster.

### 3.6. Biosynthesis of Pentangumycins Core Structure

**Figure 4:**
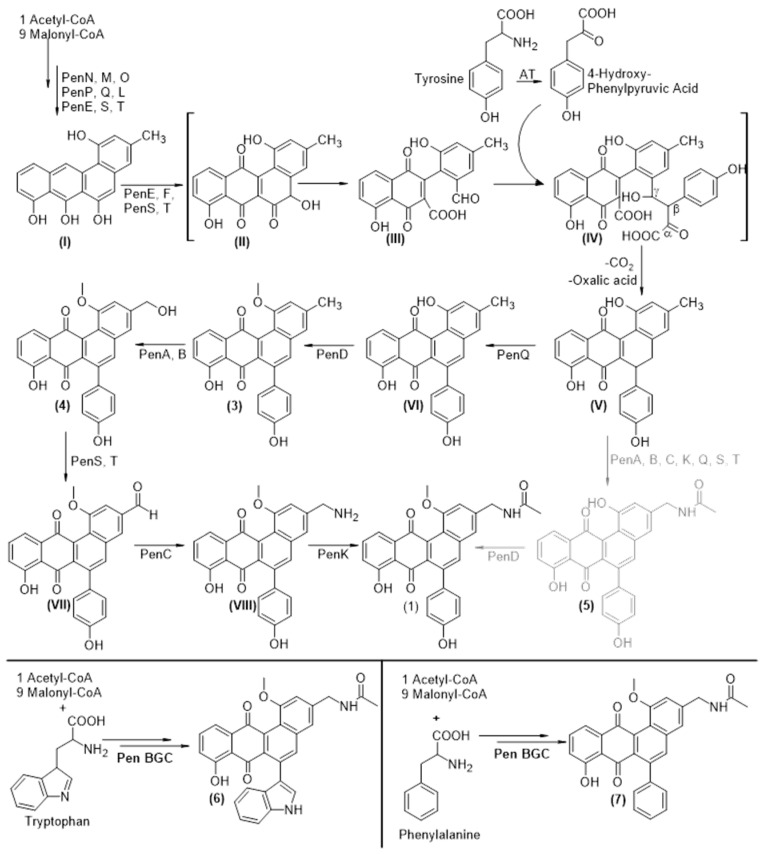
Biosynthetic pathway of pentangumycin; Roman numerals: proposed intermediates; Arabic numerals: intermediates with identified fitting masses after single gene knockouts and overexpression of the regulatory genes; Black pathway: methylation as first biosynthetic step after aromatization; Grey pathway: methylation as last biosynthetic step to complete the formation of pentangumycin (**1**).

Based on the available bioinformatic information described above (Figure 3b) and our experimental results, a biosynthetic route towards **1** was proposed (Figure 4) [59,60]. PenM, PenN and PenO utilize acetyl-CoA and malonyl-CoA to form the initial 20 carbon polyketide chain, which is reduced at position C-9 by PenP and cyclized by PenL and PenQ. Like in the case of all other angucycline type aromatic polyketides, the biosynthesis of **1** seems to proceed though the common intermediate UWM6 [57]. UWM6 is oxidized by PenE, PenS [60] and PenT, which leads to the formation of **I** [58]. The cleavage of ring B undergoes a Baeyer–Villiger oxidation through the putative intermediates **II** and **III**. The latter one is proposed to have an aldehyde functional group at position C-5 and a carboxyl group at position C-6. Similar to dauD in daunomycin biosynthesis, the ester cyclase PenF could perform a Knoevenagel condensation, which results in the formation of the C-C bond between the β-position of 4-hydroxyl-phenylpyruvic acid, an intermediate of the tyrosine degradation catalyzed by an aromatic amino acid transaminase [61], and the aldehyde of **III**, resulting in intermediate **IV**. An intramolecular nucleophilic reaction between the γ-hydroxyl and the α-keto groups cleaves oxalic acid and thus creates a double bond between the former β and γ positions and a conjugated system. The subsequent reformation of ring B is catalyzed by the decarboxylation at position C-5 and directed through the six-membered transition state towards the former β-position of the 4-hydroxyl-phenylpyruvic acid, leading to intermediate **V**. The aromatase/cyclase PenQ is proposed to be responsible for the aromatization of ring B, resulting in intermediate **VI**. To prove that the fifth ring of **1** derives from the incorporation of 4-hydroxyl-phenylpyruvic acid, a culture of *S. lividans* ΔYA6_1E5 was supplemented with fully labeled ^13^C-9-^15^N L-tyrosine. As a result, a mass shift to *m*/*z* 475 [M+H]^+^ was observed for the peak that corresponds to **1** (Figures S72–S77). This shift clearly shows that the fifth ring of **1** derives from incorporation of L-tyrosine, as it is in the case of jadomycin, and not from acetate, as it was shown for formicamycin [44]. Furthermore, the lack of two tyrosine-derived carbons in the final structure shows that the insertion of the fifth ring most probably occurs by the proposed mechanism with the loss of α- and β-carbons of tyrosine and the exchange of carbon six of **IV** with one of L-tyrosine’s in the re-cyclization process (Figure 4).

### 3.7. Tailoring Steps of Pentangumycin’s Biosynthesis

To elucidate the role of the remaining genes encoding the putative tailoring enzymes involved in post PKS modifications of **1**, a set of single knockout mutant BACs (1E5Δ*penA*; 1E5Δ*penC*; 1E5Δ*penD*; 1E5Δ*penE*, 1E5Δ*penG*; 1E5Δ*penI*; 1E5Δ*penJ*; 1E5Δ*penU*; 1E5Δ*penV*) was created and expressed in *S. lividans* ΔYA6. The production of **1** was not affected by deletions of *penG, penI, penJ, penU* and *penV* genes from BAC 1E5. At the same time, recombinant strains carrying 1E5 with deleted *penA*, *penC*, *penD* and *penE* genes lack the production of **1**, but were found (except *penE* mutant) to accumulate related angucyclinone compounds based on optical absorption and mass spectrometry data (Figure 4) The inactivation of *penA* resulted in the production of a new compound with *m*/*z* 411.1218 [M+H]^+^ that is very close to the calculated exact mass of 410.1154 Da of proposed intermediate **3** (Δ2.190 ppm) (Figure S78). The deletion of *penC* led to accumulation of the proposed intermediate **4** (detected *m*/*z* 427.1174 [M+H]^+^, calculated mass of 426.1103 Da, Δ0.409 ppm) (Figure S79). The BAC lacking *penD* gene facilitated the production of another compound with *m*/*z* 454.1278 [M+H]^+^ that corresponds to the mass of the proposed intermediate **5** (calculated exact mass of 453.1212, Δ1.506 ppm) (Figure S80). With the information gained from the analysis of all metabolic profiles from the recombinant strains carrying 1E5 with deletion of the aforementioned genes, it can be hypothesized that PenA catalyzes a hydroxylation of the methyl group at position C-19 to form a primary alcohol. The alcohol is subsequently oxidized by one of the oxidoreductases to an aldehyde, resulting in predicted intermediate **VII**. A similar biosynthetic reaction was proposed for the borrelidin biosynthetic pathway [62]. PenC performs an amination of the aldehyde at this position, resulting in structure **VIII**. However, in the extract of *S. lividans* ΔYA6_1E5ΔpenC, only proposed intermediate **4** can be found, most probably because the aldehyde of **VII** is not stable and is spontaneously reduced to an alcohol. Lastly, the deletion of *penD* leads to accumulation of **5**, the last intermediate before formation of **1** lacking methylation of the hydroxyl group at position C-1 (Figure 4). The presence of this intermediate, as well as methylated **3** and **4**, makes us believe that PenD can act on both substrates **5** and **VI** as well as that the presence of a methyl group at this position is not crucial for amination and acylation events.

The deletion of *penE* leads to a complete loss of production of **1**. Interestingly, the shunt product rabelomycin [63] can be identified in all of our extracts obtained from knockout mutants (identified with an external standard), but its production is drastically reduced in the extracts of *S. lividans* ΔYA6 1E5ΔpenE (Figure S81). This result indicates an involvement of *penE* in the early steps of the biosynthesis of **1**.

### 3.8. Analysis of Regulatory Gene Functions

To elucidate the functions of the identified regulatory genes on the biosynthesis of **1**, we constructed recombinant BACs with deletions of *penR1* and penR2. Both BACs were conjugated into *S. lividans* ΔYA6. The constructed recombinant strains *S. lividans* ΔYA6 1E5_Δ*penR1* and *S. lividans* ΔYA6 1E5_Δ*penR2* were analyzed for production of **1** (Figure S82). While **1** was still detectable in the ΔpenR1 mutant with a 50-fold decrease in yield, it was no longer present in the *S. lividans* ΔYA6 1E5_Δ*penR2* extracts. From this observation and high similarity of *penR2* to other SARP-encoding genes, it becomes obvious that this gene is encoding a pathway-specific regulator controlling the expression of structural pen genes. Furthermore, the overexpression of either *penR1* or *penR2* might lead to the increase in the production of **1**. To test this hypothesis, both genes were cloned under the control of the moderately strong A3 promoter [24] and introduced into *S. lividans* ΔYA6 using a marker-free vector system [64]. The resulting strains *S. lividans* ΔYA6_A3_*penR1* and *S. lividans* ΔYA6_A3_*penR2* were tested for the production of **1** and **2**. Surprisingly, the production level of **1** remained unchanged in both strains (Figure S83). *S. lividans* ΔYA6_A3_*penR1* 1E5 still produced **2**, but **2** was absent in extracts of *S. lividans* ΔYA6_A3_*penR2* 1E5 (Figure S84). These results indicate an interdependence between the two regulatory elements, similar to the promoter effects observed in the production of bottromycin, and lead to the conclusion that the unbalanced expression of pentangumycin’s BGC is indeed the reason for accumulation of SEK90 and its derivatives by the heterologous strain carrying BAC 1E5 [65]. Furthermore, two novel peaks, **6** and **7**, were detected in the extract of *S. lividans* ΔYA6_A3_*penR1* 1E5 (Figure S85). Based on LC-HRMS data, **6** could be proposed to be an analogue of **1** with tryptophan incorporated into ring B instead of tyrosine (detected *m*/*z* 491.15934 [M+H]^+^, calculated exact mass of 490.1529 Da, Δ1.646), while **7** seems to carry phenylalanine-derived moiety at the same position (detected *m*/*z* 452.14903 [M+H]^+^, calculated exact mass 451.1419 Da, Δ0.485 ppm) (Figure S85). To prove the possible nature of the identified derivatives **6** and **7**, the culture of *S. lividans* ΔYA6_A3_*penR1* 1E5 was supplemented with either L-tryptophan (^13^C_11_H_12_N_2_O_2_) or L-phenylalanine (ring-D_5_). As result, in the correspondingly fed cultures, the mass of **6** shifted to *m*/*z* 500.19 [M+H]^+^ (Figure S86), and the mass of **7** shifted to *m*/*z* 457.20 [M+H]^+^ (Figure S87), which corresponds to the incorporation of labelled L-tryptophan and labelled L-phenylalanine.

### 3.9. Origin of SEK90

**Figure 5:**
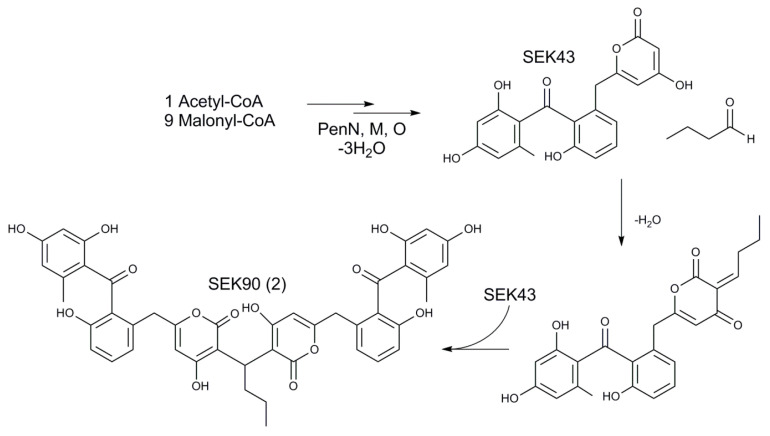
Origin of SEK90.

From a structural perspective, **2** is a dimer of SEK43 linked by a butyl group. It was shown by McDaniel that SEK43 is formed by spontaneous cyclization of the polyketide chain in mutants lacking the cyclase [47]. Since then, SEK43 has been reported as a shunt product in several studies of aromatic polyketide cyclases/aromatases [66,67,68]. At the same time, SEK43 was found in the extract of natural strains only in the case of the aranciamycin-producing *Streptomyces* sp. Tü6384 [69]. On the other hand, its derivative, named SEK43F, resulting from fusion of SEK43 with the pyrole-like moiety, was isolated from the recombinant *S. albus* expressing fluostatin biosynthetic gene cluster [70]. Nevertheless, a dimerization of this compound has not been described thus far. SEK43 has the 4-hydroxypyrone structural motif. The reactivity of 4-hydroxypyrones towards saturated aldehydes was reported previously [71]. It can be hypothesized that dimerization towards SEK90 can occur spontaneously inside the cell after the biosynthetic formation of SEK43 (Figure 5). To verify this theory, an experimental setup was designed; 6-Benzyl-4-hydroxy-2-pyrone that possesses the same structural motif as SEK43 was incubated with butanal or formaldehyde in distilled water, production medium inoculated with *S. lividans* ΔYA6 and production medium inoculated with *S. lividans* ΔYA6_1E5. If the dimerization of the pyrone with the corresponding aldehyde is performed by an enzymatic reaction, the expected dimers (1E5_CMP1, further designated as **8**, and 1E5_CMP2, further designated as **9**; see Figure S51) should only be present in the extracts of *S. lividans* ΔYA6 or *S. lividans* ΔYA6_1E5, depending on the presence of the necessary enzymes in the heterologous host or the introduced BAC. If the reaction towards **2** is spontaneous, the dimerization of pyrone and aldehydes should occur in the distilled water mixture. After incubation, all reactions were extracted and analyzed with LC-HRMS. Peaks that correspond to dimerized pyrones were present in all mixtures, including the distilled water-based one, confirming our hypothesis about the spontaneous nature of the 4-hydroxypyrone dimerization. Furthermore, we have synthesized **8** and **9** to verify their structure by NMR (Tables S6 and S7; Figures S54–S70) [71]. The peaks observed in the distilled water mixtures had identical exact masses and HPLC retention times as the synthesized standards (Figures S71 and S72). Detailed analysis of the *S. lividans* ΔYA6 1E5 extract revealed the presence of SEK43 (detected *m*/*z* 396.09679 [M+H]^+^, Δ0.242 ppm to SEK43 with the calculated exact mass of 368.0860 Da) (Figures S51 and S73) and a derivative of SEK90 connected by a methyl- instead of a butyl group named as SEK87 (detected *m*/*z* 749.1859 [M+H]^+^, Δ0.777 ppm to SEK87 with the calculated exact mass of 748.1792 Da) (Figures S51 and S74). None of the three compounds can be found in the extract of *S. espanaensis*. Thus, it is obvious that SEK90 as well as SEK87 derives from the combination of an unbalanced performance of pentangumycins minimal PKS, which seems to be uncoordinated with the activity of the Pen cyclases, leading to accumulation of SEK43 and the primary metabolism of *S. lividans*. A similar situation was proposed for the assembly of SEK43F, which resulted from interplay between the primary metabolism of the host strain *S. albus* and the heterologously expressed fluostatins type II PKS gene cluster [70].

## 4. Conclusions

In conclusion, we have shown that the described “cluster to compound” approach based on a systematic analysis of the genome of an Actinobacteria strain for unique BGCs combined with heterologous expression can be a source of new natural products with unique structural characteristics. As result, two novel compounds were isolated in sufficient amounts for structure elucidation and testing of their biological activity. Through genetic manipulation and feeding experiments, the biosynthesis of **1** was elucidated. SEK90 represents a new shunt product in biosynthesis of aromatic polyketides that arises from interplay between expressed BGC and primary metabolism of the host strain. At the same time, pentangumycin, the actual final product of the *pen* gene cluster is a new member of the angucyclinone family with a heavily modified ring system and a new, unusual tailoring modification which was not observed in other members of this group of natural products before.

Importantly, we have demonstrated that *Streptomyces* species can be used as hosts for heterologous expression of biosynthetic gene clusters derived from distantly related Actinobacteria species, even if their expression is silent in the wild-type strain. However, a still relatively low success rate should trigger the development of host strains other than *Streptomyces* for actinobacterial secondary metabolism gene cluster expression.

## Figures and Tables

**Table 1:** NMR spectroscopic data for pentangumycin (**1**) (DMSO-d_6_) and SEK90 (**2**) (DMSO-d_6_).

Pentangumycin (1)				SEK90 (2)			
Pos.	δ_C_	δ_H_ (*J* in Hz)	HMBC	Pos.	δ_C_	δ_H_ (*J* in Hz)	HMBC
1	156.7 C	-	-	1	166.5 C	-	-
2	108.7 CH	7.10 s	1, 4, 12b, 19	2	102.7 C	-	-
3	143.2 C	-	-	3	165.5 C	-	-
4	117.5 CH	7.44 s	1, 2, 4a, 5, 12b, 19	4	101.9 CH	5.68 (s br)	2, 5, 6
4a	136.6 C	-	-	5	160.6 C	-	-
5	135.1 CH	7.88 s	1, 4, 4a, 6a, 7, 12a, 12b, 13	6	36.1 CH_2_	3.55 (s br)	4, 5, 7, 8, 12
6	139.1 C	-	-	7	132.5 C	-	-
6a	131.2 C	-	-	8	120.1 CH	6.74 dd (7.7, N/A)	9, 10, 11, 12, 13
7	188.0 C	-	-	9	130.9 CH	7.2 dd (7.9, N/A)	7, 8, 10, 11, 12
7a	115.8 C	-	-	10	114.4 CH	6.77 dd (8.1, N/A)	8, 9, 11, 12, 13
8	160.5 C	-	-	11	153.3 C	-	-
9	123.0 CH	7.26 m	7a, 11	12	130.6 C	-	-
10	136.7 CH	7.73 t (7.5)	8, 11a	13	199.9 C	-	-
11	117.2 CH	7.50 dd (7.5, 0.93)	7a, 9, 10, 8, 12	14	115.3 C	-	-
11a	135.5 C	-	-	15	164.9 C	-	-
12	185.5 C	-	-	16	100.5 CH	6.11 d (2.4)	14, 15, 17
12a	140.0 C	-	-	17	166.3 C	-	-
12b	119.5 C	-	-	18	111.4 CH	6.04 dd (0.64, 2.4)	13, 14, 15, 16, 20
13	132.2 C	-	-	19	142.7 C	-	-
14/18	129.9 CH	7.26 m	6, 14/18, 16	20	21.2 CH_3_	1.8 s	14, 18, 19
15/17	114.8 CH	6.78 dt (8.5, 2)	13, 15/17, 16	21	29.5 CH	4.36 t (8.2)	1, 2, 3, 22, 23
16	156.6 C	**-**	-	22	31.5 CH_2_	1.75 dt (7.3, 8.2)	2, 21, 23, 24
19	42.4 CH_2_	4.43 d (6)	2, 3, 4, 20	23	20.6 CH_2_	1.05 dq (7.3)	21, 22, 24
20	169.6 C	-	-	24	13.8 CH_3_	0.89 t (7.3)	22, 23
21	22.8 CH_3_	1.93 s	20	-	-	-	-
22	56.1 CH_3_	3.88 s	1	-	-	-	-
NH	-	8.53 t (br)	19, 20	-	-	-	-
OH_1_	-	-	-	-	-	12.67 s	14, 15, 16
OH_2_	-	-	-	-	-	11.49 s (br)	-
OH_3_	-	-	-	-	-	10.38 s	16, 18
OH_4_	-	-	-	-	-	9.78 s	10, 11, 12

## Supplementary Materials

The following are available online at https://www.mdpi.com/2076-2607/8/12/2034/s1, The Supporting Information is available and contains supplementary figures and tables including HPLC-MS chromatograms, NMR spectra and additional structures.
