# Supplementary material for: Targeted Genome Mining—From Compound Discovery to Biosynthetic Pathway Elucidation
Source: Microorganisms. 2020 Dec 19;8(12):2034. doi: 10.3390/microorganisms8122034 (PMC7765855; doi:10.3390/microorganisms8122034)
Supplement: Supplementary file 1 [file microorganisms-08-02034-s001.pdf]

Supplementary

Targeted Genome Mining – From Compound Discovery to Biosynthetic Pathway Elucidation – Supplementary Materials

Nils Gummerlich [1], Yuriy Rebets [1], Constanze Paulus[1], Josef Zapp[2] and Andriy Luzhetskyy[1,3,*]

[1] Department of Pharmaceutical Biotechnology, Saarland University, Campus C2.3, 66123, Saarbrücken, Germany; nils.gummerlich@gmx.de (N.G.), yurko.rebets@gmail.com (Y.R.), constanzepaulus@gmail.com (C.P.)

[2] Department of Pharmaceutical Biology, Saarland University, C2.3, 66123 Saarbrücken, Germany; j.zapp@mx.uni-saarland.de (J.Z.)

[3] Helmholtz-Institute for Pharmaceutical Research Saarland (HIPS), Campus E8.1, 66123, Saarbrücken, Germany.

* Correspondence: a.luzhetskyy@mx.uni-saarland.de Tel.: +49-681-302-70200 (A.L.)

* Correspondence: andriy.luzhetskyy@helmholtz-hips.de; Tel.: +49-681-302-70200 (A.L.)

Received: date; Accepted: date; Published: date

List of tables

Table S 1 Oligonucleotides used in this work; All Oligonucleotides were produced by Eurofins (Eurofins Genomics Germany GmbH, Ebersberg, Germany) 9

Table S 2 Strains, Plasmids and BACs used in this work 12

Table S 3 Preparative HPLC method for pentangumycin (CMP468) & SEK90 (CMP791); 20 mL/min flow rate 14

Table S 4 AntiSMASH predicted biosynthetic gene clusters in *S. espanaensis* 16

Table S 5 NMR spectroscopic data of 1E5_CMP1 (*$CDCl_3$*) 17

Table S 3 NMR spectroscopic data of 1E5_CMP2 (*$CDCl_3$*) 18

Table S 7 Preparative HPLC method and yield for 8 and 9; Flow Rate: 20mL/min 15

List of Figures

Figure S 1 HPLC-MS Extracted ion chromatogram (Extracted mass 309±0.5) of *S. lividans* ΔYA6_3C18 and the corresponding mass chromatogram 19

Figure S2 HPLC-MS Extracted ion chromatogram (Extracted mass 468±0.5) of *S. albus* J1074_1E5 (up) and *S. lividans Δ6*_1E5 (down) 20

Figure S 3 HPLC-MS Extracted ion chromatogram; a) Extracted mass 468.00 – 468.20, Ethyl acetate extract of *S. espanaensis* grown in SG Medium; b) Extracted mass 791.10 – 791.30; Butanol extract of *S. espanaensis* grown in SG Medium 21

Figure S 4 Structure of Pentangumycin with all correlations (green: HMBC correlations H → C; blue: ^{1}H-^{1}H-Cosy correlation) 22

Figure S 5 ^{1}H-NMR spectrum (500 MHz, DMSO-d_6) of Pentangumycin; complete Spectrum and zoom from 1.7 to 0.1 ppm 23

Figure S 6 ^{1}H-NMR spectrum (500 MHz, DMSO-d_6) of Pentangumycin; complete Spectrum and zoom from 2.7 to 1.7 ppm 24

Figure S 7 ^{1}H-NMR spectrum (500 MHz, DMSO-d_6) of Pentangumycin; complete Spectrum and zoom from 3.65 to 2.65 ppm 25

Figure S 8 ^{1}H-NMR spectrum (500 MHz, DMSO-d_6) of Pentangumycin; complete Spectrum and zoom from 4.6 to 3.6 ppm 26

Figure S 9 ^{1}H-NMR spectrum (500 MHz, DMSO-d_6) of Pentangumycin; complete Spectrum and zoom from 5.6 to 4.6 ppm 27

Figure S 10 ^{1}H-NMR spectrum (500 MHz, DMSO-d_6) of Pentangumycin; complete Spectrum and zoom from 6.7 to 5.7 ppm 28

Figure S 11 ^{1}H-NMR spectrum (500 MHz, DMSO-d_6) of Pentangumycin; complete Spectrum and zoom from 7.6 to 6.7 ppm 29

Figure S 12 ^{1}H-NMR spectrum (500 MHz, DMSO-d_6) of Pentangumycin; complete Spectrum and zoom from 7.6 to 6.7 ppm 30

Figure S 13 ^{13}C-NMR (125 MHz, DMSO- d_6) of Pentangumycin; complete spectrum and zoom from 60 to 0 ppm. 31

Figure S 14 ^{13}C-NMR (125 MHz, DMSO- d_6) of Pentangumycin; complete spectrum and zoom from 130 to 70 ppm. 32

Figure S 15 ^{13}C-NMR (125 MHz, DMSO- d_6) of Pentangumycin; complete spectrum and zoom from 190 to 130 ppm. 33

Figure S 16 ^{1}H-^{1}H- COSY spectrum (500 MHz, DMSO-d_6) of Pentangumycin 34

Figure S 17 ^{1}H-^{1}H- COSY spectrum (500 MHz, DMSO-d_6) of Pentangumycin; Zoom to aromatic region 35

Figure S 18 ^{1}H-^{1}H- COSY spectrum (500 MHz, DMSO-d_6) of Pentangumycin; Zoom from 8.5 to 4.5 ppm 36

Figure S 19 HSQC-spectrum (500 MHz; 125 MHz, DMSO-d_6) of Pentangumycin; complete spectrum 37

Figure S 20 HSQC-spectrum (500 MHz; 125 MHz, DMSO-d_6) of Pentangumycin; zoom from 7.9 to 6.8 ppm and 139 to 107 ppm 38

Figure S 21 HSQC-spectrum (500 MHz; 125 MHz, DMSO-d_6) of Pentangumycin; zoom from 4 to 2 ppm and 59 to 19 ppm 39

Figure S 22 HMBC-spectrum (500 MHz; 125 MHz, DMSO-d_6) of Pentangumycin; complete spectrum 40

Figure S 23 HMBC - HSQC-Overlay spectrum (500 MHz; 125 MHz, DMSO-d_6) of Pentangumycin (black: HMBC; red/blue: HSQC); zoom from 9.2 to 6.5 ppm and 195 to 110 ppm 41

Figure S 24 HMBC - HSQC-Overlay spectrum (500 MHz; 125 MHz, DMSO-d_6) of Pentangumycin (black: HMBC; red/blue: HSQC); zoom from 9.2 to 6.5 ppm and 50 to 31 ppm 42

Figure S 25 HMBC - HSQC-Overlay spectrum (500 MHz; 125 MHz, DMSO-d_6) of Pentangumycin (black: HMBC; red/blue: HSQC); zoom from 4.6 to 3.5 ppm and 110 to 175 ppm 43

Figure S 26 HMBC - HSQC-Overlay spectrum (500 MHz; 125 MHz, DMSO-d_6) of Pentangumycin (black: HMBC; red/blue: HSQC); zoom from 4.6 to 3.7 ppm and 62 to 34 ppm 44

Figure S 27 HMBC - HSQC-Overlay spectrum (500 MHz; 125 MHz, DMSO-d_6) of Pentangumycin (black: HMBC; red/blue: HSQC); zoom from 2.6 to 1.4 ppm and 174 to 151 ppm 45

Figure S 28 Structure of SEK90 with all correlations (green: HMBC correlations H → C; blue: ^{1}H-^{1}H-Cosy correlation) 46

Figure S 29 ^{1}H-NMR spectrum (500 MHz, DMSO-d_6) of SEK90; complete Spectrum and zoom from 1.8 to 0.7 ppm 47

Figure S 30 ^{1}H-NMR spectrum (500 MHz, DMSO-d_6) of SEK90; complete Spectrum and zoom from 4.4 to 3.4 ppm 48

Figure S 31 ^{1}H-NMR spectrum (500 MHz, DMSO-d_6) of SEK90; complete Spectrum and zoom from 7.2 to 5.7 ppm 49

Figure S 32 ^{1}H-NMR spectrum (500 MHz, DMSO-d_6) of SEK90; complete Spectrum and zoom from 13 to 9.5 ppm 50

Figure S 33 ^{13}C-NMR spectrum (125 MHz, DMSO-d_6) of SEK90; complete Spectrum and zoom from 202 to 196 ppm 51

Figure S 34 ^{13}C-NMR spectrum (125 MHz, DMSO-d_6) of SEK90; complete Spectrum and zoom from 168 to 60 ppm 52

Figure S 35 13C-NMR spectrum (125 MHz, DMSO-d6) of SEK90; complete Spectrum and zoom from 155 to 113 ppm 53

Figure S 36 ^{13}C-NMR spectrum (125 MHz, DMSO-d_6) of SEK90; complete Spectrum and zoom from 105 to 92 ppm 54

Figure S 37 ^{13}C-NMR spectrum (125 MHz, DMSO-d_6) of SEK90; complete Spectrum and zoom from 37 to 20 ppm 55

Figure S 38 ^{13}C-NMR spectrum (125 MHz, DMSO-d_6) of SEK90; complete Spectrum and zoom from 26 to 6 ppm 56

Figure S 39 ^{1}H-^{1}H- COSY spectrum (500 MHz, DMSO-d_6) of SEK90........ 57

Figure S 40 ^{1}H-^{1}H- COSY spectrum (500 MHz, DMSO-d_6) of SEK90; zoom from 8 to 6.2 ppm 58

Figure S 41 ^{1}H-^{1}H- COSY spectrum (500 MHz, DMSO-d_6) of SEK90; zoom from 7 to 0.5 ppm 59

Figure S 42 HSQC-spectrum (500 MHz; 125 MHz, DMSO-d_6) of SEK90 60

Figure S 43 HSQC-spectrum (500 MHz; 125 MHz, DMSO-d_6) of SEK90; zoom from 7.5 to 5.5 ppm and 135 to 99 ppm 61

Figure S 44 HSQC-spectrum (500 MHz; 125 MHz, DMSO-d_6) of SEK90; zoom from 4 to 0,5 ppm and 53 to 16 ppm 62

Figure S 45 HSQC-spectrum (500 MHz; 125 MHz, DMSO-d_6) of SEK90; zoom from 4 to 0.5 ppm and 53 to 16 ppm 63

Figure S 46 HMBC - HSQC-Overlay spectrum (500 MHz; 125 MHz, DMSO-d_6) of SEK90 (red: HMBC; black/green: HSQC) 64

Figure S 47 HMBC - HSQC-Overlay spectrum (500 MHz; 125 MHz, DMSO-d_6) of SEK90 (black: HMBC; red/blue: HSQC); zoom from 13 to 10 ppm and 170 to 105 ppm........ 65

Figure S 48 HMBC - HSQC-Overlay spectrum (500 MHz; 125 MHz, DMSO-d_6) of SEK90 (black: HMBC; red/blue: HSQC); zoom from 13 to 10 ppm and 170 to 105 ppm........ 66

Figure S 49 HMBC - HSQC-Overlay spectrum (500 MHz; 125 MHz, DMSO-d_6) of SEK90 (black: HMBC; red/blue: HSQC); zoom from 6.5 to 0.5 ppm and 51 to 10 ppm........ 67

Figure S 50 HMBC - HSQC-Overlay spectrum (500 MHz; 125 MHz, DMSO-d_6) of SEK90 (black: HMBC; red/blue: HSQC); zoom from 4.5 to 0.5 ppm and 185 to 100 ppm........ 68

Figure S 51 Structures of SEK87, SEK43 and 1E5_CMP1 & 1E5_CMP2 69

Figure S 52 HPLC-MS Extracted ion chromatogram (Extracted mass 468±0.5) of a) S. lividans ΔYA6_1E5Δ54860 and b) S. lividans ΔYA6_1E5Δ55110; The production is unchanged in the deletion of the NRPS/PKSI hybrid core gene, while the production is abolished in the created mutant lacking the type II PKS gene........ 70

Figure S 53 Genes (WP_124773686 & WP_124773691-WP124773694) of Micromonospora sp. LB39 mapped to Pentangumycins biosynthetic gene cluster 71

Figure S 54 ^{1}H-NMR spectrum (500 MHz, $CDCL_3$) of 1E5_CMP1; zoom from 10.8 ppm to 10.5 ppm.. 72

Figure S 55 ^{1}H-NMR spectrum (500 MHz, $CDCL_3$) of 1E5_CMP1; zoom from 7.4 ppm to 7.2 ppm...... 73

Figure S 56 ^{1}H-NMR spectrum (500 MHz, $CDCL_3$) of 1E5_CMP1; zoom from 6 ppm to 3.5 ppm......... 74

Figure S 57 ^{1}H-^{1}H- COSY spectrum (500 MHz, $CDCL_3$) of 1E5_CMP1 75

Figure S 58 HSQC-spectrum (500 MHz; 125 MHz, $CDCL_3$) of 1E5_CMP1.. 76

Figure S 59 Structure of 1E5_CMP2 with all obsevered correlations (HMBC: green; ^{1}H-^{1}H-Cosy: blue) 77

Figure S 60 ^{1}H-NMR spectrum (500 MHz, $CDCL_3$) of 1E5_CMP2; zoom from 11.6 to 9.5 ppm 78

Figure S 61 ^{1}H-NMR spectrum (500 MHz, $CDCL_3$) of 1E5_CMP2; zoom from 7.4 to 7.2 ppm 79

Figure S 62 ^{1}H-NMR spectrum (500 MHz, $CDCL_3$) of 1E5_CMP2; zoom from 5.96 to 5.8 ppm 80

Figure S 63 ^{1}H-NMR spectrum (500 MHz, $CDCL_3$) of 1E5_CMP2; zoom from 4.25 to 3.75 ppm 81

Figure S 64 ^{1}H-NMR spectrum (500 MHz, $CDCL_3$) of 1E5_CMP2; zoom from 2.5 to 0.5 ppm 82

Figure S 65 ^{1}H-^{1}H- COSY spectrum (500 MHz, $CDCL_3$) of 1E5_CMP2 83

Figure S 66 HSQC-spectrum (500 MHz; 125 MHz, CDCL3) of 1E5_CMP2 84

Figure S 67 HMBC-spectrum (500 MHz; 125 MHz, CDCL3) of 1E5_CMP2 85

Figure S 68 HPLC-MS Extracted ion chromatogram (Extracted mass 417±0.5) of a) Spontaneously formed 8; b) synthesized 8; c) mass chromatogram of a)... 86

Figure S 69 HPLC-MS Extracted ion chromatogram (Extracted mass 459.1±0.1) of a) Spontaneously formed 9; b) synthesized 9; c) mass chromatogram of a)... 87

Figure S 70 HPLC Extracted ion chromatogram (Extracted mass 396.05-696.20) of *S. lividans* ΔYA6 1E5 b) mass of SEK43 88

Figure S 71 HPLC Extracted ion chromatogram (Extracted mass 749.15-749.22) of *S. lividans* ΔYA6 1E5 b) mass of SEK87 89

Figure S 72 HPLC-MS Extracted ion chromatogram (Extracted mass 468±0.5) of a) *S. lividans* ΔYA6_1E5 with 13C-9-15N-1-labelled L-tyrosine added to the medium b) MS-Chromatogram of a), incorporation of 13C-9-15N-1-labelled L-tyrosine visible; First repeat of a triplicate 90

Figure S 73 HPLC-MS Extracted ion chromatogram (Extracted mass 468±0.5) of a) *S. lividans* ΔYA6_1E5 with 13C-9-15N-1-labelled L-tyrosine added to the medium b) MS-Chromatogram of a), incorporation of 13C-9-15N-1-labelled L-tyrosine visible; second repeat of a triplicate...................... 91

Figure S 74 HPLC-MS Extracted ion chromatogram (Extracted mass 468±0.5) of a) *S. lividans* ΔYA6_1E5 with 13C-9-15N-1-labelled L-tyrosine added to the medium b) MS-Chromatogram of a), incorporation of 13C-9-15N-1-labelled L-tyrosine visible; Third repeat of a triplicate........................ 91

Figure S 75 HPLC-MS Extracted ion chromatogram (Extracted mass 468±0.5) of a) *S. lividans* ΔYA6_1E5 without 13C-9-15N-1-labelled L-tyrosine added to the medium b) MS-Chromatogram of a) no incorporation of 13C-9-15N-1-labelled L-tyrosine visible; first repeat of a triplicate 91

Figure S 76 HPLC-MS Extracted ion chromatogram (Extracted mass 468±0.5) of a) *S. lividans* ΔYA6_1E5 without 13C-9-15N-1-labelled L-tyrosine added to the medium b) MS-Chromatogram of a) no incorporation of 13C-9-15N-1-labelled L-tyrosine visible; second repeat of a triplicate 92

Figure S 77 HPLC-MS Extracted ion chromatogram (Extracted mass 468±0.5) of a) *S. lividans* ΔYA6_1E5 without 13C-9-15N-1-labelled L-tyrosine added to the medium b) MS-Chromatogram of a) no incorporation of 13C-9-15N-1-labelled L-tyrosine visible; third repeat of a triplicate 94

Figure S 78 HPLC-MS Extracted ion chromatogram (Extracted mass 411±0.5; corresponding mass to (3)) of a) *S. lividans* ΔYA6; b) *S. lividans* ΔYA6_1E5; c) *S. lividans* ΔYA6Δ*penA*; d) *S. lividans* ΔYA6Δ*penC; e) S. lividans* ΔYA6Δ*penD;* peak visible in c); f) ESI Full MS chromatogram of the peak visible in c) R_t=13.47 95

Figure S 79 HPLC-MS Extracted ion chromatogram (Extracted mass 427.09-427.18; corresponding mass to (4)) of a) *S. lividans* ΔYA6; b) *S. lividans* ΔYA6_1E5; c) *S. lividans* ΔYA6Δ*penA*; d) *S. lividans* ΔYA6Δ*penC; e) S. lividans* ΔYA6Δ*penD;* peak visible in d); f) ESI full MS of the peak visible in d) (R_t=10.22) 96

Figure S 80 HPLC-MS Extracted ion chromatogram (Extracted mass 454.10-454.17; corresponding mass to (5)) of a) *S. lividans* ΔYA6; b) *S. lividans* ΔYA6_1E5; c) *S. lividans* ΔYA6Δ*penA*; d) *S. lividans* ΔYA6Δ*penC; e) S. lividans* ΔYA6Δ*penD;* peak visible in e); f) f) ESI full MS of the peak visible in e) (R_t=12.02) 97

Figure S 81 HPLC-MS Extracted ion chromatogram (Extracted mass 339±0.5, Rabelomycin) of a) *S. lividans* ΔYA6 1E5, b) *S. lividans* ΔYA6 1E5ΔpenE c) pure rabelomycin as external standard 1

Figure S 82 HPLC-MS Extracted ion chromatogram (Extracted mass 468±0.5, (1)) of a) *S. lividans* ΔYA6 1E5, b) *S. lividans* ΔYA6 1E5ΔpenR1 and c) *S. lividans* ΔYA6 1E5ΔpenR2 2

Figure S 83 HPLC-MS Extracted ion chromatogram (Extracted mass 468±0.5 (1)) of a) *S. lividans* ΔYA6 1E5, b) *S. lividans* ΔYA6 A3_penR1 1E5 and c) *S. lividans* ΔYA6 A3_penR2 1E5 3

Figure S 84 HPLC-MS Extracted ion chromatogram (Extracted mass 791.10-791.25 (2)) of a) *S. lividans* ΔYA6 1E5, b) *S. lividans* ΔYA6 A3_penR1 1E5 and c) *S. lividans* ΔYA6 A3_penR2 1E5 4

Figure S 85 HPLC-MS Extracted ion chromatogram a) *S. lividans* ΔYA6 A3_penR1 (Extracted mass 491±0.5 (6) and 452±0.5 (7)), b) *S. lividans* ΔYA6 A3_penR1 1E5 (Extracted mass 452±0.5 (7)) c) *S. lividans* ΔYA6 A3_penR1 1E5 (Extracted mass 491±0.5 (6)) 5

Figure S 86 HPLC-MS Extracted ion chromatogram (Extracted mass 491±0.5) of a) *S. lividans* ΔYA6_A3_penR1 1E5 with L-tryptophan 13C-11 added to the medium b) MS-Chromatogram of a), incorporation of L-tryptophan 13C-11 visible 6

Figure S 87 HPLC-MS Extracted ion chromatogram (Extracted mass 452±0.5) of a) *S. lividans* ΔYA6_A3_penR1 1E5 with 2D-5-labelled L-Phenylalanine added to the medium b) MS-Chromatogram of a), incorporation of 2D-5-labelled L-Phenylalanine visible 7

Table S 1 Oligonucleotides used in this work; All Oligonucleotides were produced by Eurofins (Eurofins Genomics Germany GmbH, Ebersberg, Germany)

Name	Sequence of the Oligonucleotide
f3E7test	GTCGTTGACCCTCAGCAAGT
r3E7test	GCCTACCACTTCTACGCGTT
f1G5test	GTAGGCGAACAACTGCAACG
r1G5test	CTTCATGCGCAAGGAGATGC
f1C15test	ACCCGATCGGCCTCAAGATC
r1C15test	AGATCACGATCATCGCGGTC
f3K5test	GCGAAGTACGACACGACGTA
r3K5test	GGGGTGTCCGGTTCTACAAG
f1F6tes	ATGCTCAGGATCGAATCGCC
r1F6test	ACAGCTACGCCTTCGACTTC
f1C7test	GAATCATCGCCGACAGCAAC
r1C7test	GTTCTCGTCGATCTTGCCGA
f3E19test	AGCCTGGTCAACCACTGTTC
r3E19test	AGCCCAACAGTTGATAGCCC
f1G11test	CCTTCGAGATCTCCGGGTTG
r1G11test	GACTCGGGAACTCCTTACGC
f3C18test	GAGTACCACATCGCCACCAA
r3C18test	AGAGGATCTCCAGGTCGTCG
f1L8test	CCACTGACGTGACGCATACT
r1L8test	GTGAGCAGTGAGGTGTGGTT
f3M21test	AGACCGTCGAGCTGAACAAG
r3M21test	CACACCGTCTGGAGGATCTG
f1E5test	AAGCTGTGGTTGTACTCCGG
r1E5test	GCTTTCGGGGTCAATTGTCG
f3A24test	AGTTCGTGGAAGCGCTGGAC
r3A24test	AAGTCCTCCAGGACCTTCACC
f3K17test	GAAGACGTCGATGCTCCACA
r3K17test	ATCTTCGCCGGCTACAACAT
f-del-54860	CACCACGCCCCGGCACGACGACCCGGCCACCGCGTCACGGC TTCCGGGGATCCGTCGACCC
r-del-54860	GGGCCGCGCCAGCACCATGGCGCTCTGGAAGCCGCCGAAAC TGTAGGCTGGAGCTGCTTCG
f-del-55110	ATGGAGCCCATCGCGATCATCGGTGTGGGGTGCCGGTTCCC TTCCGGGGATCCGTCGACCC
r-del-55110	CGCGACCTCGCGCGGTGGTTCGTCACGACGCAGCGACGGCA TGTAGGCTGGAGCTGCTTCG
f-chk-54860	GCCTGCTTCGACGCGATCAA
r-chk-54860	GATCACGCCCATGTCGAACG
f-chk-55110	GATCACGCTCTCCCAGACG
r-chk-55110	GACGAGTCCTTCATCGCCAA
f-del-54730	GTGACCACCACAAGCCCCGCCTCCACGATCGACCTCTATTCCGACGAATTT CCGGGGATCCGTCGACCC
r-del-54730	TCAGCGGTGCAGGGTGGTCGGGAGCGCGGCGATGCCGCGGATGGTGTTGTG TAGGCTGGAGCTGCTTCG
f-del-54740	ATGAGGCTGATCGTCGATCGCGACCGCTGCGAAGGGCACGGCGTGTGCGTT CCGGGGATCCGTCGACCC
r-del-54740	TCAGCCTTCCAGCTTGAGGGCGGCGACCGGGCAGATGAGCACCGAGTCGT GTAGGCTGGAGCTGCTTCG

f-del-54750	ATGGAGAGCACGGCGCGAACGGTGTTCCGCCCGGCCTCGGCCCGCACCATTCCGGGGATCCGTCGACCC
r-del-54750	CTAGGACACCCTGGTCCGGGCCGCCCGGCCCTTGAGCAGCCGGCCCAGCTGTAGGCTGGAGCTGCTTCG
f-del-54760	ATGTCAGAGGAGAGCACCGAGAACACGACCGCCATGCGGTTGCTGCGGCTTCCGGGGATCCGTCGACCC
r-del-54760	CTAGGCGCGCACTGCTTCTACGACGCTGCACAGCCCGGCGGGGACGATCTGTAGGCTGGAGCTGCTTCG
f-del-54770+80	ATGCCGGTAATCGCGGCGAACGACGGTTGTCTGACCGTCTTCAACATGTTTCCGGGGATCCGTCGACCC
r-del-54770+80	TCAGTGCGGCGGGTCGACCAGCCCCAGTTGGACGAGGATGCCCATGTCGTGTAGGCTGGAGCTGCTTCG
f-del-54790	ATGAGCACACCCCCGTCACTGGTCGTCGTCGGCGCGTCCTTGGCCGGTCTTCCGGGGATCCGTCGACCC
r-del-54790	TCAGTCGCGGTGCACCGACGTGACCGCGTCGGACCAGGTCACGCCCCGGTGTAGGCTGGAGCTGCTTCG
f-del-54800	ATGATGGAAGGCGAACTGGGGGCCTTCCTGCGCAGTCGCCGTGAAGCCGTTCCGGGGATCCGTCGACCC
r-del-54800	TCAGCCGGAGCCGACCGCGCGCAACCCCACCTGGTTCACGTTCAGCCGGTGTAGGCTGGAGCTGCTTCG
f-del-54820	TCAGGCCGAGGCGCTGTCCAATCGGGACAGTTCCGCCGCAGTCAGCTCGTTCCGGGGATCCGTCGACCC
r-del-54820	ATGCGTCCGACTCCGCTGGGCACCGCCGCGCTGGGCACCACCGGGTTGATGTAGGCTGGAGCTGCTTCG
f-del-54830	TCAGGCGGGGTGCGGCGACCCGGTCGACCAGATCAGGTGGAACGACAGCTTCCGGGGATCCGTCGACCC
r-del-54830	ATGACAGCCGAGTCCGACCTGCCGTCCCGTGCCGACGTGGGCGCGCTGCTGTAGGCTGGAGCTGCTTCG
f-del-54930	ATCGAGGACGGCCCCGACCCGCAGGCCACCGAGCGCCAGCACGCCAAGGTTCCGGGGATCCGTCGACCC
r-del-54930	TCATTGCGGCGGGTTGCCGTGCTTGCGGCTGGGCAGGTCGGCGTGCTTGTGTAGGCTGGAGCTGCTTCG
f-del-54940	GTGCAAGGTGCATTCGTGGAGTTGCGATGCGTTGCCGACCGAATACCGGTTCCGGGGATCCGTCGACCC
r-del-54940	CTATTGCCAACTCGACGGGCTGCGGTAGGCCGTAACGCGTTCCGCGCGGTGTAGGCTGGAGCTGCTTCG
f-del-54950	GTGGTAGGGCAACAAGCCGGTGAGTTAGTCAGCACATGGGAGCCCGCGCTTCCGGGGATCCGTCGACCC
f-del-54950	TCAGCCCTCGCCGAGGCGGAAACCGACACCGCGAACGGTGACGATCCACTGTAGGCTGGAGCTGCTTCG
f-del-1E5-left	GTGGTGCCGCTCTCCCTCATAGGTTTGCCAACGACGGCGTCAGTGCGTCTTCCGGGGATCCGTCGACCC
r-del-1E5-left	CTGGCAGTGCCGGTCTCAGCTCGGCCTCCCTCACCGGCGACGGCTGCCATGTAGGCTGGAGCTGCTTCG
f-del-1E5-right	TCACCACGCTCCAAGGCGGAGCCGGGACGTGCCCGCCGCCCACGGCCGCTTCCGGGGATCCGTCGACCC
r-del-1E5-right	TCACGTCACGGTGAGGCGGATGGGTATGCCGAGGCTGGGGTTGCCCGGATGTAGGCTGGAGCTGCTTCG
f-del-54730-chk	TGAACTTCGGGACCTTGACG
r-del-54730-chk	CGATCGACGATCAGCCTCAT
f-del-54740-chk	GGTACAGCCGACCGACAC
r-del-54740-chk	ATCAGCTCGATGGTGCGG

f-del-54750-chk	ATGAGGCTGATCGTCGATCG
r-del-54750-chk	GTTCTCGGTGCTCTCCTCTG
f-del-54760-chk	CGCTGACGGTGTACAAGCA
r-del-54760-chk	TCCCGCATCTCTTTGAGCAG
f-del-54770+80-chk	TGGACCTCGACATGTTGGTC
r-del-54770+80-chk	TCCTCTCCTGGGTCCTGTTC
f-del-54790-chk	GAACAGGACCCAGGAGAGGA
r-del-54790-chk	GTTCGAGACGATGCAACTGG
f-del-54800-chk	TCCCCTACTTCTGGTCGGAC
r-del-54800-chk	GTGCTCGGTCATGTCTTCCC
f-del-54820-chk	CATCGGCGTTGTCGGAAAC
r-del-54820-chk	GTCGTTCCACCTGATCTGGT
f-del-54830-chk	GGTCGTGCAGGTAGTACAGG
r-del-54830-chk	GAGTGGGAGTTCTGCGACAA
f-del-54930-chk	GAAGGGAGCAGTGCGTGAA
r-del-54930-chk	CTTCTCCACCAGAACCTCCG
f-del-54940-chk	TGGTCCGGCAGTACAAAGAC
r-del-54940-chk	TTTTCCGGTGTCATGACGGT
f-del-54950-chk	TGCGGTTCACACTCTTGAGC
r-del-54950-chk	ATGTGGATGACTGCCCGTG
f-chk-1E5-le-int	GATCGCCATTGTGTCCGTTG
r-chk-1E5-le-int	CATCTCGATCACTCCCACGG
f-chk-1E5-ri-int	TCACAAGGCACCTTACGGAC
r-chk-1E5-ri-int	CATCAGCGAAATCACTGCCG
f-chk-1E5_ri	CTCCGGGAAGTGATACTCGC
r-chk-1E5_ri	GACGTTCTCCTCAGGTGACG
f-chk-1E5_le	CACTCGACGTCCAACTGTCA
r-chk-1E5_le	GAGTGCCGCGGATATCTGAA
F-54800A3OE	AAAAAAAGGTACCTAGCAGGGCTCCAAAACTAACGCCTGATGTAGGATCA GATGAAAAAAAAAAAGGAGGAAAATACATATGATGGAAGGCGAACTGGG
R-54800A3OE	AAAAAAAAAGCTTTCAGCCGGAGCCGACCGCGCGCAACCCCACCTGGTTC ACGTT
F-54950A3OE	AAAAAAAGGTACCTAGCAGGGCTCCAAAACTAACGCCTGATGTAGGATCA GATGAAAAAAAAAAAGGAGGAAAATACATGTGGTAGGGCAACAAGCC
R-54950A3OE	AAAAAAAAAGCTTTGTGGGTGTTCCACTCCGGA

Table S 2 Strains, Plasmids and BACs used in this work

Streptomyces	Characteristics	Reference
S. albus J1074	*S. albus G1 (DSM 41398)* derivative with the defective *SalG* I restriction modification system heterologous host	(Chater and Wilde, 1980 [1])
Streptomyces lividans Δ6	*S. lividans* TK24 derivative, deletion of 6 BGCs	(Paper in Revision)
Saccharothrix espanaensis DSM 44229 (T)	Producer of Saccharomicins	Labeda et al.[2]
**E. coli**	**Characteristics**	**Reference**
GB2005	General cloning host	Wang et al., 2006[3]
ET12567 pUZ8002	Strain used for intergeneric conjugation,	Kieser 2000[4]
GB2005-red-rham	*GB2005*, RhamC-BAD-γbaA used for Red/ET	Strochlic et al., 2010[5]
**Plasmids**	**Characteristics**	**Reference**
psmart-BAC-S	Apr^R; BAC Vector	Lucigen
pTOS	Apr^R; VWB-based *Streptomyces* integrative vector	Herrmann et al. (2012)[6]
pTOS_A3_R1	pTOS derivative; Integrated Gene 54800, influenced by A3 promotor cloned into the Kpn I and Hind III site	This work
pTOS_A3_R2	pTOS derivative; Integrated Gene 54950, influenced by A3 promotor cloned into the Kpn I and Hind III site	This work
patt-shyg	*Swa* I/*EcoR* V fragment of phyg-OK containing hyg cloned into the *EcoR* V site of patt	Myronovskyi et al. [7]
**BACs**	**Characteristics**	**Reference**
pSMART-BAC-S-1C15	pSMART-BAC-S derivative containing a fragment of the *S. espanaensis* chromosome (Locus in bp: 1,670,099-1,769,322)	Intact genomics
pSMART-BAC-S-3A24	pSMART-BAC-S derivative containing a fragment of the *S. espanaensis* chromosome (Locus in bp: 2,190,390-2,280,022)	Intact genomics
pSMART-BAC-S-3E7	pSMART-BAC-S derivative containing a fragment of the *S. espanaensis* chromosome (Locus in bp: 2,332,949-2,404,154)	Intact genomics
pSMART-BAC-S-1G5	pSMART-BAC-S derivative containing a fragment of the *S. espanaensis* chromosome (Locus in bp: 2,825,657-2,924,398)	Intact genomics
pSMART-BAC-S-1F6	pSMART-BAC-S derivative containing a fragment of the S. espanaensis chromosome (Locus in bp: 3,429,623-3,516,631)	Intact genomics
pSMART-BAC-S-1I20	pSMART-BAC-S derivative containing a fragment of the *S. espanaensis* chromosome (Locus in bp: 3,577,211-3,660,078)	Intact genomics
pSMART-BAC-S-3E19	pSMART-BAC-S derivative containing a fragment of the S. espanaensis chromosome (Locus in bp: 3,526,632-3,686,841)	Intact genomics
pSMART-BAC-S-3K5	pSMART-BAC-S derivative containing a fragment of the *S. espanaensis* chromosome (Locus in bp: 3,786,071-3,893,910)	Intact genomics

pSMART-BAC-S-1G11	pSMART-BAC-S derivative containing a fragment of the *S. espanaensis* chromosome (Locus in bp: 4,412,386-4,533,983)	Intact genomics
pSMART-BAC-S-3C18	pSMART-BAC-S derivative containing a fragment of the *S. espanaensis* chromosome (Locus in bp: 4,412,386-4,533,983)	Intact genomics
pSMART-BAC-S-1L8	pSMART-BAC-S derivative containing a fragment of the *S. espanaensis* chromosome (Locus in bp: 5,681,922-5,790,602)	Intact genomics
pSMART-BAC-S-3M21	pSMART-BAC-S derivative containing a fragment of the *S. espanaensis* chromosome (Locus in bp: 5,865,637-5,963,104)	Intact genomics
pSMART-BAC-S-1E5	pSMART-BAC-S derivative containing a fragment of the *S. espanaensis* chromosome (Locus in bp: 6,074,530-6,190,776)	Intact genomics
pSMART-BAC-S-1C7	pSMART-BAC-S derivative containing a fragment of the *S. espanaensis* chromosome (Locus in bp: 6,252,700-6,375,967)	Intact genomics
pSMART-BAC-S-3K17	pSMART-BAC-S derivative containing a fragment of the *S. espanaensis* chromosome (Locus in bp: 6,511,399-6,617,883)	Intact genomics
1E5Δ55110	pSMART-BAC-S-1E5 derivative; Knockout of gene BN6_55110	This work
1E5Δ54860	pSMART-BAC-S-1E5 derivative; Knockout of gene BN6_54860	This work
1E5ΔpenA	pSMART-BAC-S-1E5 derivative; Knockout of gene BN6_54730	This work
1E5ΔpenC	pSMART-BAC-S-1E5 derivative; Knockout of gene BN6_54750	This work
1E5ΔpenD	pSMART-BAC-S-1E5 derivative; Knockout of gene BN6_54760	This work
1E5ΔpenE/F	pSMART-BAC-S-1E5 derivative; Knockout of gene BN6_54770 & BN6_54780	This work
1E5ΔpenG	pSMART-BAC-S-1E5 derivative; Knockout of gene BN6_54790	This work
1E5ΔpenR1	pSMART-BAC-S-1E5 derivative; Knockout of gene BN6_54800	This work
1E5ΔpenI	pSMART-BAC-S-1E5 derivative; Knockout of gene BN6_54820	This work
1E5ΔpenJ	pSMART-BAC-S-1E5 derivative; Knockout of gene BN6_54830	This work
1E5ΔpenV	pSMART-BAC-S-1E5 derivative; Knockout of gene BN6_54940	This work
1E5ΔpenR2	pSMART-BAC-S-1E5 derivative; Knockout of gene BN6_54950	This work
1E5Δleft	pSMART-BAC-S-1E5 derivative; Deletion of left flanking region (Deletion locus in bp: 6,079,768 - 6,123,914; Deletion of 44,147 bp)	This work
1E5Δright	pSMART-BAC-S-1E5 derivative; Deletion of right flanking region (Deletion locus in bp: 6,148,356 6,184,748; Deletion of 36,393 bp)	This work

Table S 3 Preparative HPLC method for pentangumycin (CMP468) & SEK90 (CMP791); 20 mL/min flow rate

Method for 1			Method for 2			Waters AutoPurification System
Min.	%A	%B	Min.	%A	%B	Waters 2545 Binary Gradient module
0	95	5	0	95	5	Waters SFO (System Fluidics organizer)
2	95	5	2	95	5	Waters 2998 PAD (Photodiode array Detector)
16	3	97	16	3	97	Waters SQ-Detector-2
17	3	97	20	3	97	Waters 2767 Sample Manager
18	95	5	21	95	5	Nucleodur C18 Htec 250/4,6 C18 5 μM (analytical)
19	95	5	22	95	5	Nucleodur C18 Htec 250/21 C18 5 μM (preparative)

Biosynthetic pathway of pentangumycin; Roman numerals: proposed intermediates; Arabic numerals: intermediates with identified fitting masses after single gene knockouts and overexpression of the regulatory genes; Black pathway: methylation as first biosynthetic step after aromatization; Grey pathway: methylation as last biosynthetic step to complete the formation of pentangumycin (1).

Table S 4 Preparative HPLC method and yield for 8 and 9; Flow Rate: 20mL/min

Method for 1E5_CMP1 and 1E5_CMP2			Waters AutoPurification System	Purified compounds
Min.	%A	%B	Waters 2545 Binary Gradient module	8: 6 mg
0	95	5	Waters SFO (System Fluidics organizer)	9: 7 mg
1	95	5	Waters 2998 PAD (Photodiode array Detector)	
5	50	50	Waters 2767 Sample Manager	
15	5	95	Nucleodur C18 Htec 250/4,6 C18 5 μM (analytical)	
19	5	95	Nucleodur C18 Htec 250/21 C18 5 μM (preparative)	
20	95	5		
21	95	5		

Table S 5 AntiSMASH predicted biosynthetic gene clusters in *S. espanaensis*

#	Prediction:[a]	Predicted Cluster:[b]	Homology:[c]	BAC:[d]	Expressed:[e]
1	Terpene	Geosmin	100%	known	No
2	Lanthipeptide	Erythreapeptin	75%	homology	No
3	Terpene	Isorenieratene	42%	ex: 1C15	No
4	Bacteriocine			known	No
5	Furan	Asukamycin	30%	ex: 3A24	No
6	NRPS			ex: 3E7	No
7	Other	A54145	3%	not covered	No
8	NRPS	Myxochelin	50%	ex: 1G5	No
9	Indole	Frankiamicin	14%	ex: 1F6	No
10	Ladderane, NRPS	Skyllamycin	22%	ex: 1F6	No
11	Linear azole containing Peptides	A201A	6%	ex: 1I20	No
12	Type I PKS	Tylactone	6%	ex: 3E19	No
13	NRPS	Tyrobetaine	53%	ex: 3K5	No
14	NRPS			not covered	No
15	NRPS, Type I PKS	Kedarcidin	18%	not covered	No
16	Lanthipeptide	Kinamycin	5%	ex: 1G11	No
17	Oligosaccharide	Teicoplanin	4%	not covered	No
18	Melanin			known	No
19	NRPS, Type I PKS	Leinamycin	15%	not covered	No
20	NRPS, Type I PKS	Lavendiol	35%	ex: 3C18	Yes
21	Terpene	Isorenieratene	85%	homology	No
22	NRPS	Cyclomarin	13%	ex: 1L8	No
23	Aminoglycoside			ex: 3M21	No
24	NRPS	Ficellomycin	3%	ex: 3M21	No
25	RIPP	Anantin C	75%	homology	No
26	Terpene, Type II PKS, Type I PKS, NRPS	Fluostatin	23%	ex: 1E5	Yes
27	terpene,NRPS,T1PKS	Ficellomycin	27%	ex: 1C7	No
28	Oligosaccharide	Desosamine	22%	ex: 3K17	No
29	Terpene			excluded	No
30	Terpene	SF2575	6%	excluded	No
31	Lanthipeptide	Olimycin A	8%	excluded	No

a: Prediction: Predicted class of BGC;

b: Predicted cluster: Cluster with the highest homology;
c: Homology: Homology between the predicted cluster and the BGC identified in the genome of S. espanaensis;
d: Work: known: Cluster was known and excluded; homology: due to high homology of the whole cluster, the cluster was excluded; not covered: Cluster was not covered by our library; ex: “XXX” Cluster was covered by the BAC “XXX” and chosen for expression in our host;
e: Expressed: Indication of successful expression in our heterologous hosts

Table S 6 NMR spectroscopic data of 1E5_CMP1 (*$CDCl_3$*)

1E5_CMP1			
Pos.	δ_C	δ_H, m (*J* in Hz)	HMBC
1 & 1'	129,6	7,22 m	-
2 & 2'	128,8	7,33 m	-
3 & 3'	127,6	7,28 m	-
4 & 4'	120,8	5,90 s	-
5 & 5'	39,7	3,71 s	-
6	18,75	3,48 s	-
OH1 & OH1'	-	10,69 s	-

Table S 7 NMR spectroscopic data of 1E5_CMP2 (*$CDCl_3$*)

1E5_CMP2			
Pos.	δ_C	δ_H, m (*J* in Hz)	HMBC
1 & 1'	170,29	-	-
2 & 2'	168,58	-	-
3 & 3'	163,33	-	-
4 & 4'	134,5	-	-
5 & 5'	129,23	7,22 m	C-10
6 & 6'	128,86	7,33 m	C-4
7 & 7'	127,44	7,28 m	C-5
8 & 8'	104,95	-	-
9 & 9'	102,83	5,86 m	C-10, C-8, C-3, C-2
10 & 10'	39,8	3,76 d (7,1 Hz)	C-9, C-5, C-4, C-3
11	31,42	4,13 t (7,6 Hz)	C-13, C-12, C-8, C2, C-1
12	30,13	2,16 m	C-14, C-13, C-11, C-8
13	21,83	1,25 m	C-14, C-12, C-11
14	13,74	0,90 t (7,4 Hz)	C-13, C-12
OH1	-	11,37 s	-
OH1'	-	10,55 s (br)	-

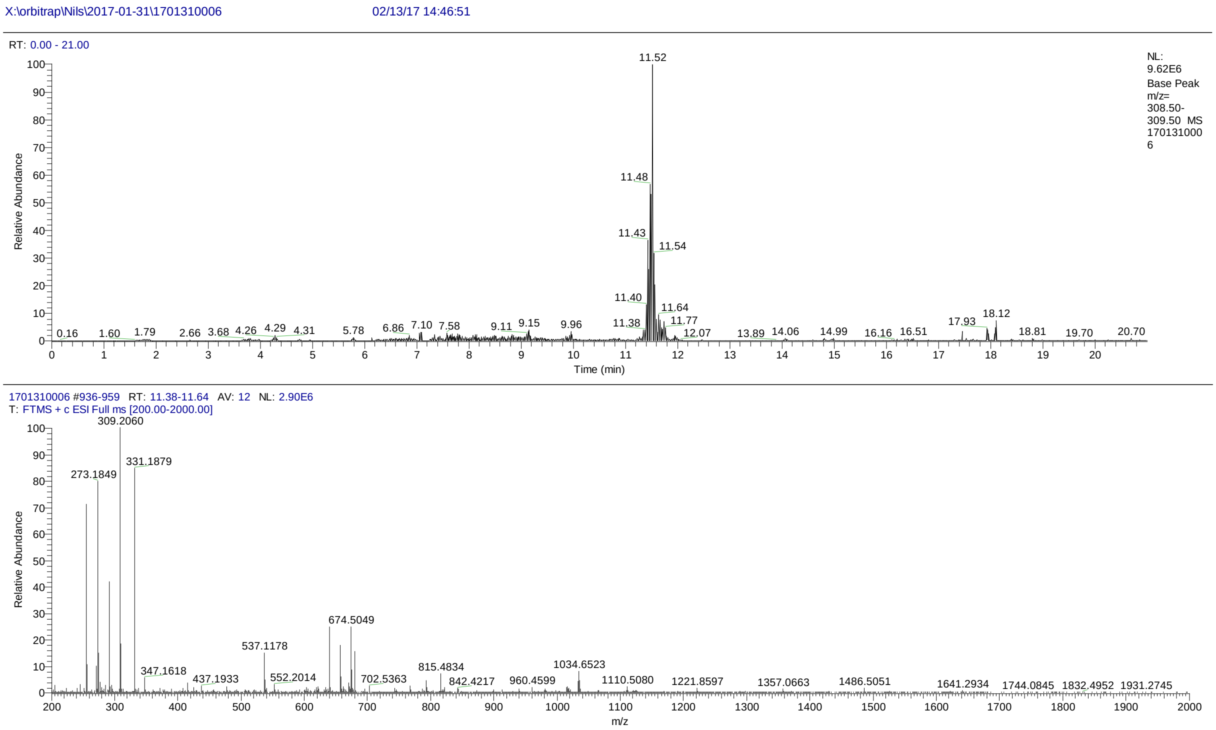

Figure S 1 HPLC-MS Extracted ion chromatogram (Extracted mass 309±0.5) of *S. lividans* ΔYA6_3C18 and the corresponding mass chromatogram

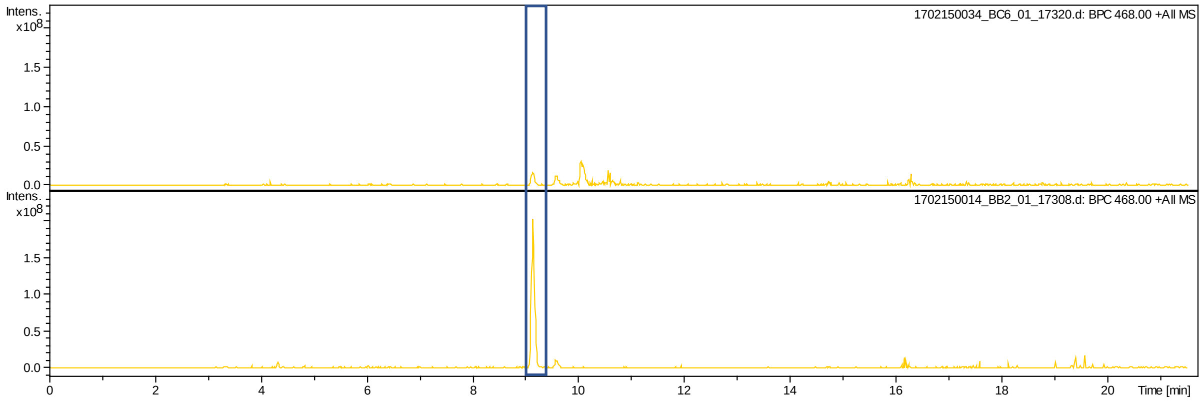

Figure S2 HPLC-MS Extracted ion chromatogram (Extracted mass 468±0.5) of *S. albus* J1074_1E5 (up) and *S. lividans Δ6*_1E5 (down)

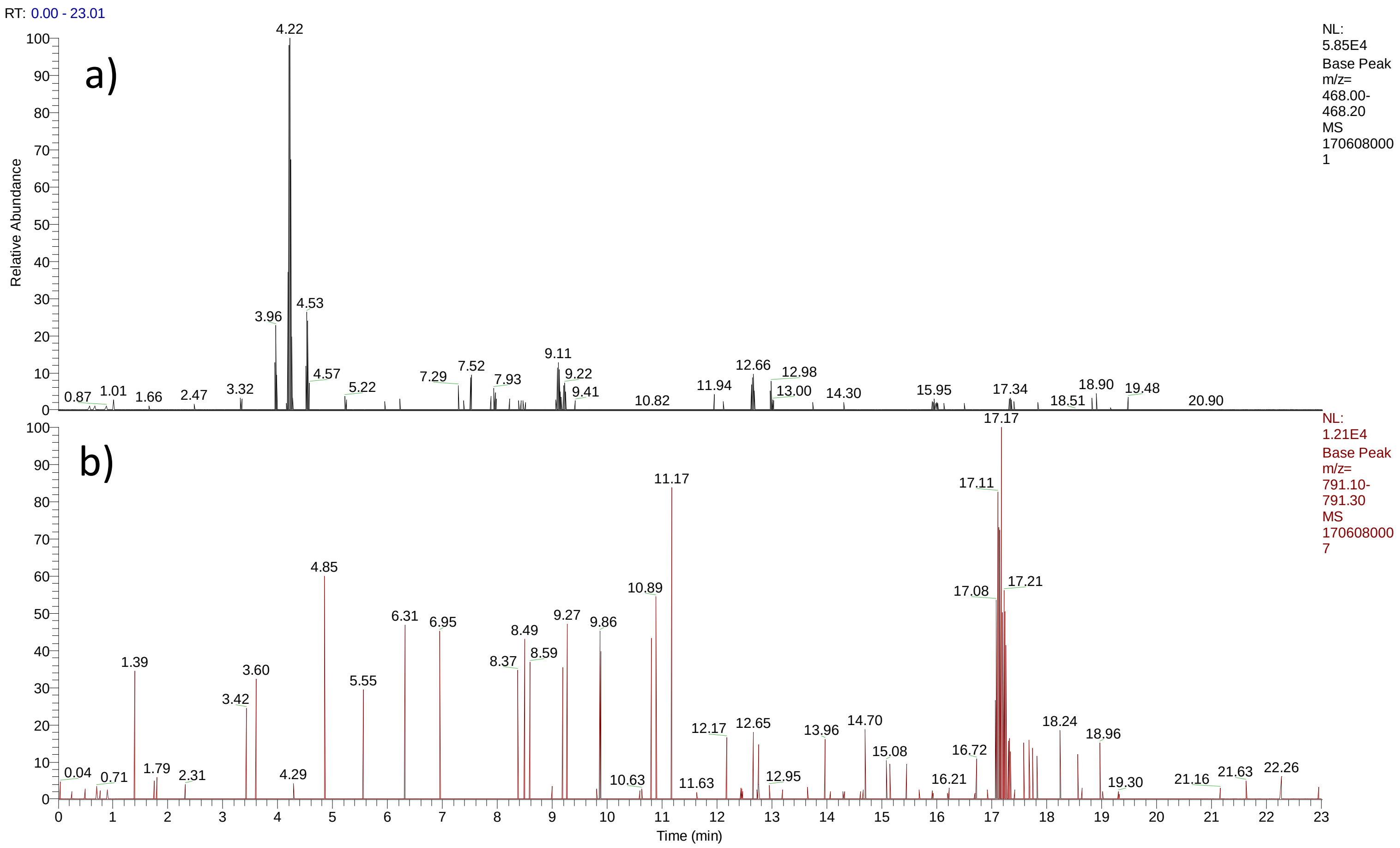

Figure S 3 HPLC-MS Extracted ion chromatogram; a) Extracted mass 468.00 – 468.20, Ethyl acetate extract of *S. espanaensis* grown in SG Medium; b) Extracted mass 791.10 – 791.30; Butanol extract of *S. espanaensis* grown in SG Medium

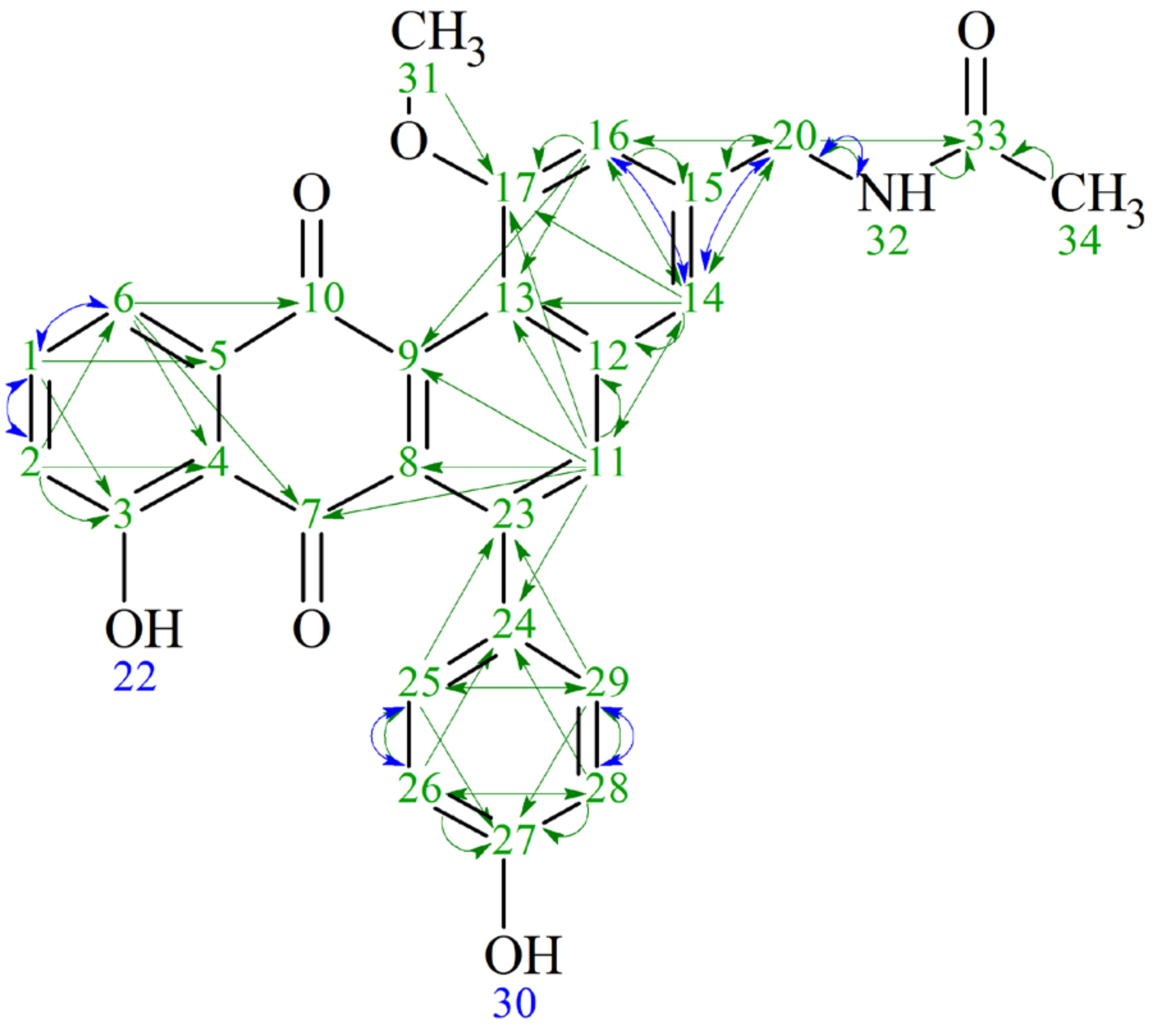

Figure S 4 Structure of Pentangumycin with all correlations (green: HMBC correlations H → C; blue: ^{1}H-^{1}H-Cosy correlation)

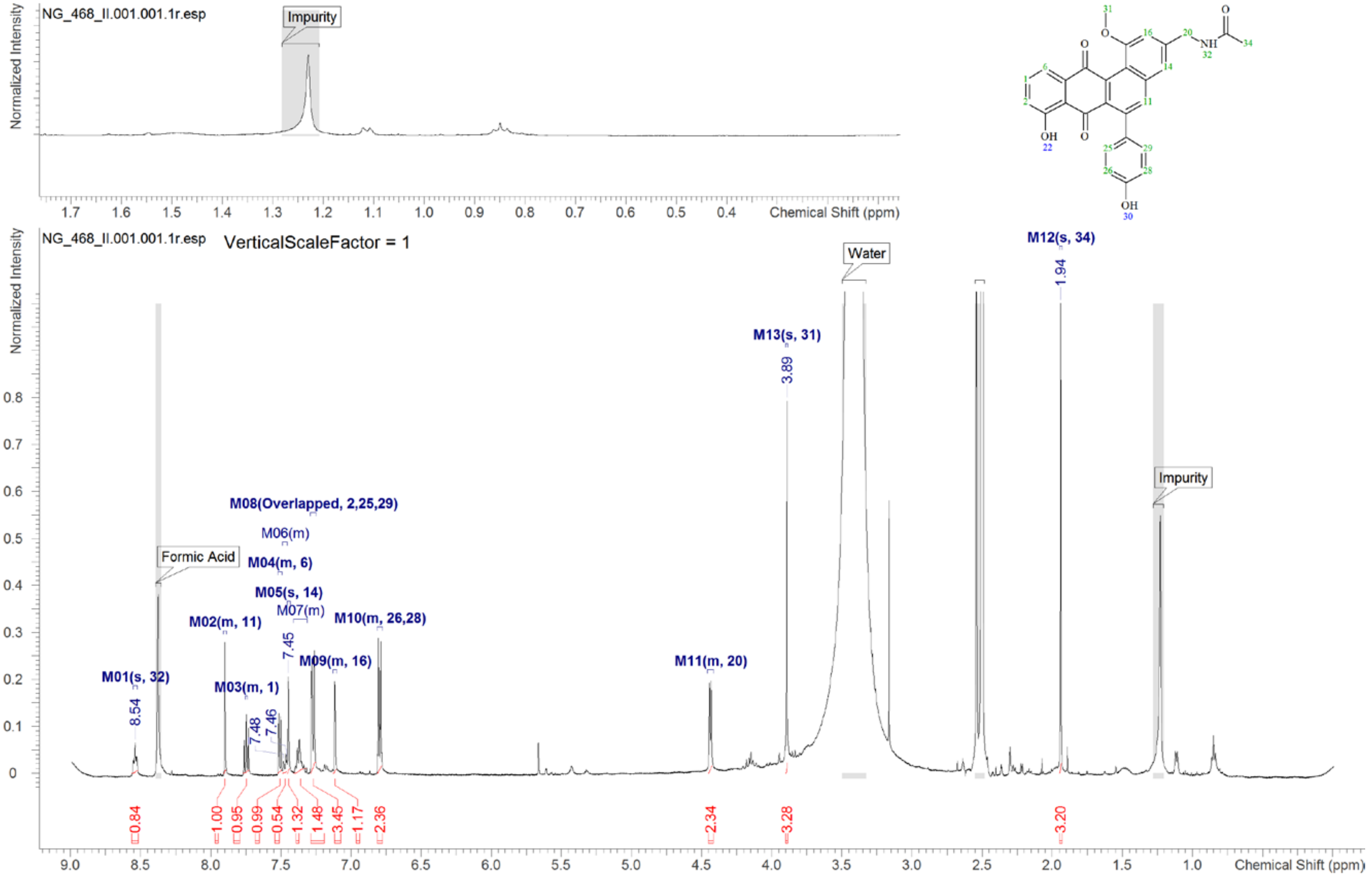

Figure S 5 ^{1}H-NMR spectrum (500 MHz, DMSO-d$_6$) of Pentangumycin; complete Spectrum and zoom from 1.7 to 0.1 ppm

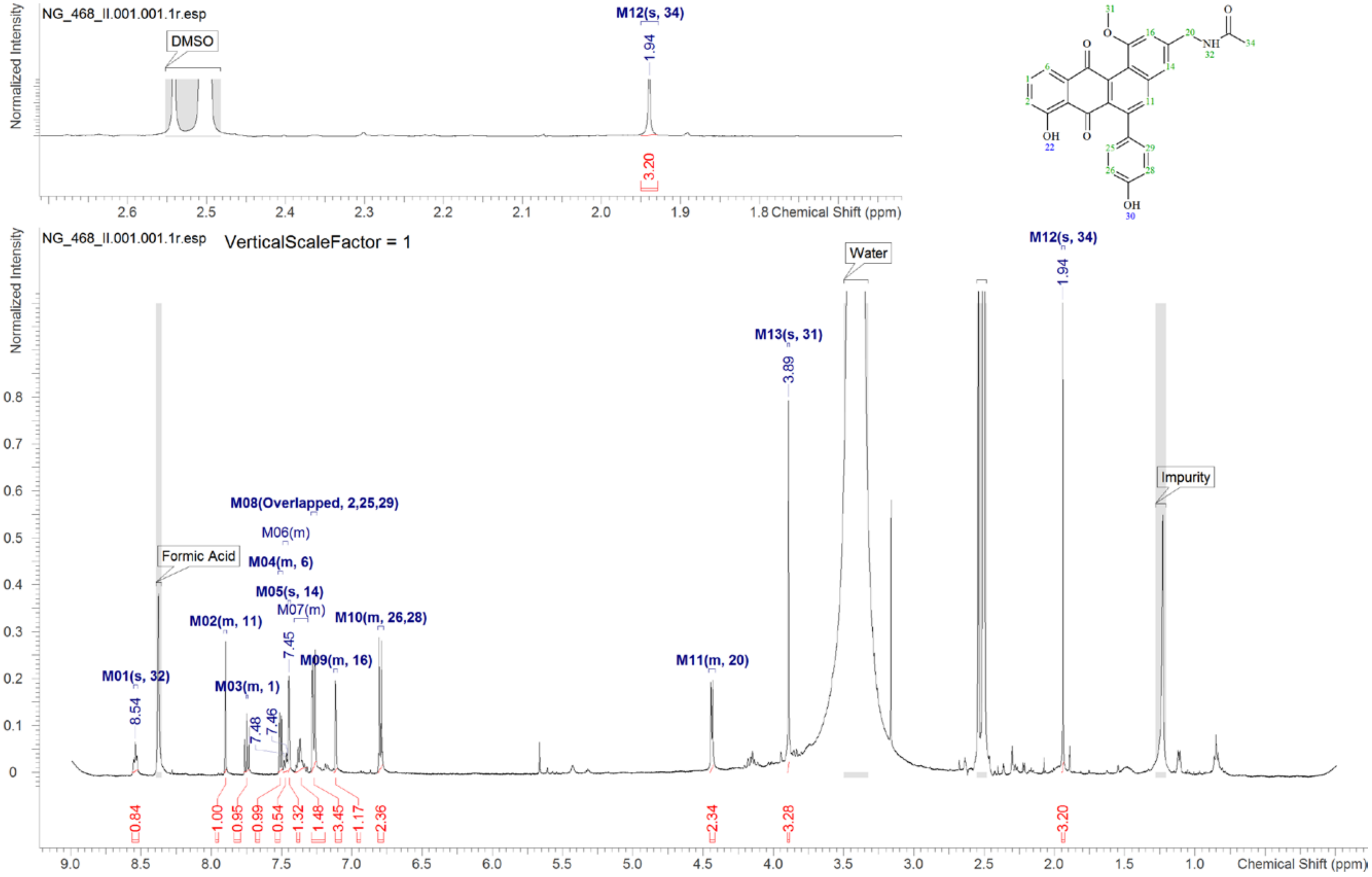

Figure S 6 ^{1}H-NMR spectrum (500 MHz, DMSO-d$_6$) of Pentangumycin; complete Spectrum and zoom from 2.7 to 1.7 ppm

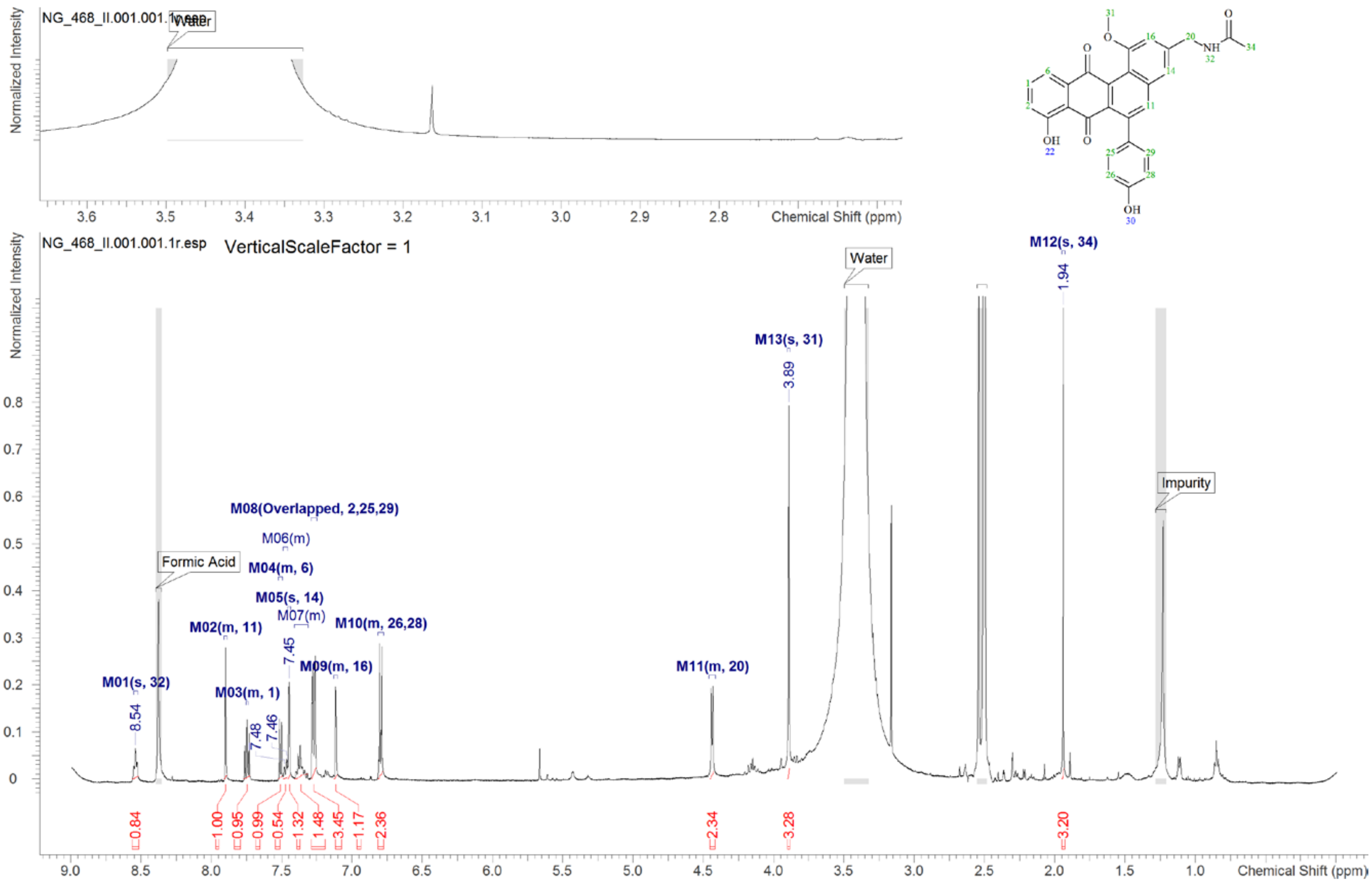

Figure S 7 ¹H-NMR spectrum (500 MHz, DMSO-d_6) of Pentangumycin; complete Spectrum and zoom from 3.65 to 2.65 ppm

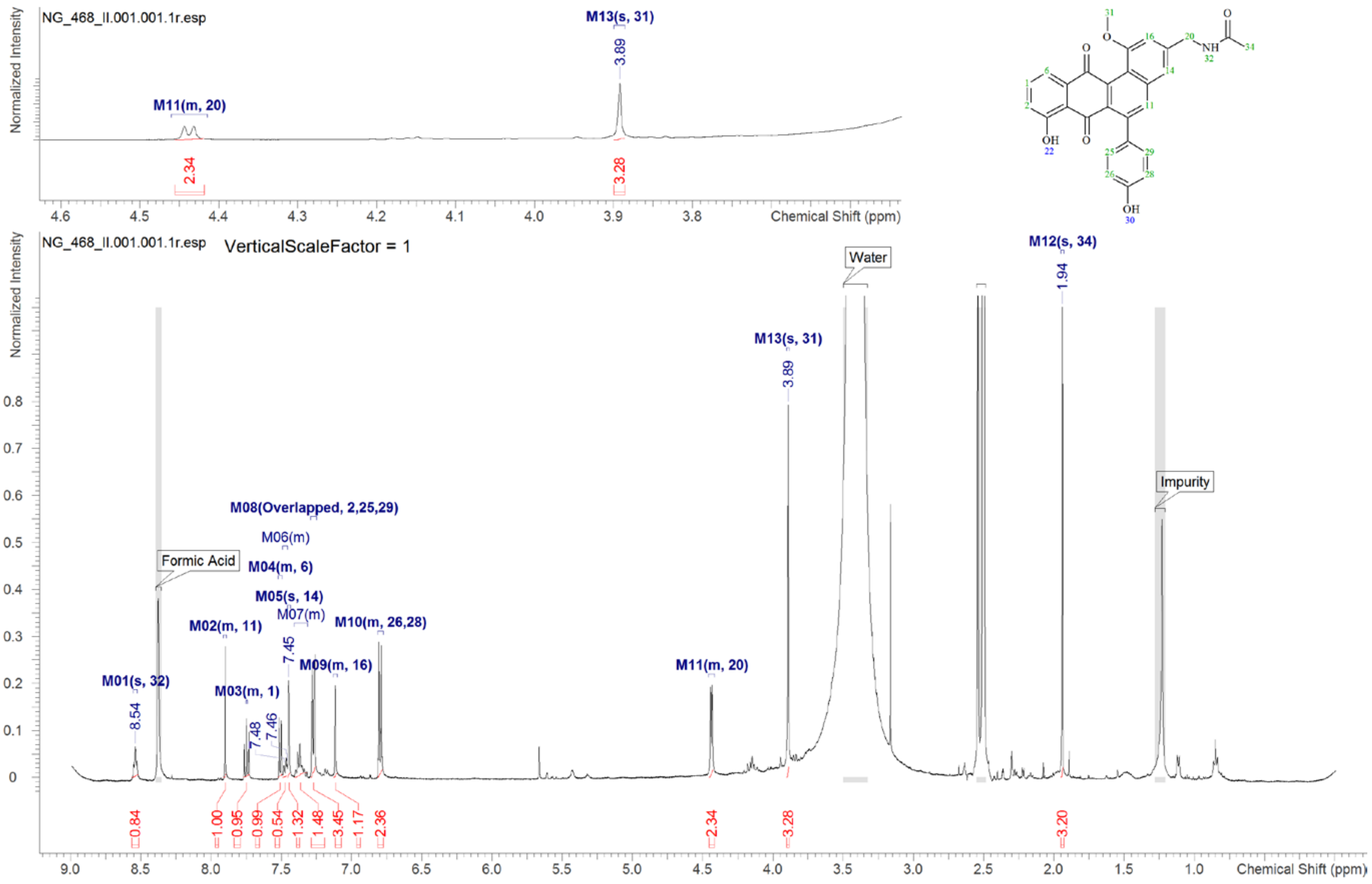

Figure S 8 ^{1}H-NMR spectrum (500 MHz, DMSO-d_6) of Pentangumycin; complete Spectrum and zoom from 4.6 to 3.6 ppm

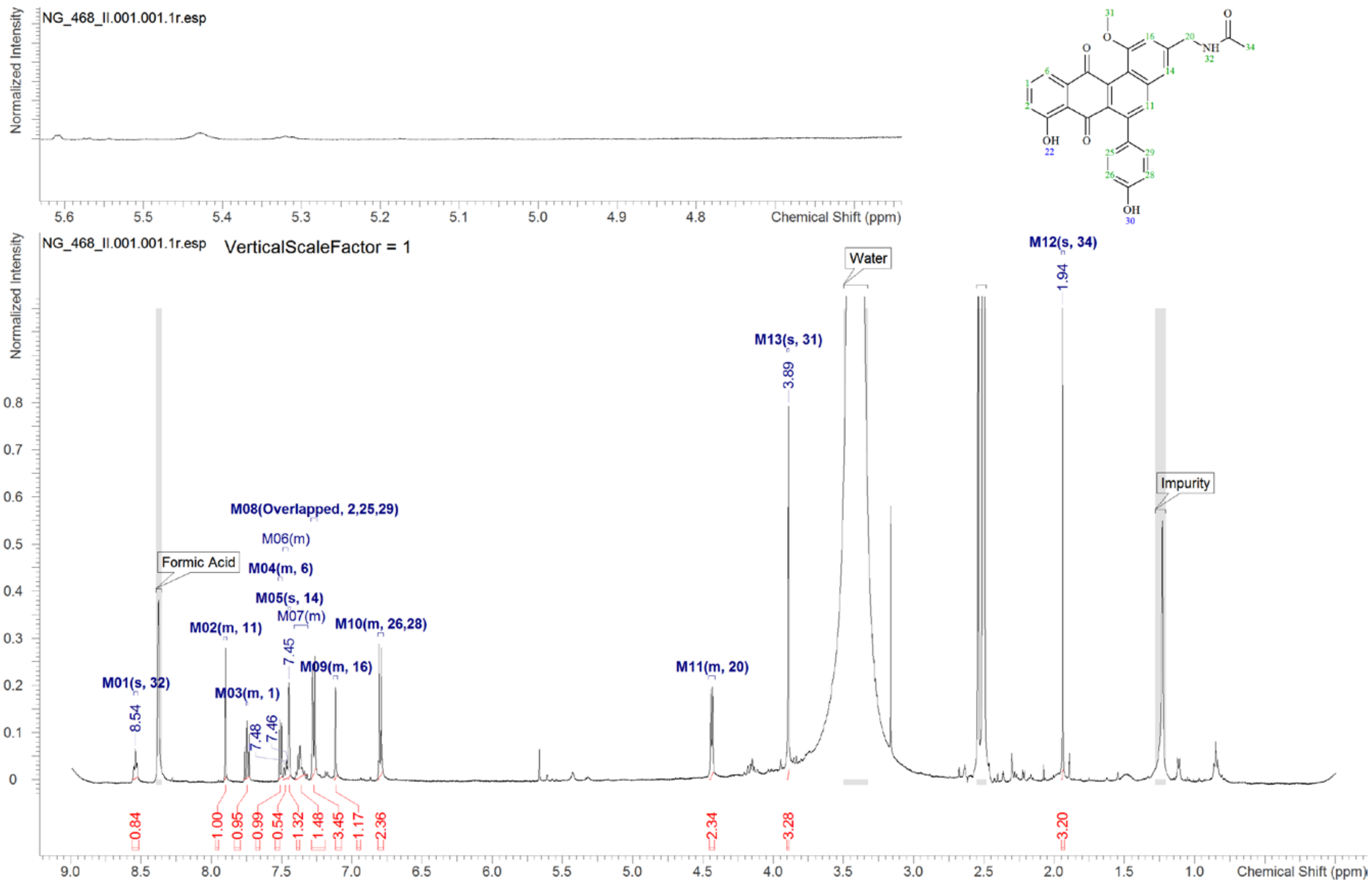

Figure S 9 ^{1}H-NMR spectrum (500 MHz, DMSO-d$_6$) of Pentangumycin; complete Spectrum and zoom from 5.6 to 4.6 ppm

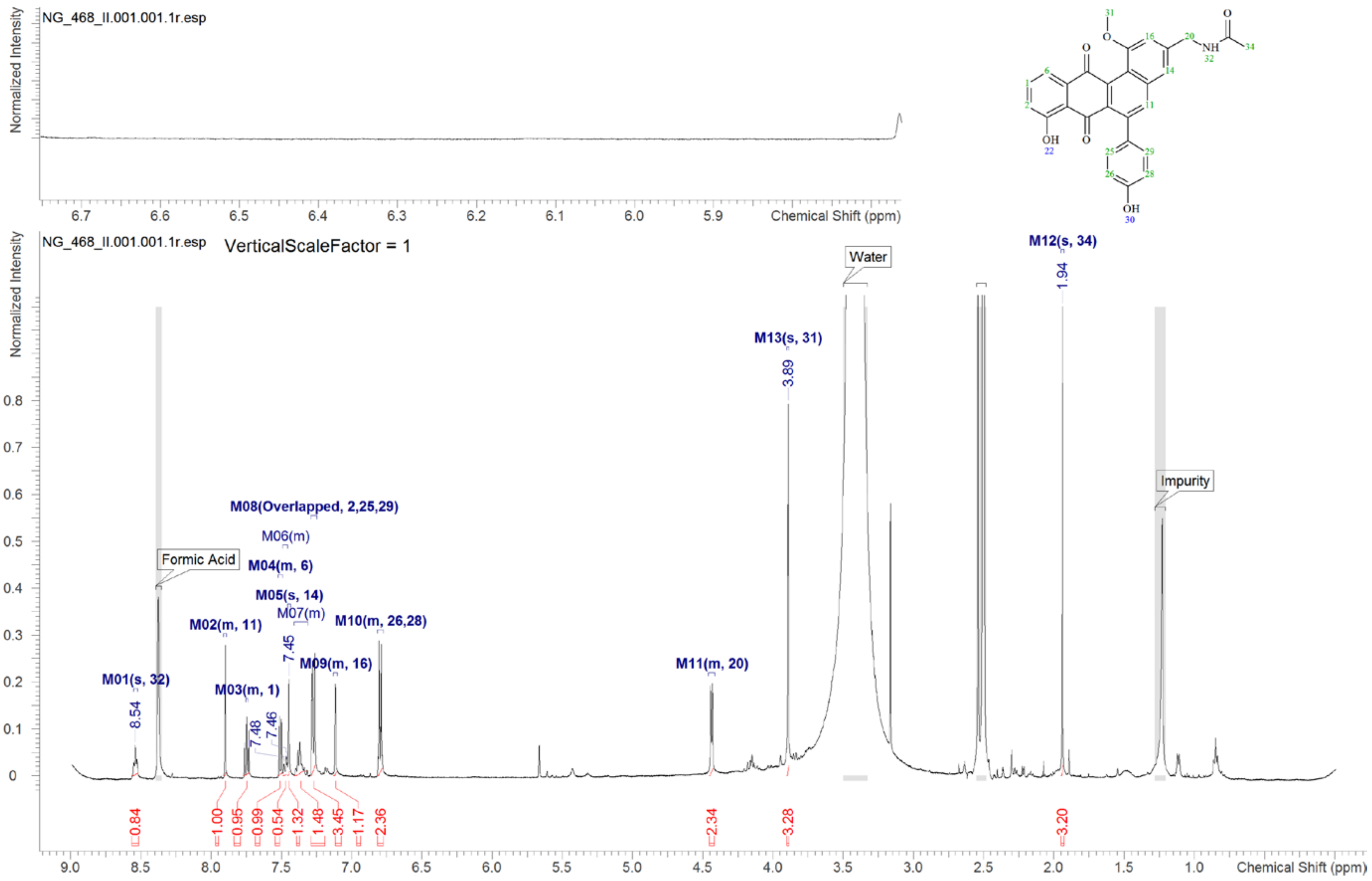

Figure S 10 ^{1}H-NMR spectrum (500 MHz, DMSO-d$_6$) of Pentangumycin; complete Spectrum and zoom from 6.7 to 5.7 ppm

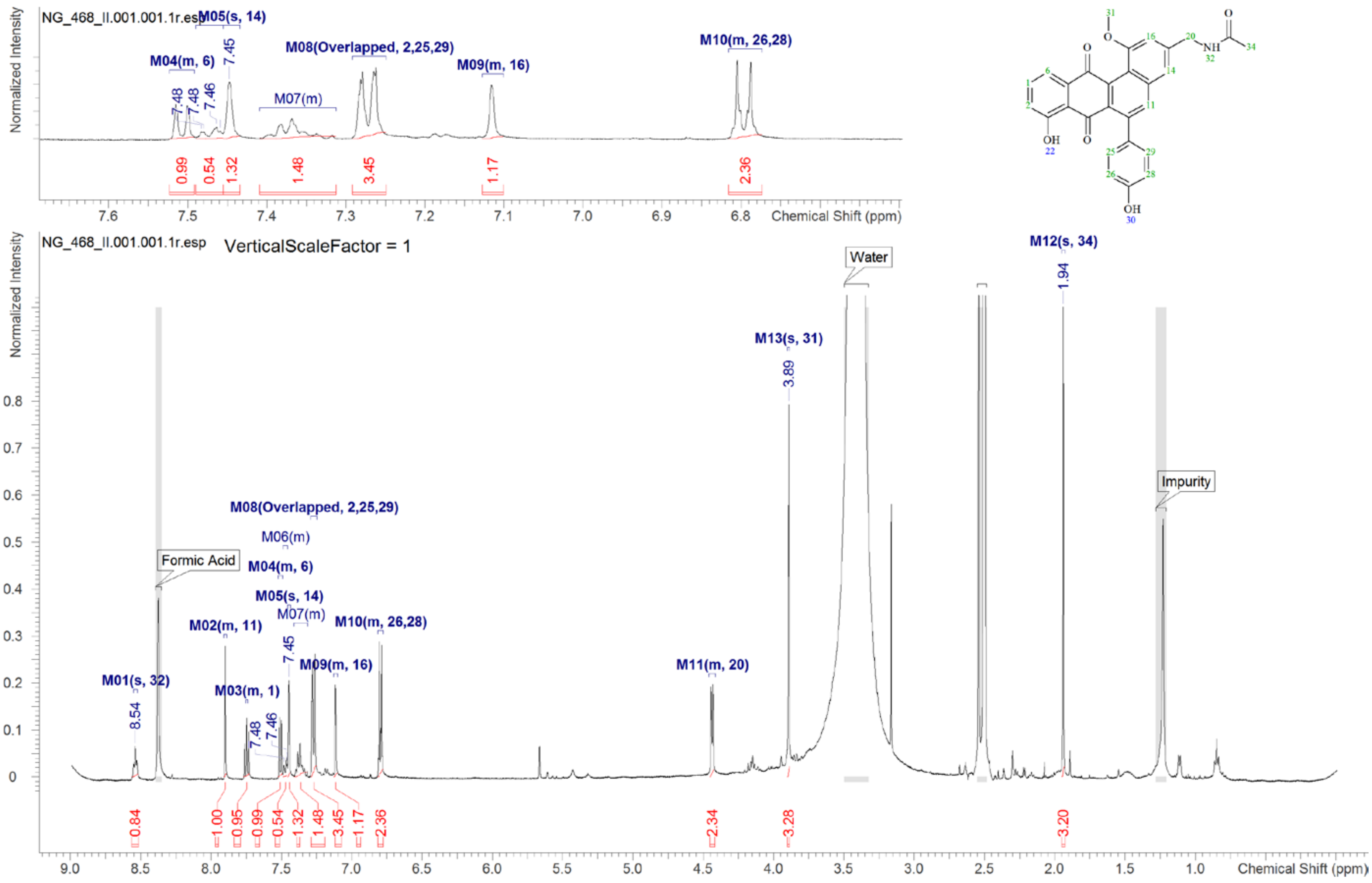

Figure S 11 ^{1}H-NMR spectrum (500 MHz, DMSO-d_6) of Pentangumycin; complete Spectrum and zoom from 7.6 to 6.7 ppm

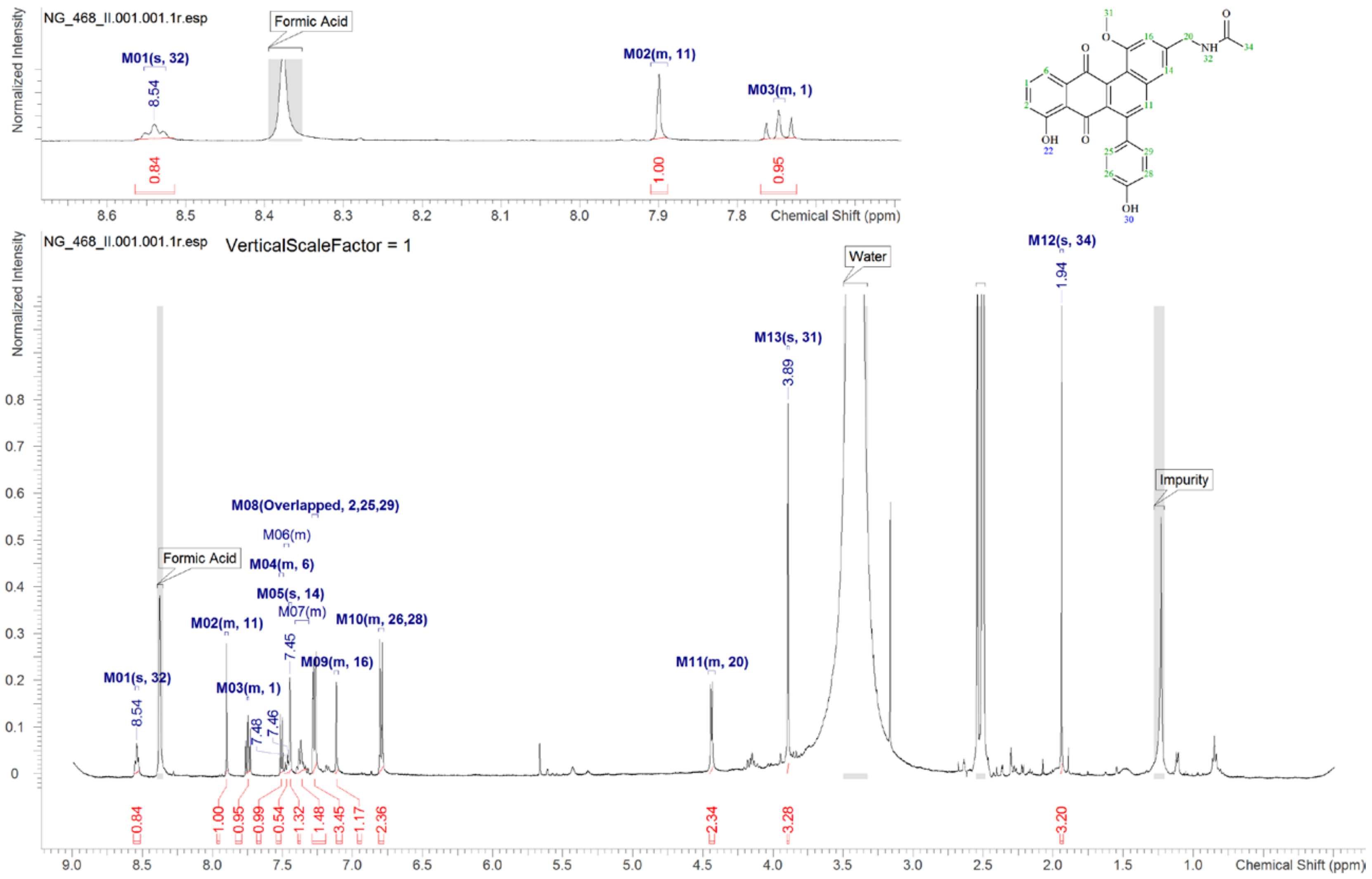

Figure S 12 ^{1}H-NMR spectrum (500 MHz, DMSO-d$_6$) of Pentangumycin; complete Spectrum and zoom from 7.6 to 6.7 ppm

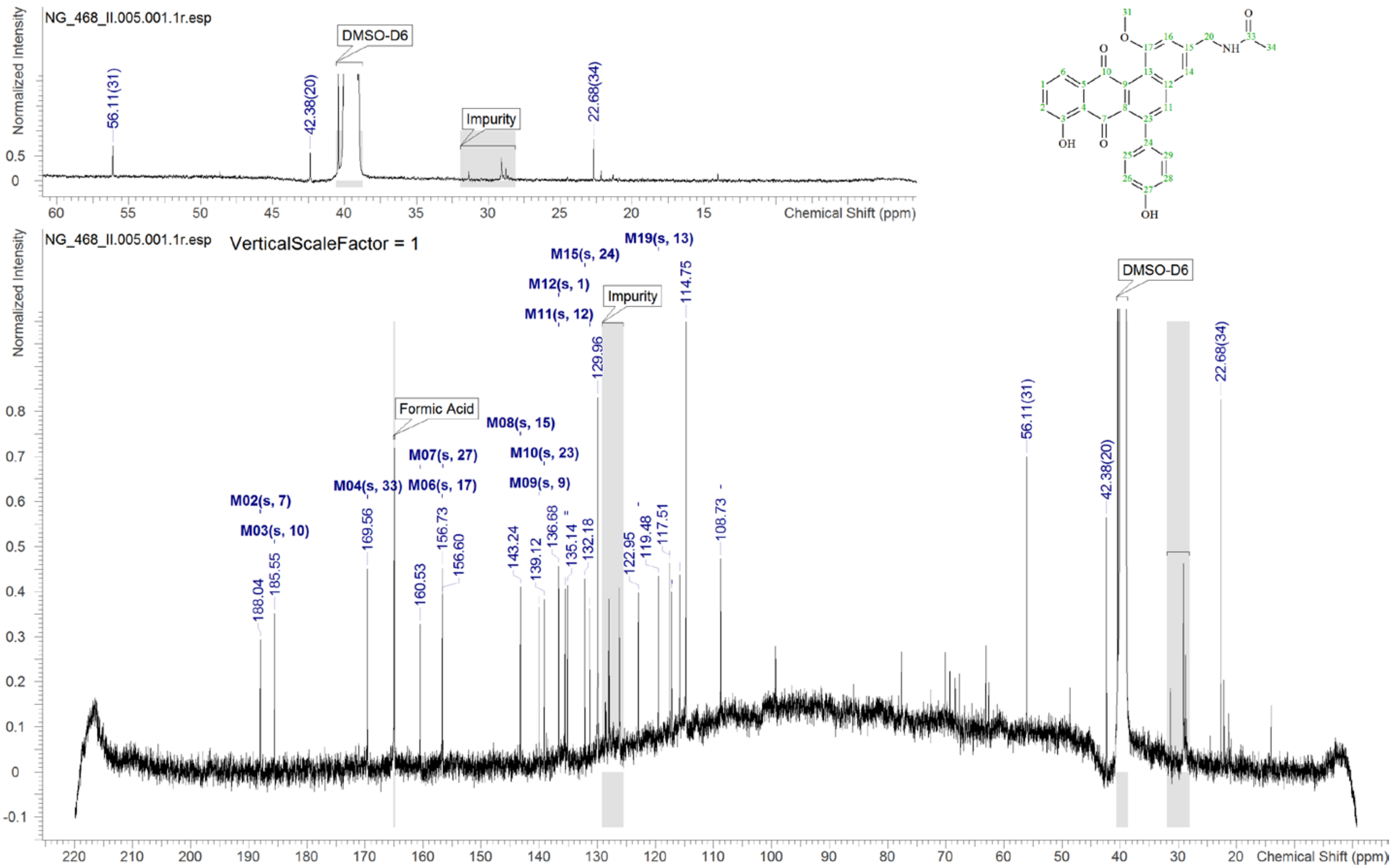

Figure S 13 ^{13}C-NMR (125 MHz, DMSO- d_6) of Pentangumycin; complete spectrum and zoom from 60 to 0 ppm.

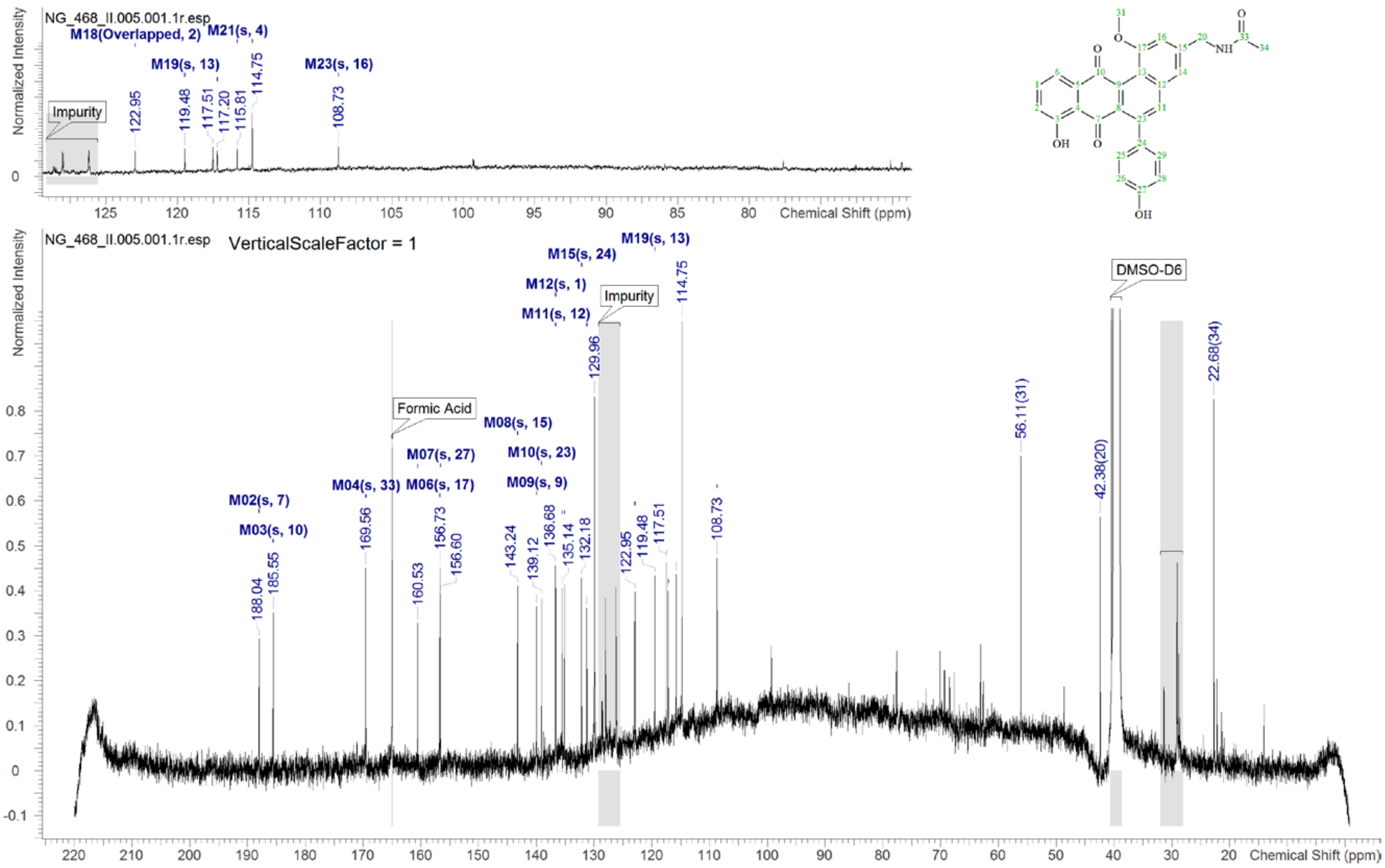

Figure S 14 ^{13}C-NMR (125 MHz, DMSO- d_6) of Pentangumycin; complete spectrum and zoom from 130 to 70 ppm.

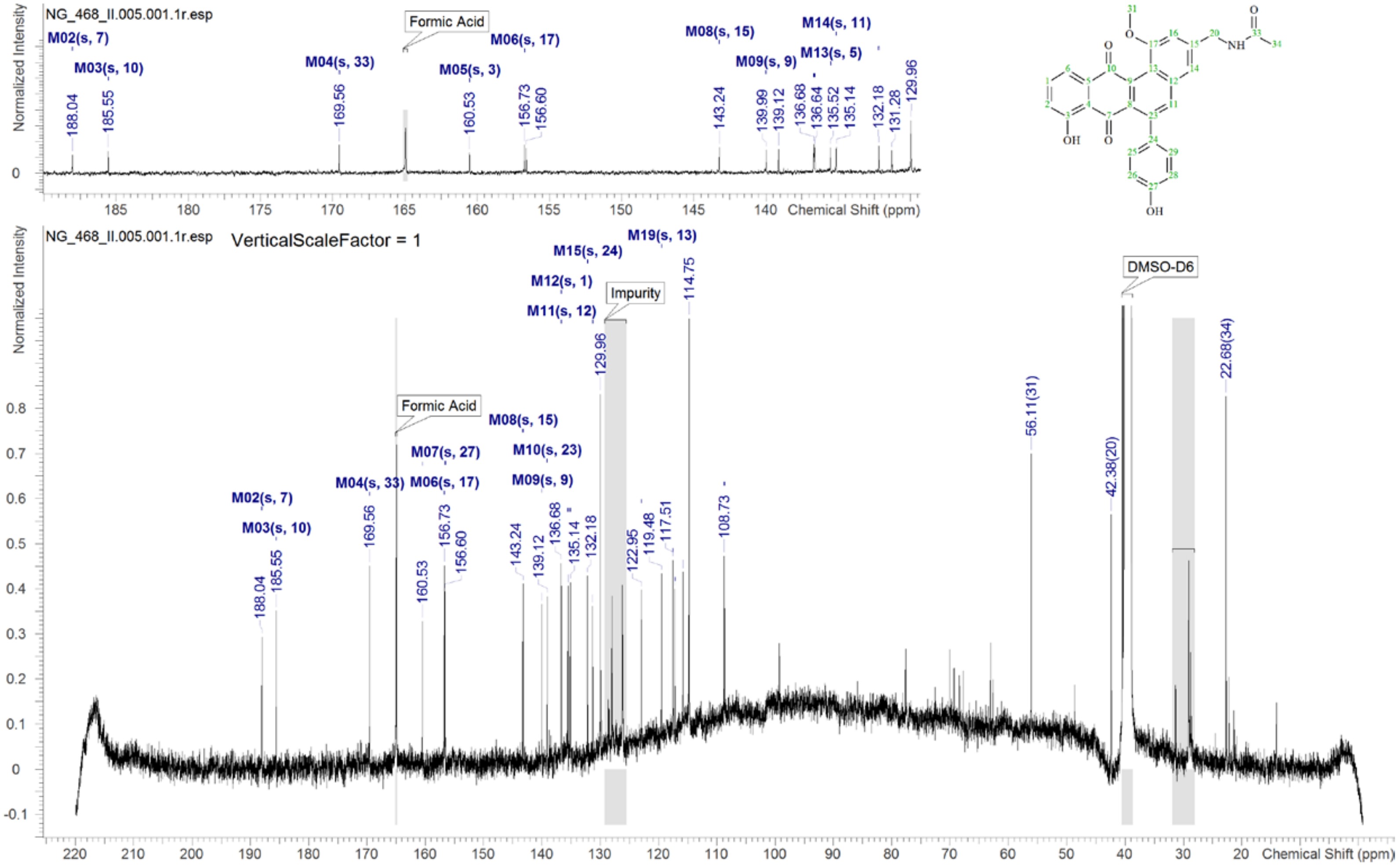

Figure S 15 ^{13}C-NMR (125 MHz, DMSO- d_6) of Pentangumycin; complete spectrum and zoom from 190 to 130 ppm.

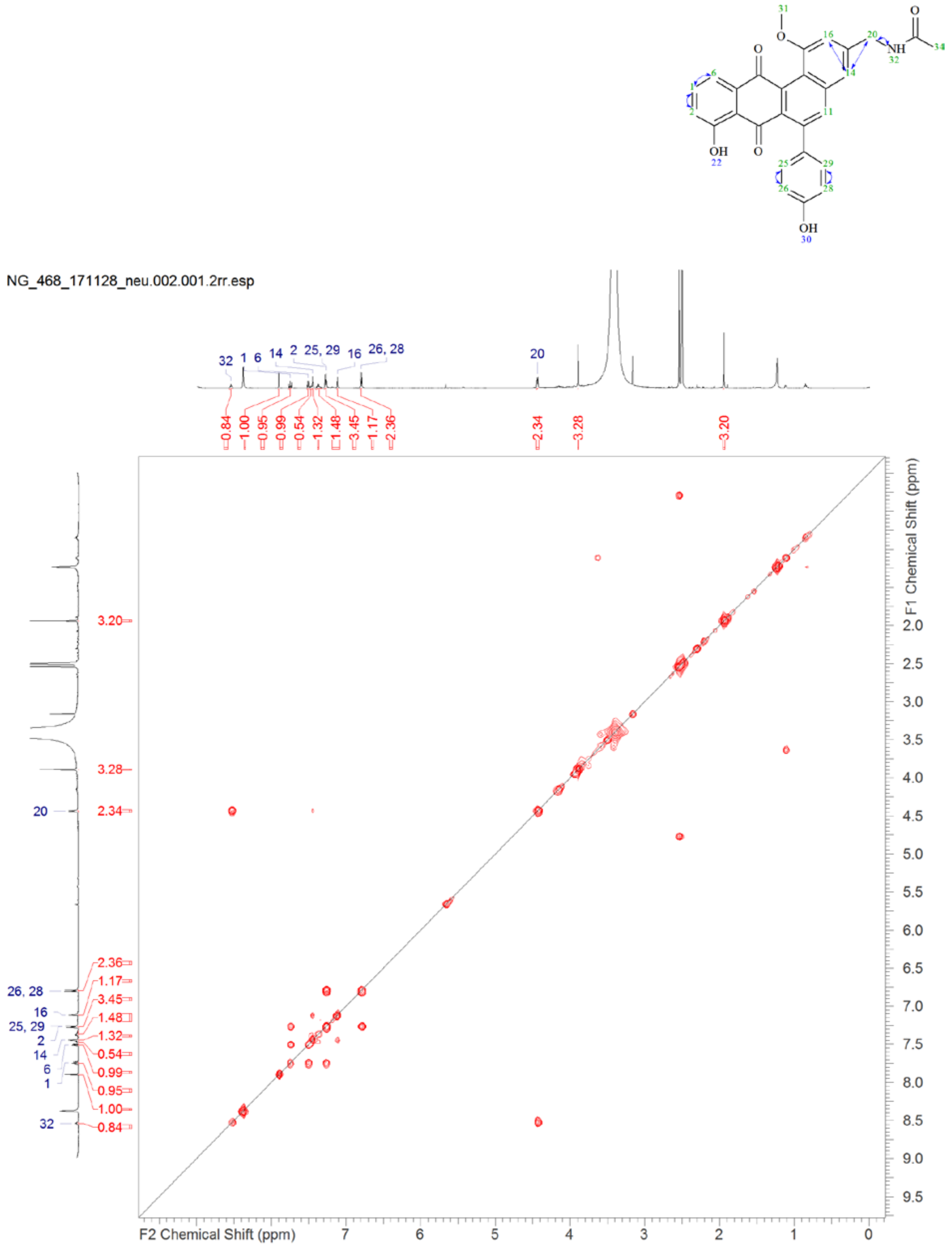

Figure S 16 ^{1}H-^{1}H- COSY spectrum (500 MHz, DMSO-d_6) of Pentangumycin

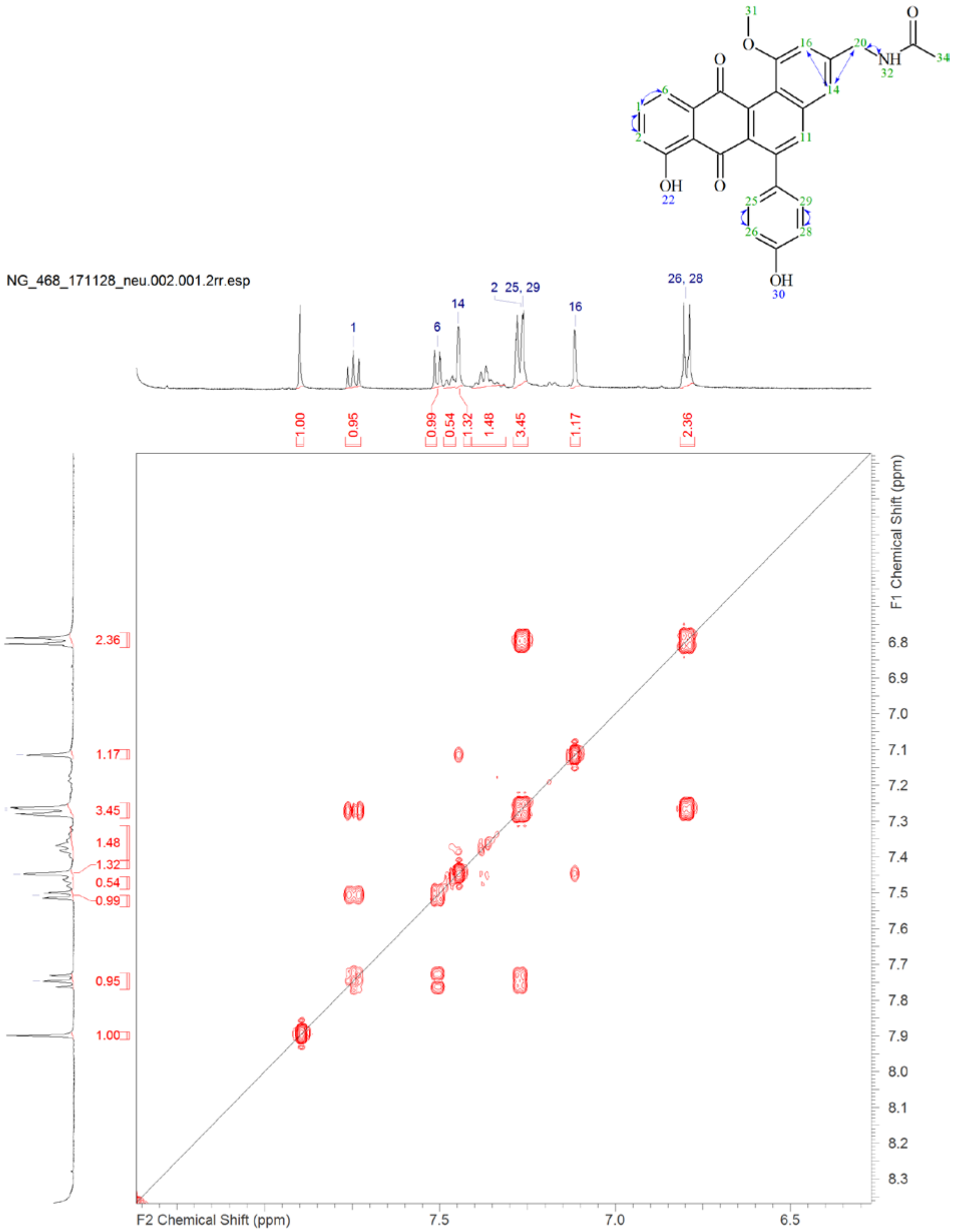

Figure S 17 ¹H-¹H- COSY spectrum (500 MHz, DMSO-d₆) of Pentangumycin; Zoom to aromatic region

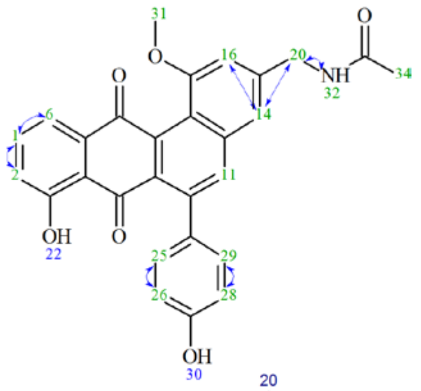

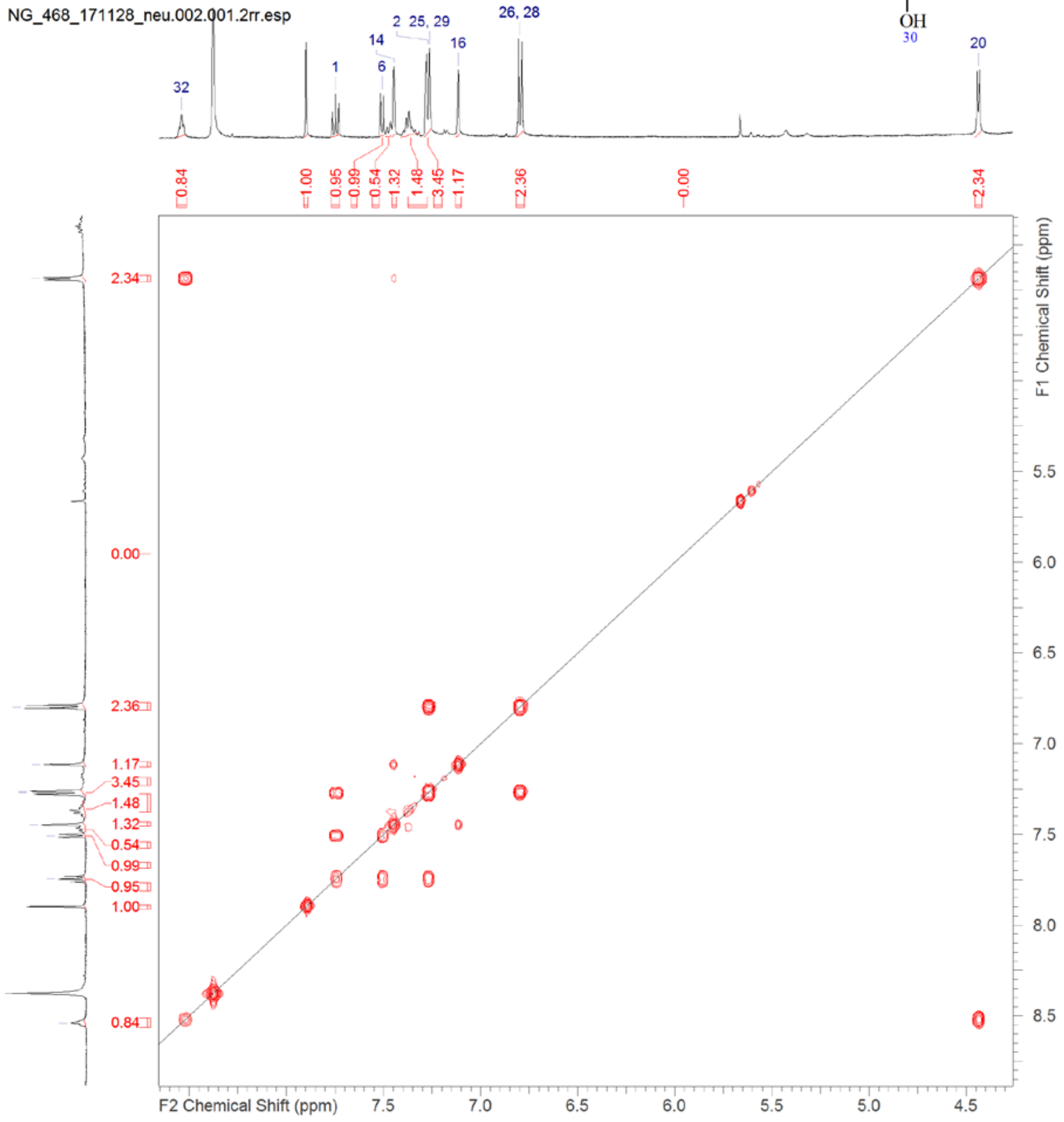

Figure S 18 ^{1}H-^{1}H- COSY spectrum (500 MHz, DMSO-d_6) of Pentangumycin; Zoom from 8.5 to 4.5 ppm

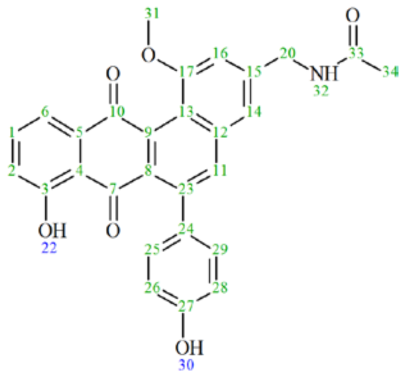

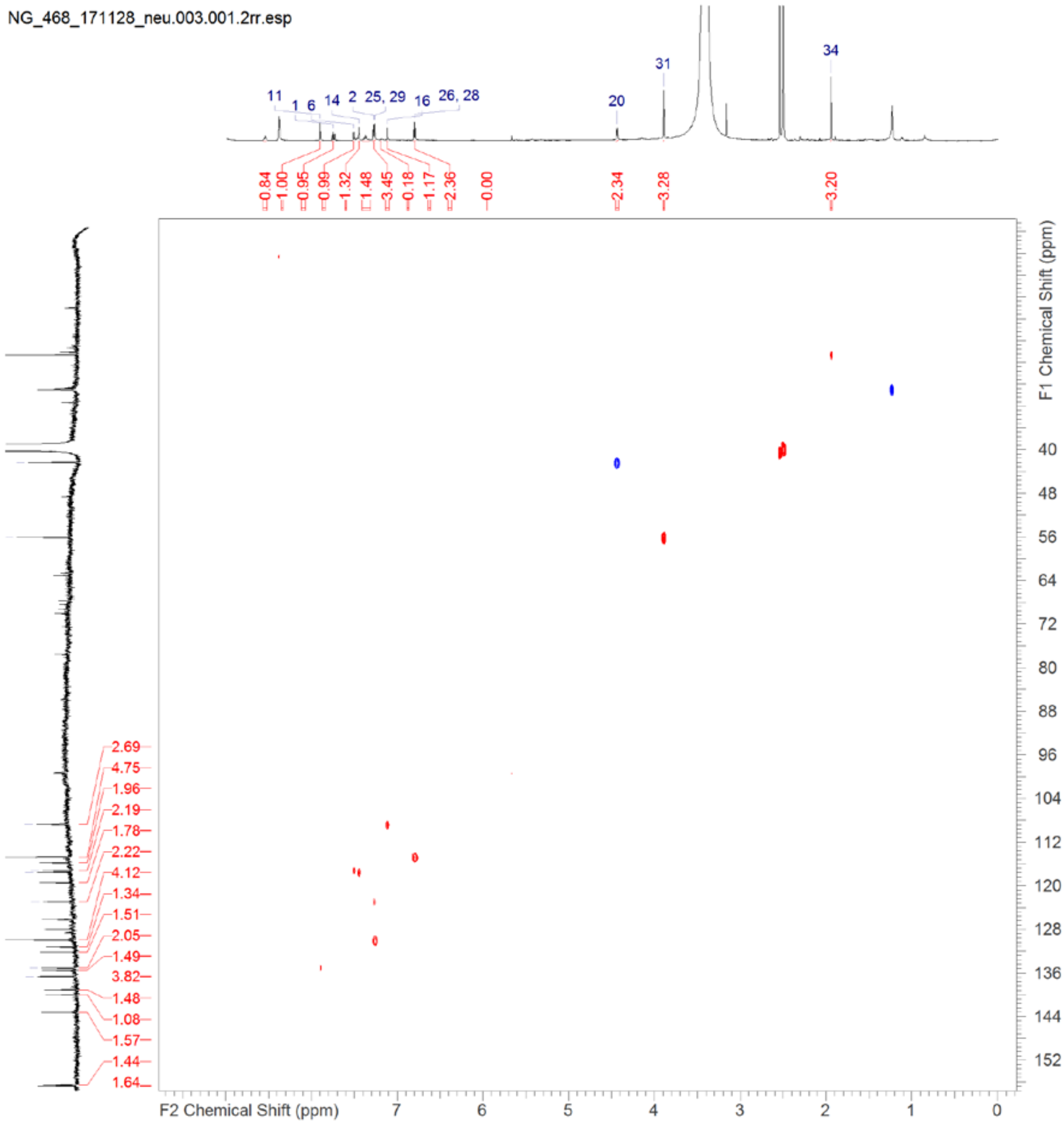

Figure S 19 HSQC-spectrum (500 MHz; 125 MHz, DMSO-d$_6$) of Pentangumycin; complete spectrum

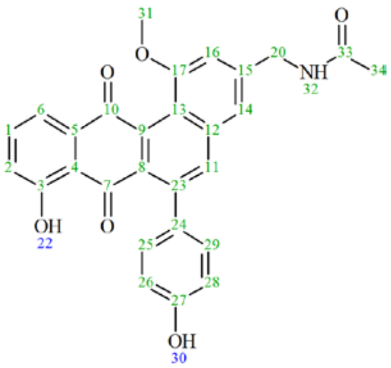

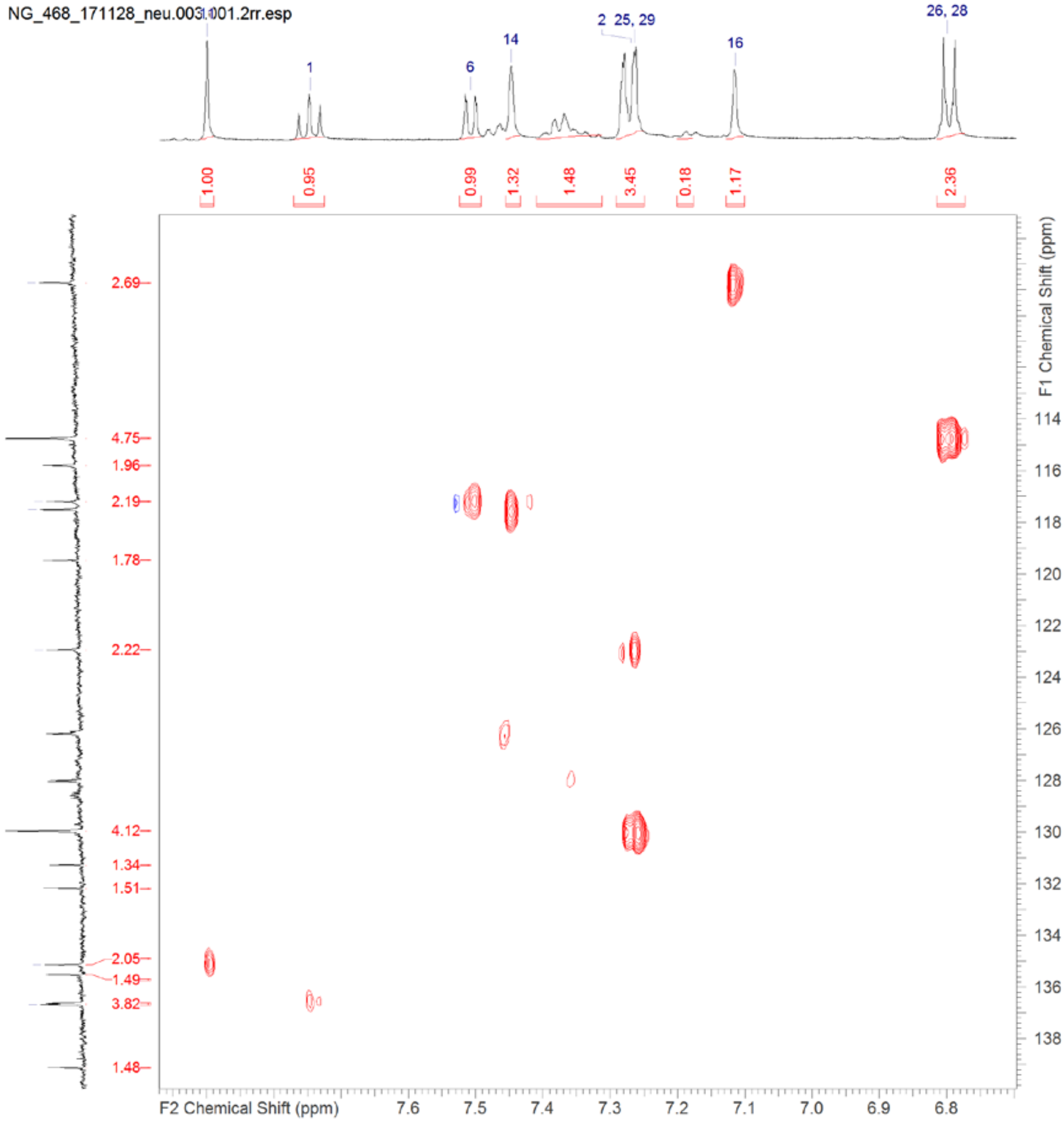

Figure S 20 HSQC-spectrum (500 MHz; 125 MHz, DMSO-d₆) of Pentangumycin; zoom from 7.9 to 6.8 ppm and 139 to 107 ppm

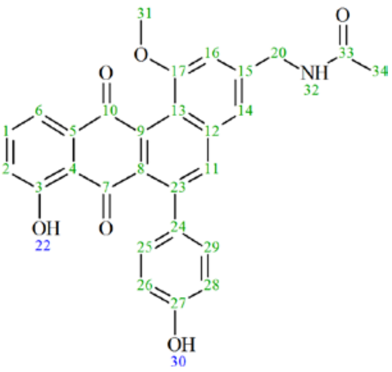

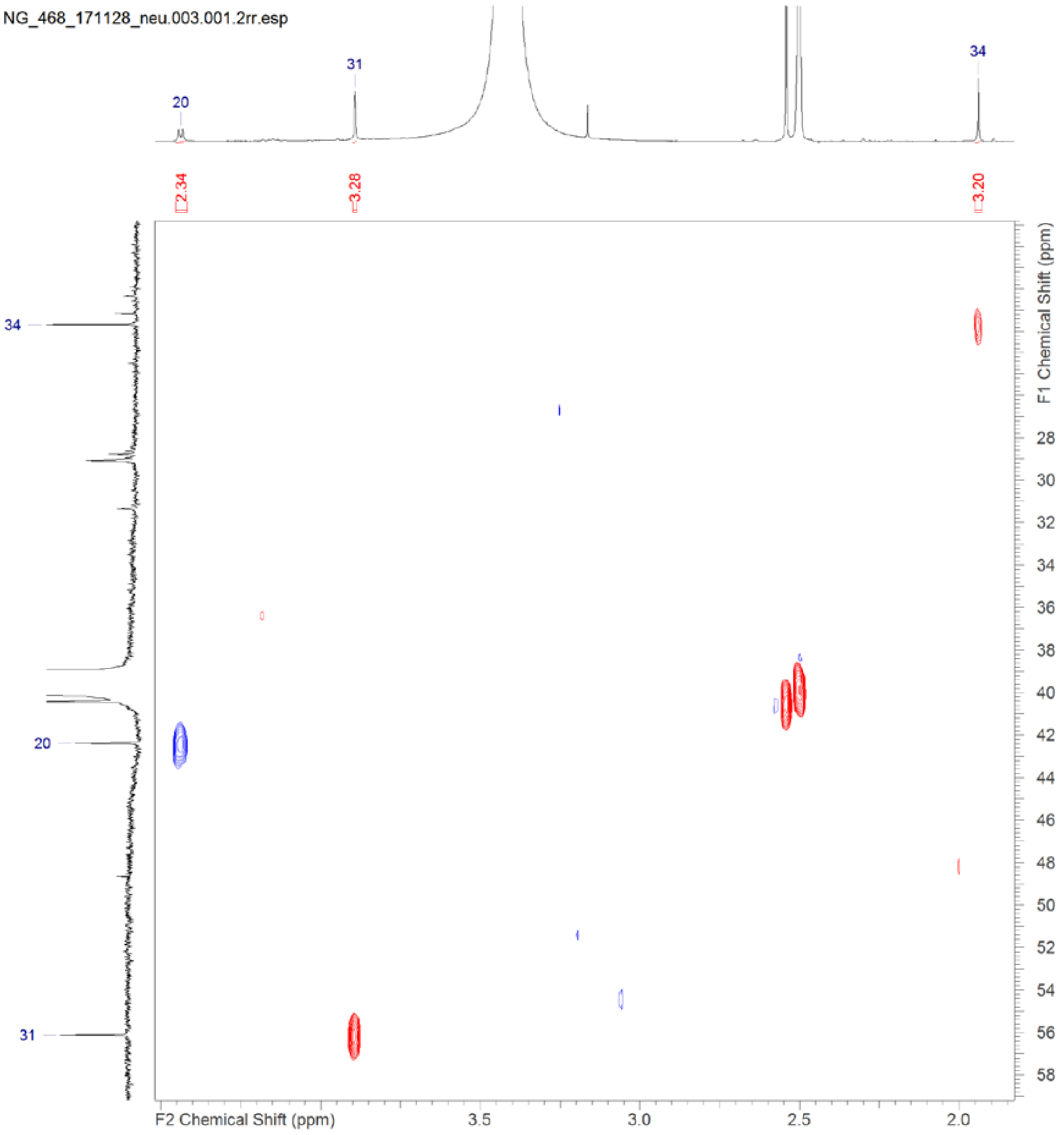

Figure S 21 HSQC-spectrum (500 MHz; 125 MHz, DMSO-d_6) of Pentangumycin; zoom from 4 to 2 ppm and 59 to 19 ppm

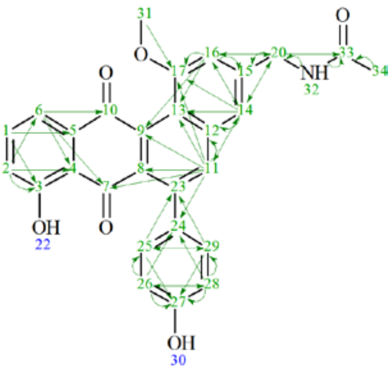

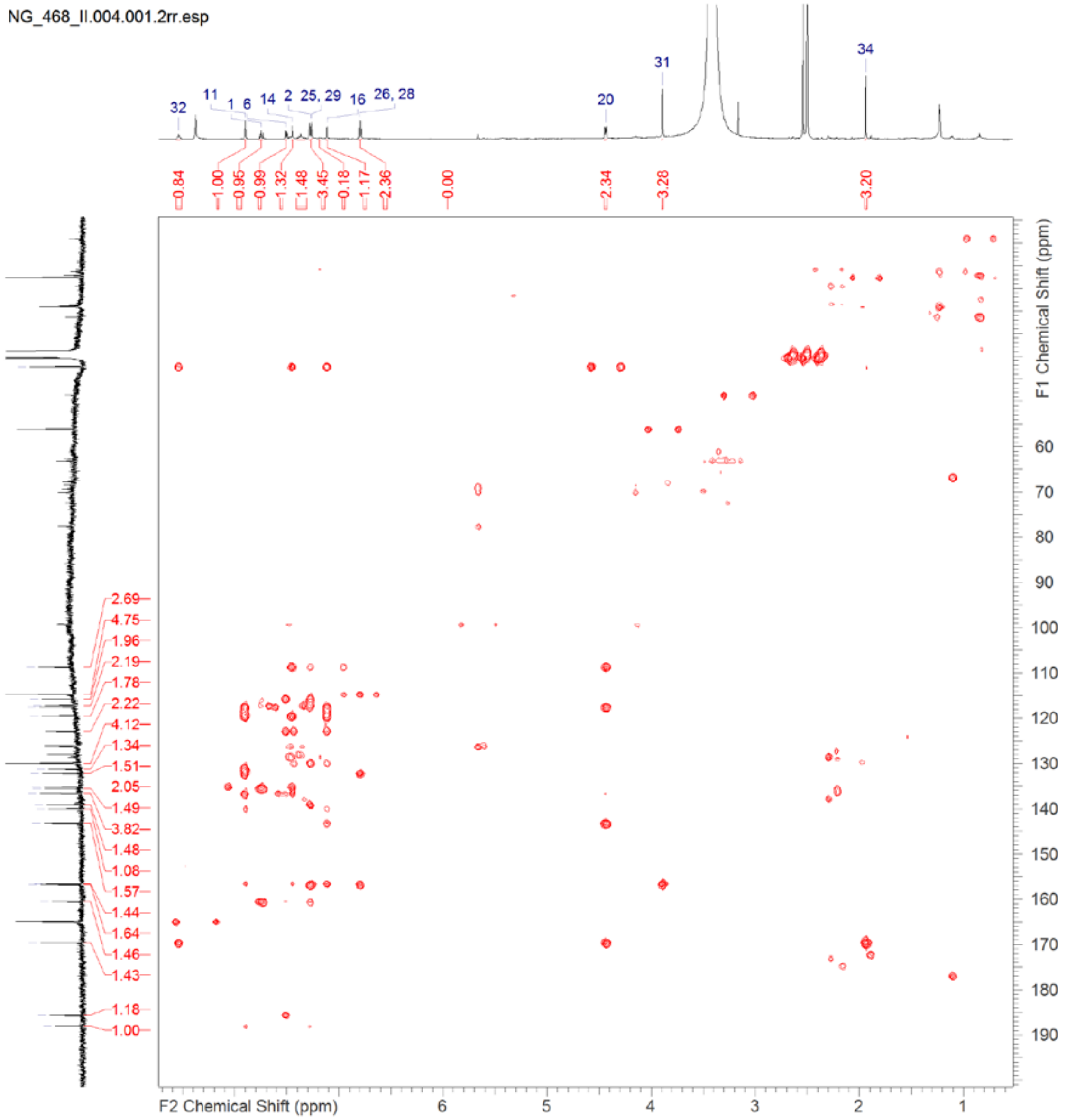

Figure S 22 HMBC-spectrum (500 MHz; 125 MHz, DMSO-d6) of Pentangumycin; complete spectrum

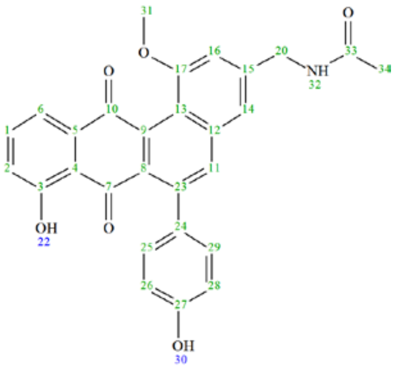

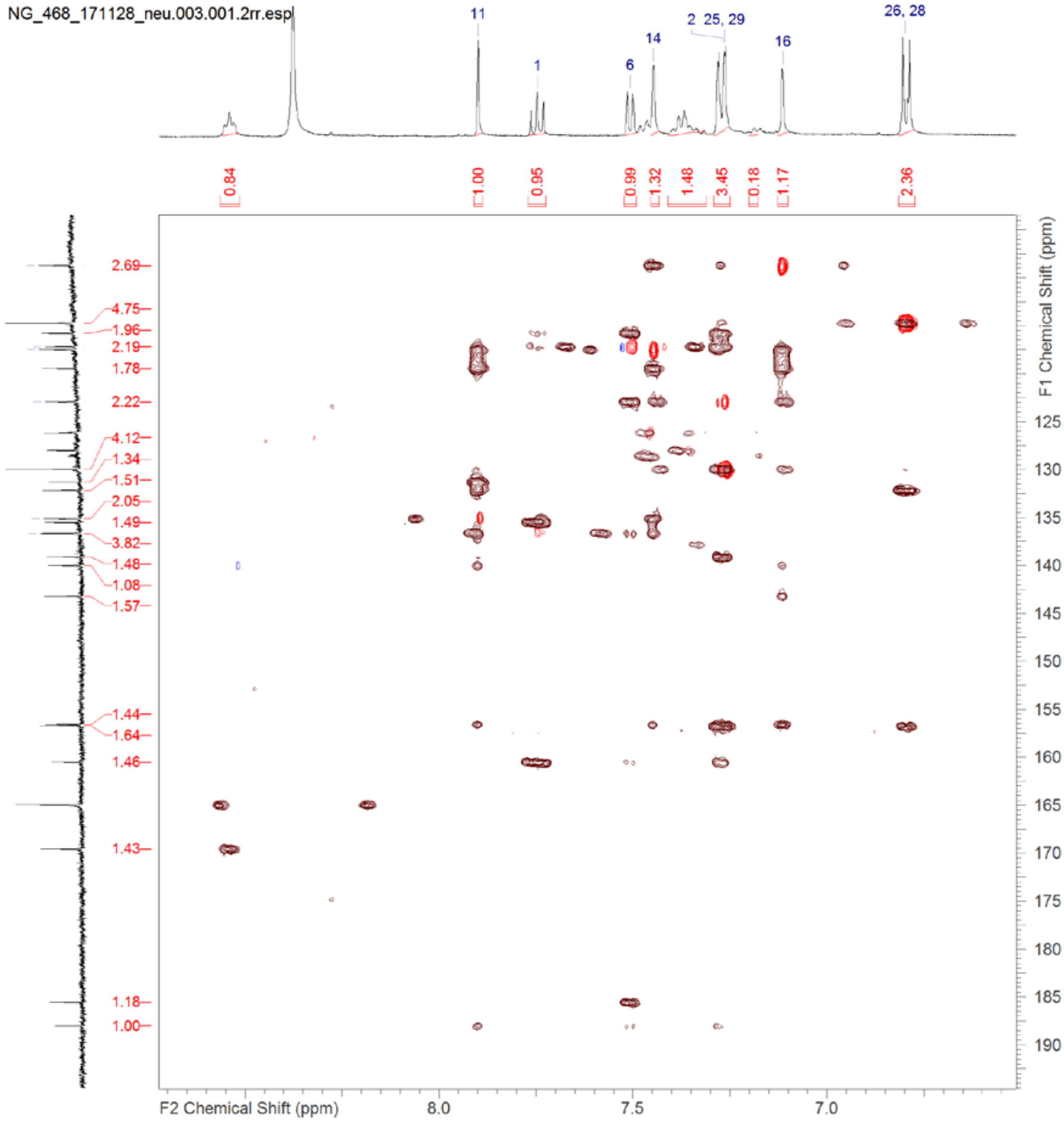

Figure S 23 HMBC - HSQC-Overlay spectrum (500 MHz; 125 MHz, DMSO-d$_6$) of Pentangumycin (black: HMBC; red/blue: HSQC); zoom from 9.2 to 6.5 ppm and 195 to 110 ppm

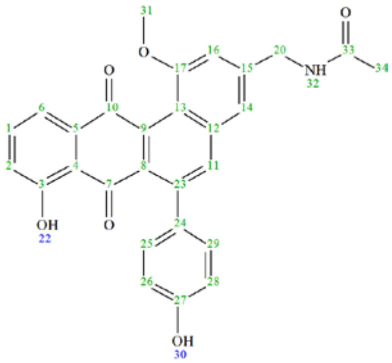

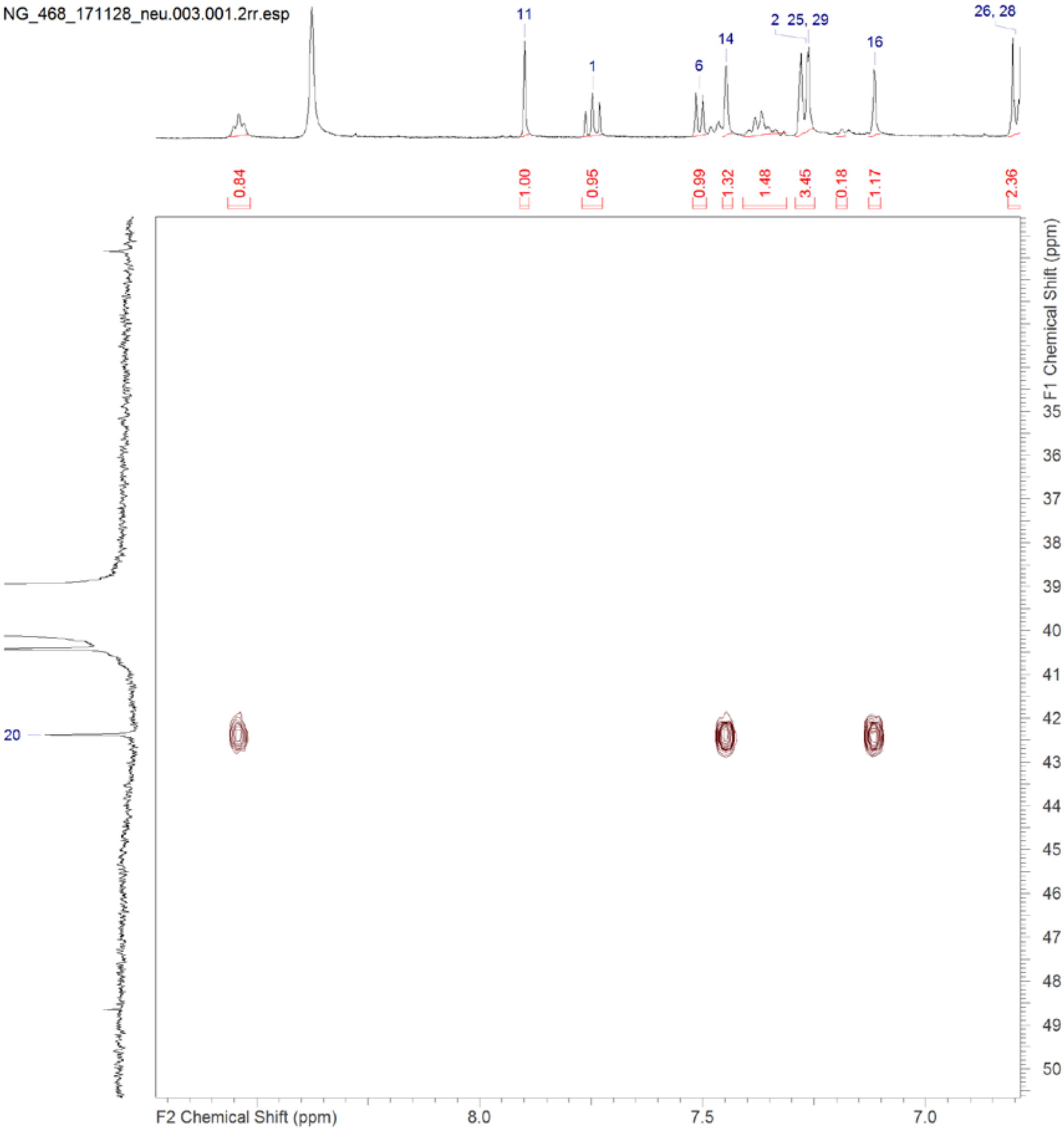

Figure S 24 HMBC - HSQC-Overlay spectrum (500 MHz; 125 MHz, DMSO-d$_6$) of Pentangumycin (black: HMBC; red/blue: HSQC); zoom from 9.2 to 6.5 ppm and 50 to 31 ppm

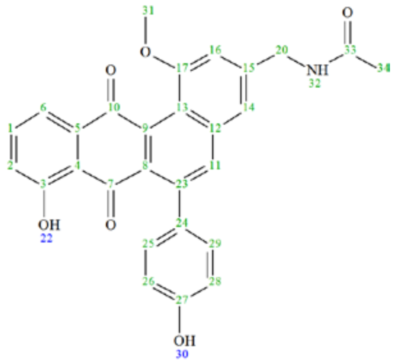

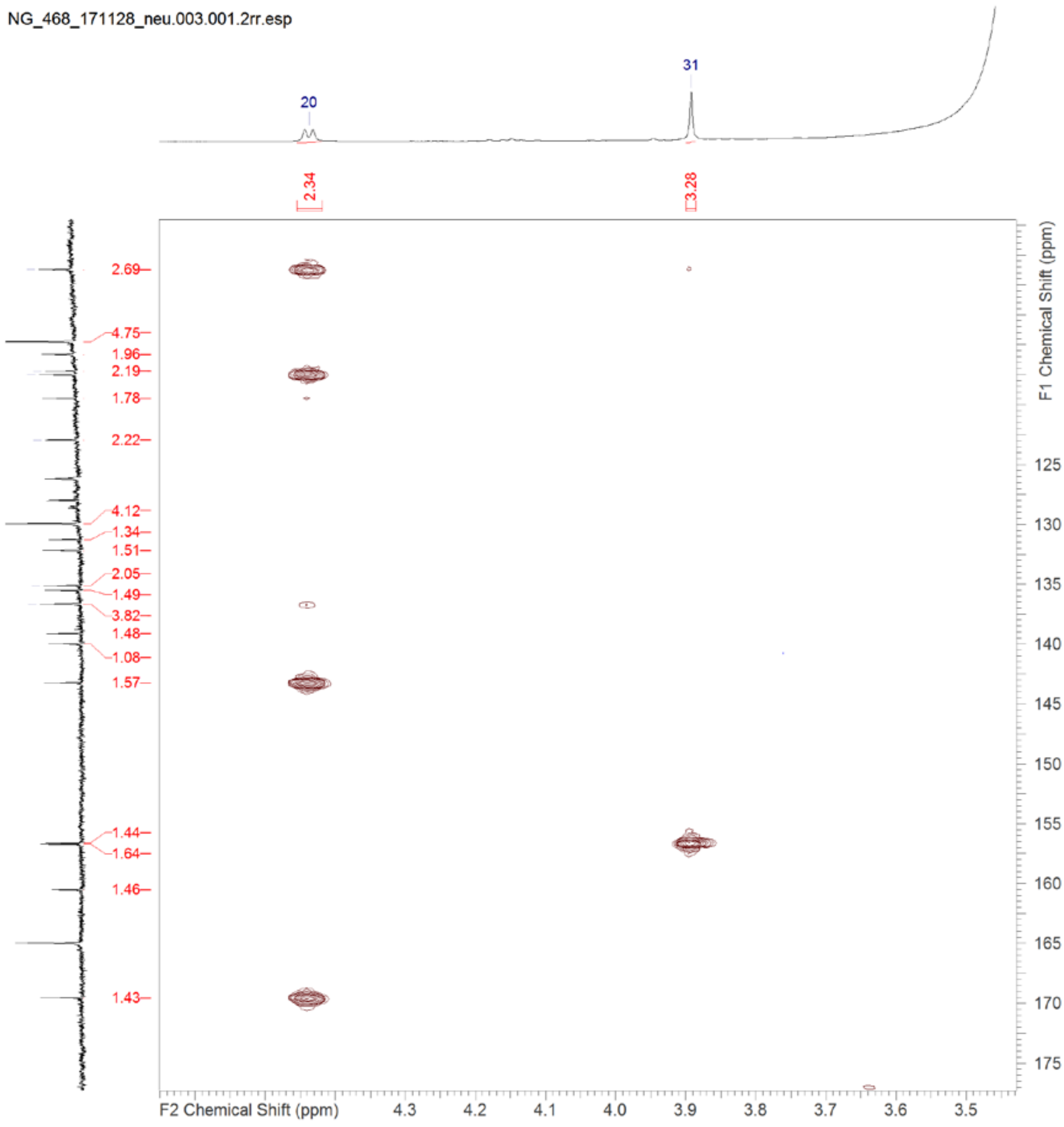

Figure S 25 HMBC - HSQC-Overlay spectrum (500 MHz; 125 MHz, DMSO-d₆) of Pentangumycin (black: HMBC; red/blue: HSQC); zoom from 4.6 to 3.5 ppm and 110 to 175 ppm

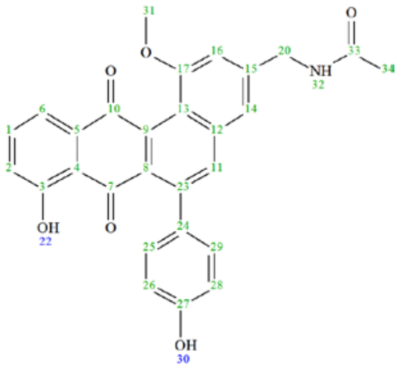

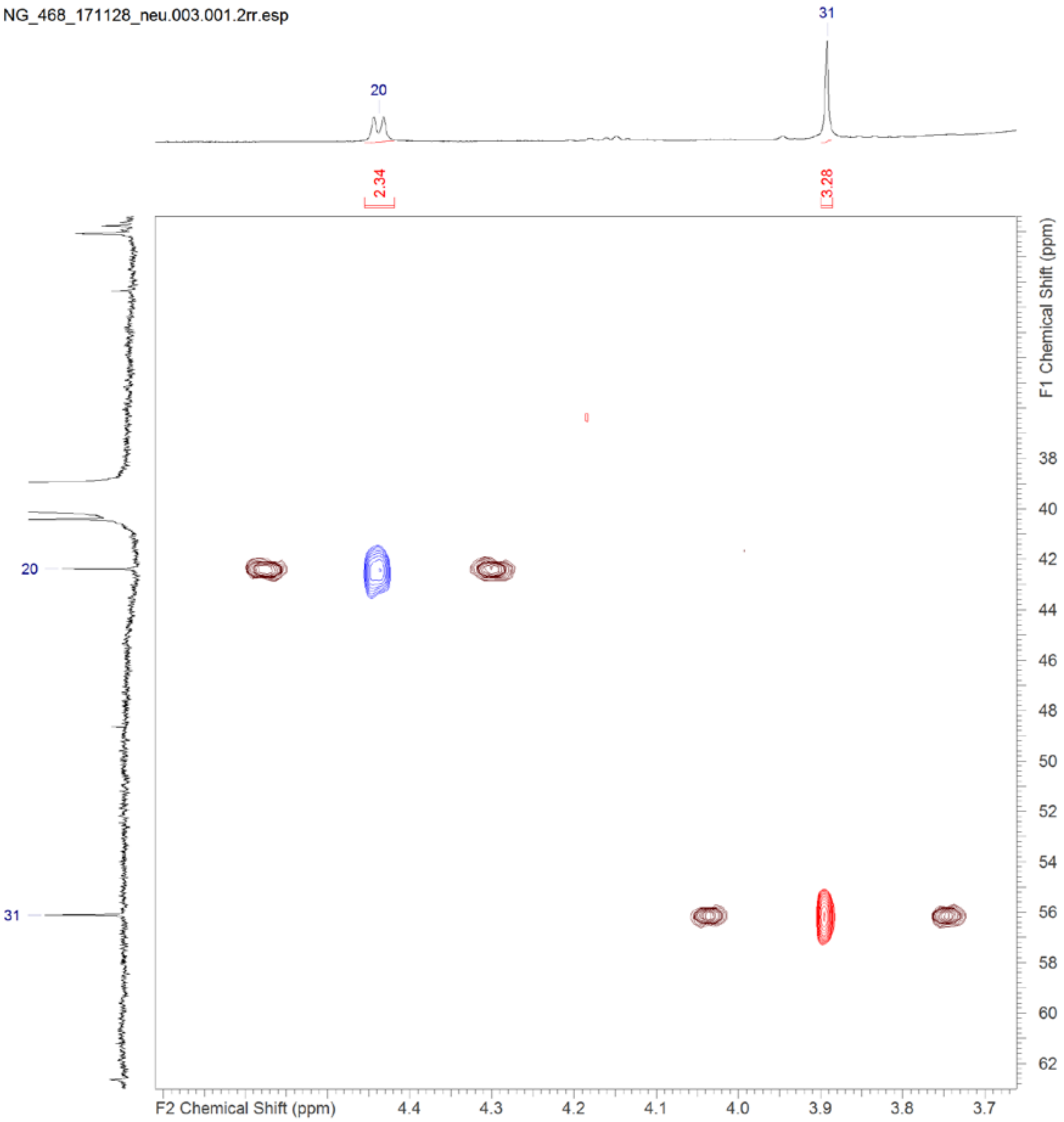

Figure S 26 HMBC - HSQC-Overlay spectrum (500 MHz; 125 MHz, DMSO-d_6) of Pentangumycin (black: HMBC; red/blue: HSQC); zoom from 4.6 to 3.7 ppm and 62 to 34 ppm

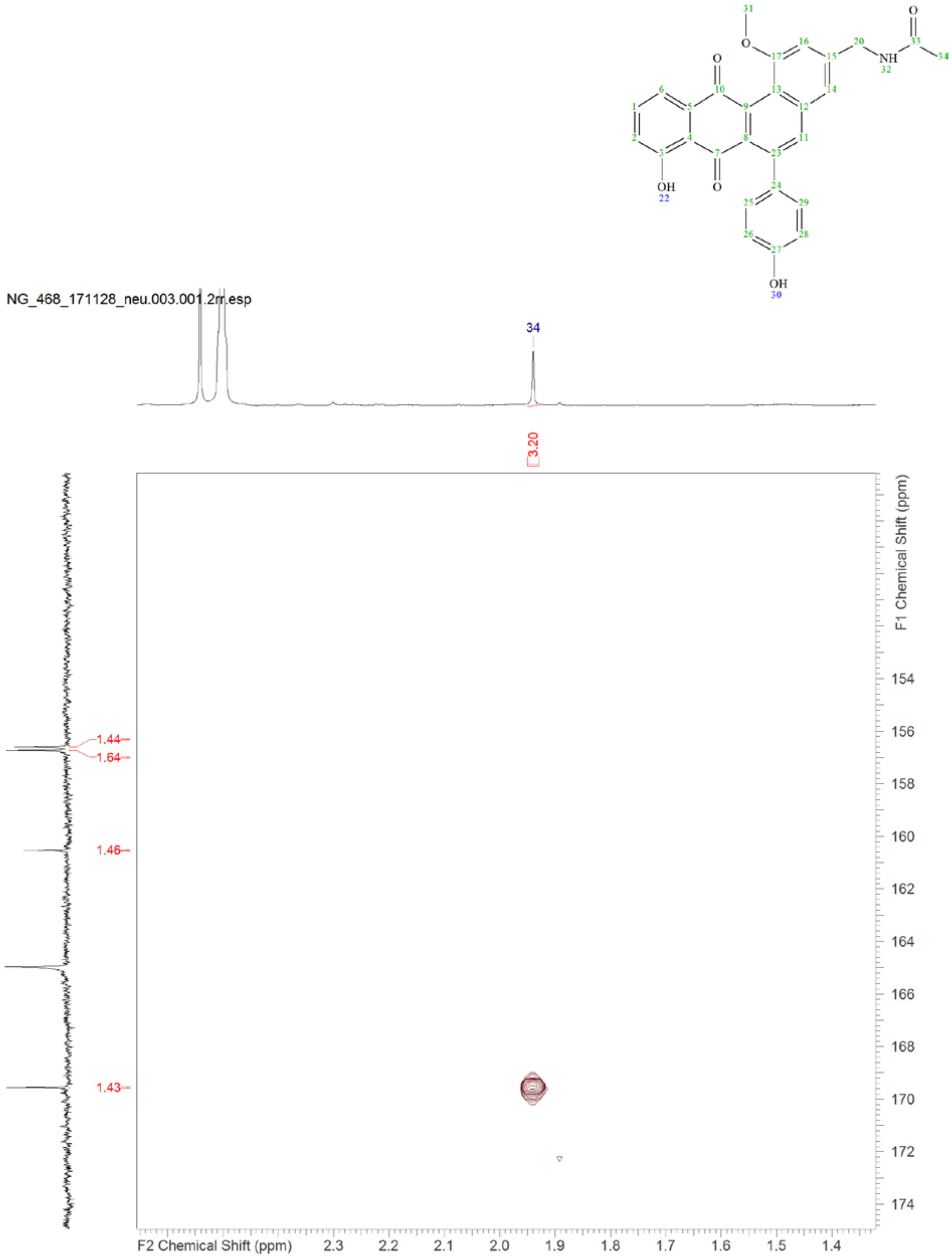

Figure S 27 HMBC - HSQC-Overlay spectrum (500 MHz; 125 MHz, DMSO-d_6) of Pentangumycin (black: HMBC; red/blue: HSQC); zoom from 2.6 to 1.4 ppm and 174 to 151 ppm

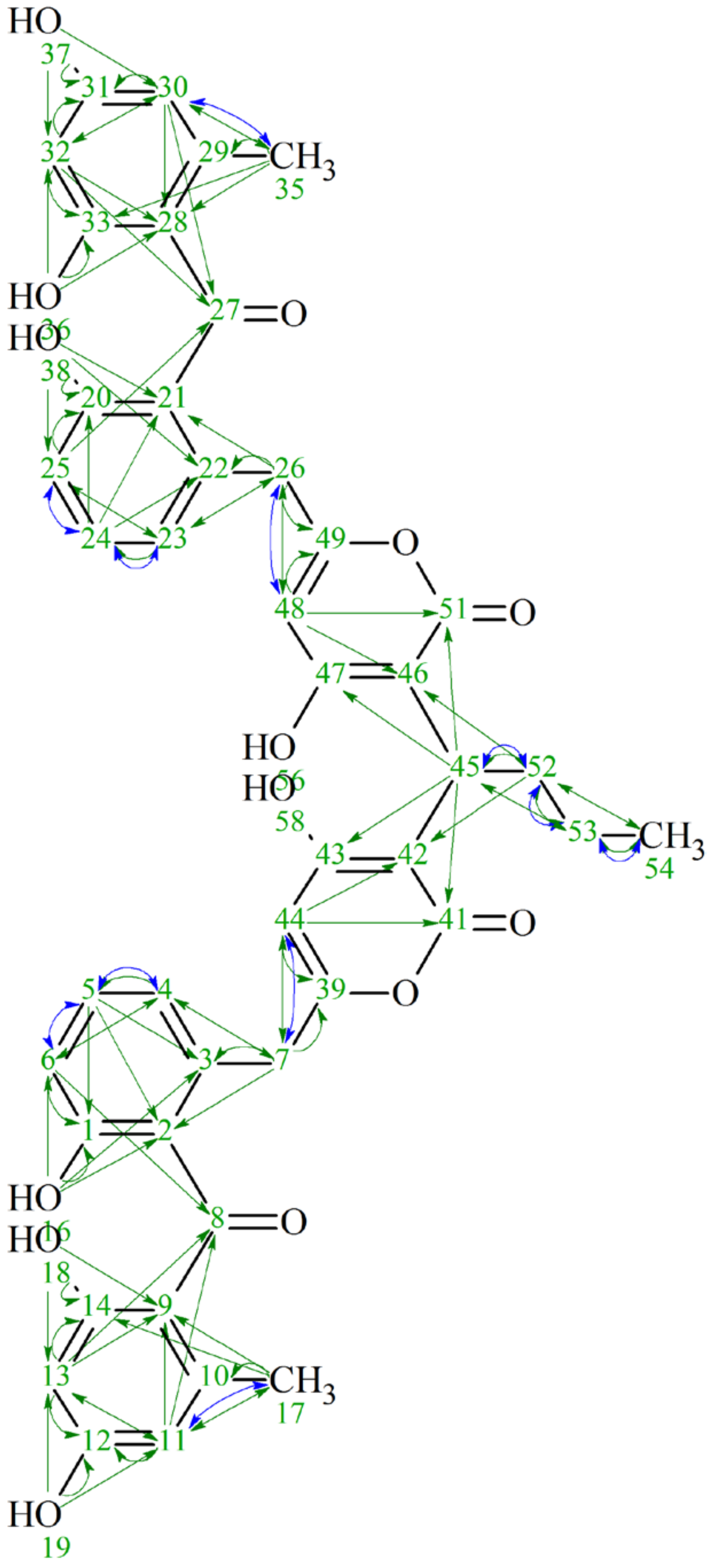

Figure S 28 Structure of SEK90 with all correlations (green: HMBC correlations H → C; blue: ^{1}H-^{1}H-Cosy correlation)

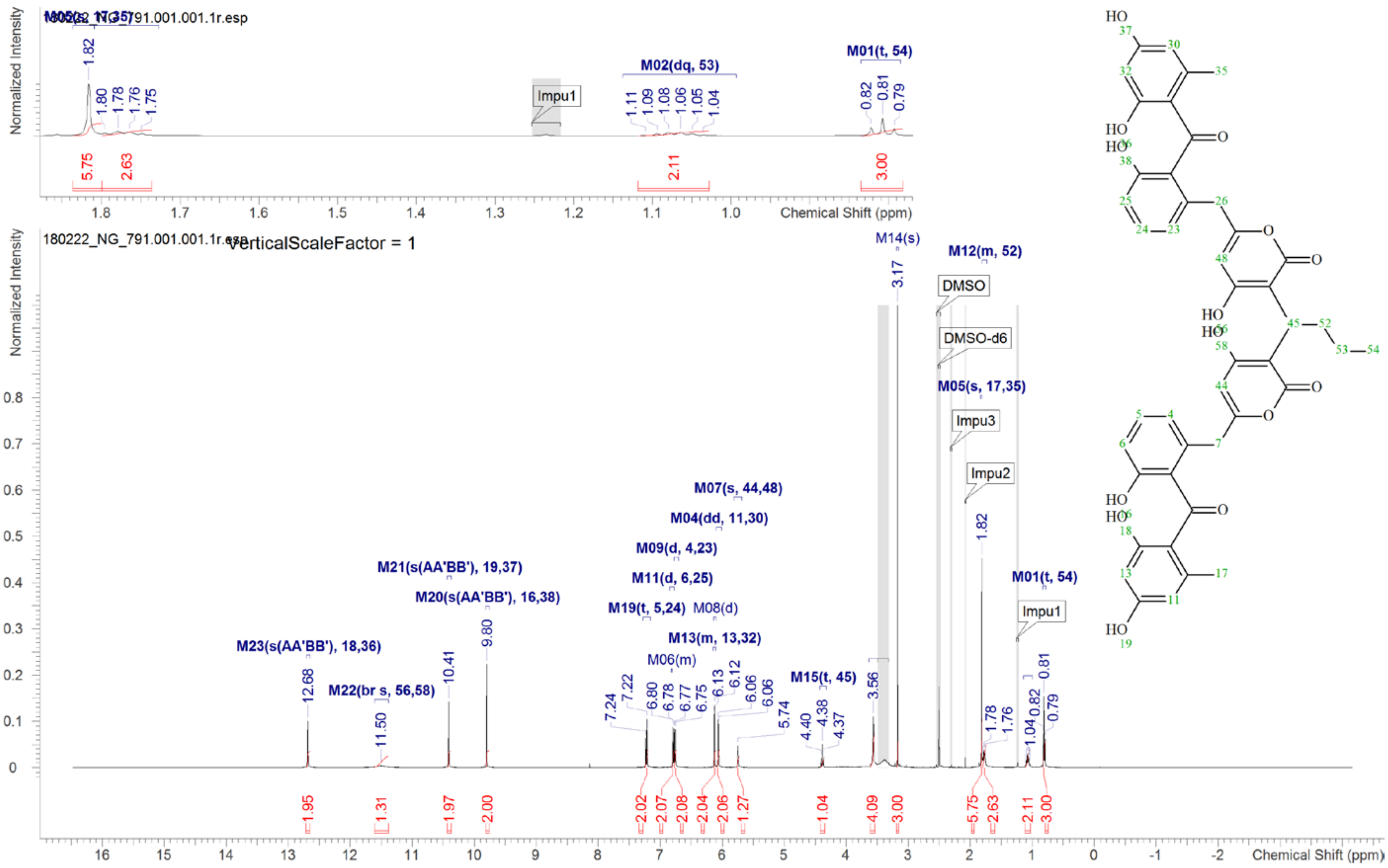

Figure S 29 ^{1}H-NMR spectrum (500 MHz, DMSO-d_6) of SEK90; complete Spectrum and zoom from 1.8 to 0.7 ppm

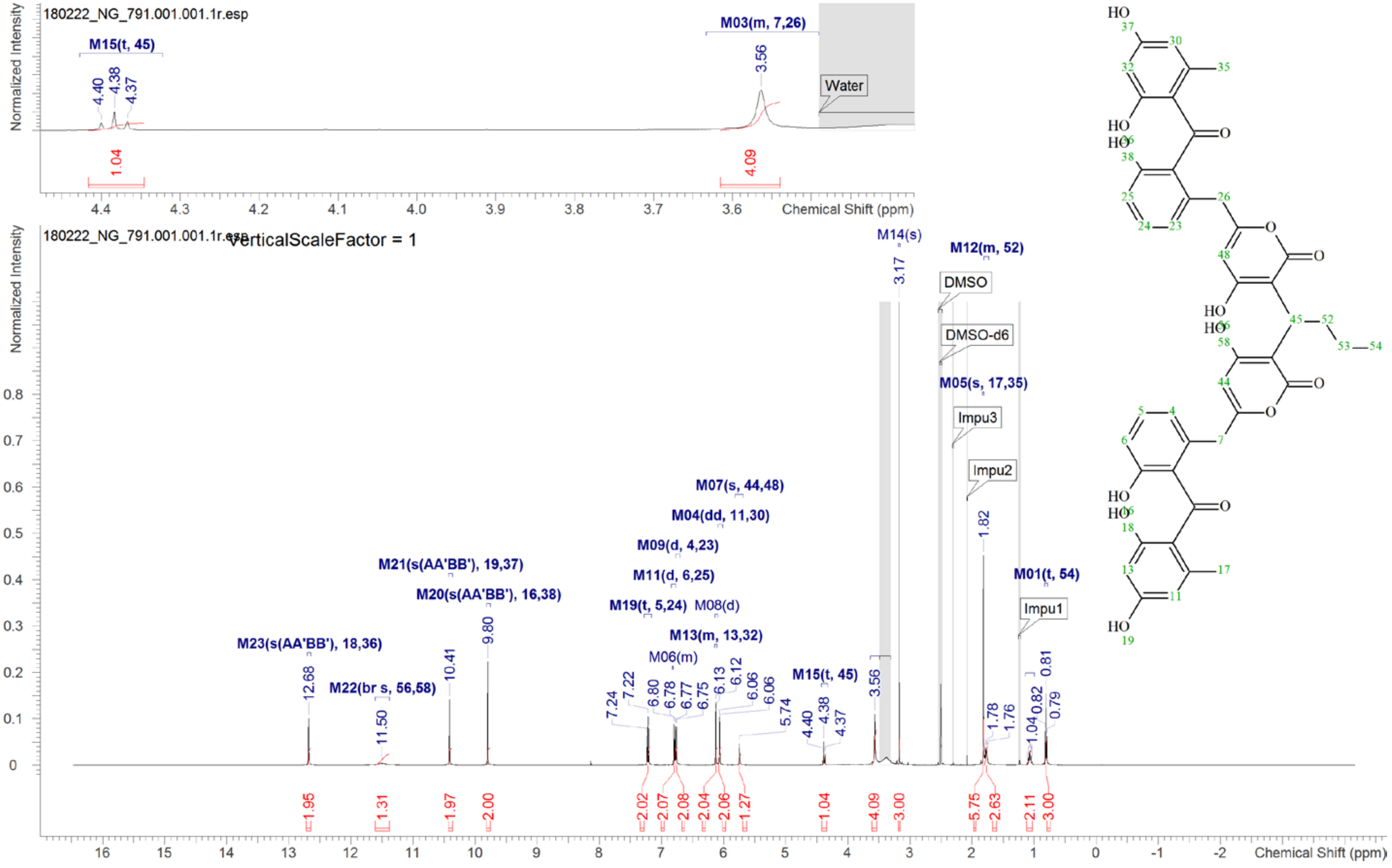

Figure S 30 ¹H-NMR spectrum (500 MHz, DMSO-d₆) of SEK90; complete Spectrum and zoom from 4.4 to 3.4 ppm

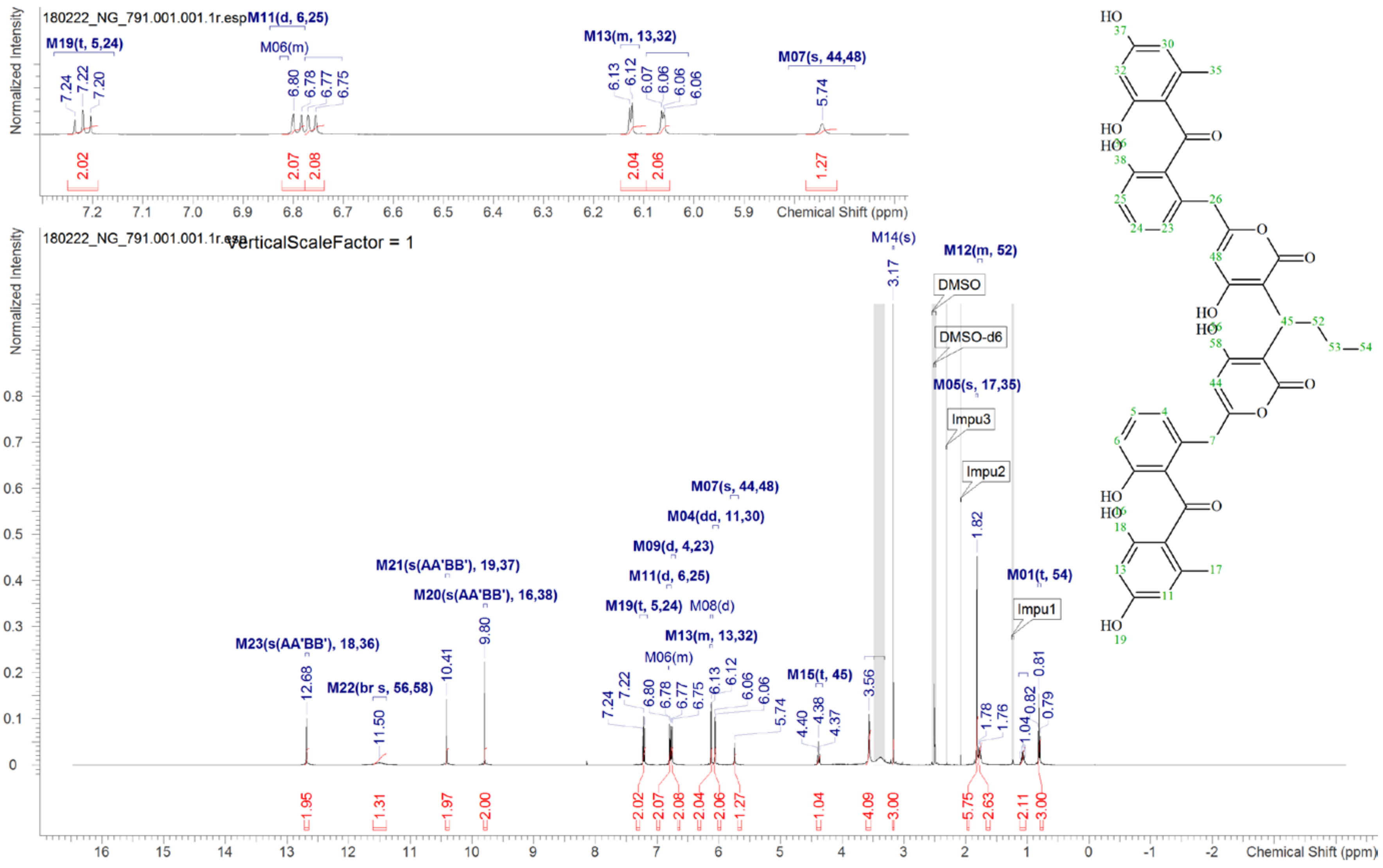

Figure S 31 ^{1}H-NMR spectrum (500 MHz, DMSO-d_6) of SEK90; complete Spectrum and zoom from 7.2 to 5.7 ppm

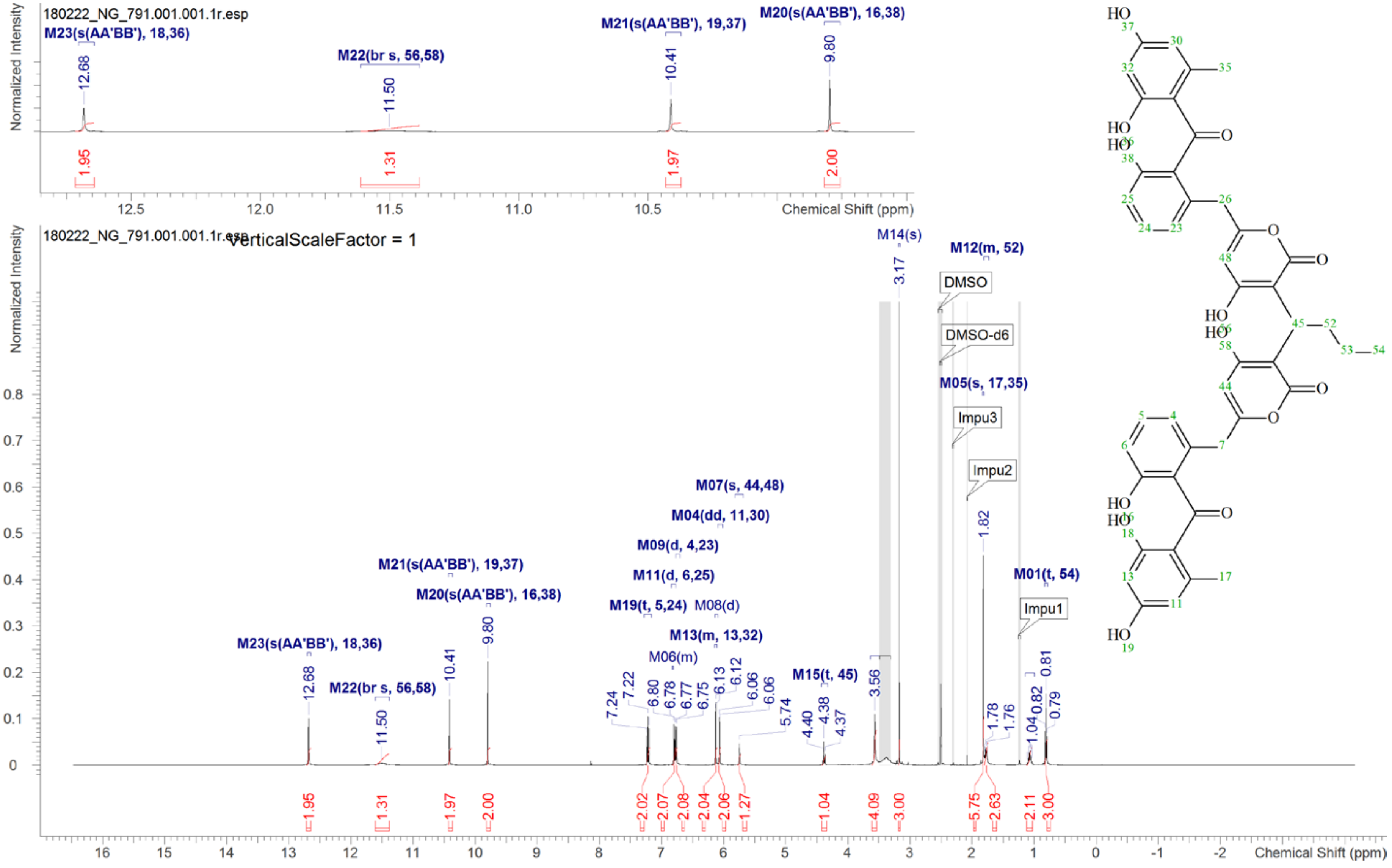

Figure S 32 ^{1}H-NMR spectrum (500 MHz, DMSO-d_6) of SEK90; complete Spectrum and zoom from 13 to 9.5 ppm

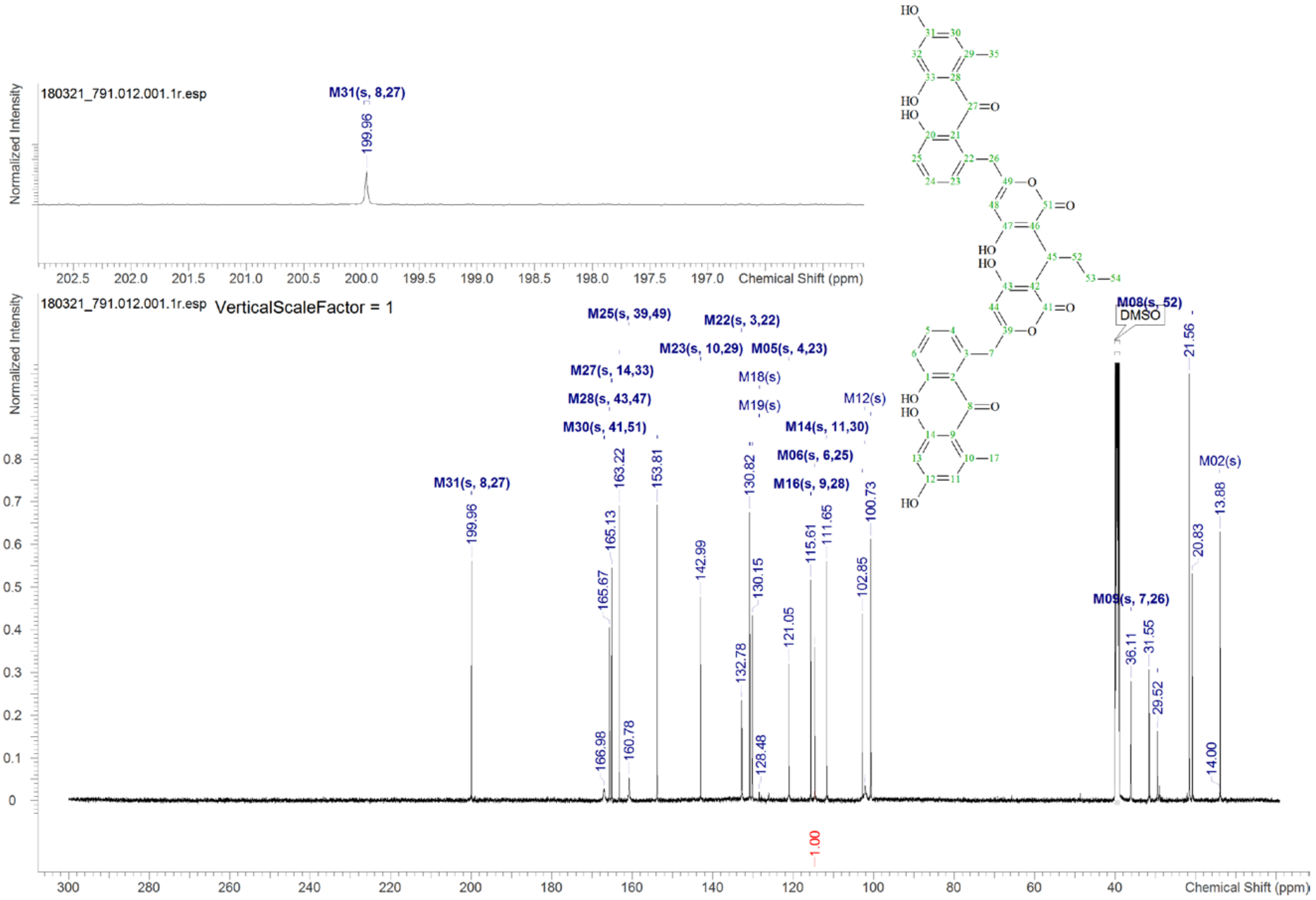

Figure S 33 ^{13}C-NMR spectrum (125 MHz, DMSO-d$_6$) of SEK90; complete Spectrum and zoom from 202 to 196 ppm

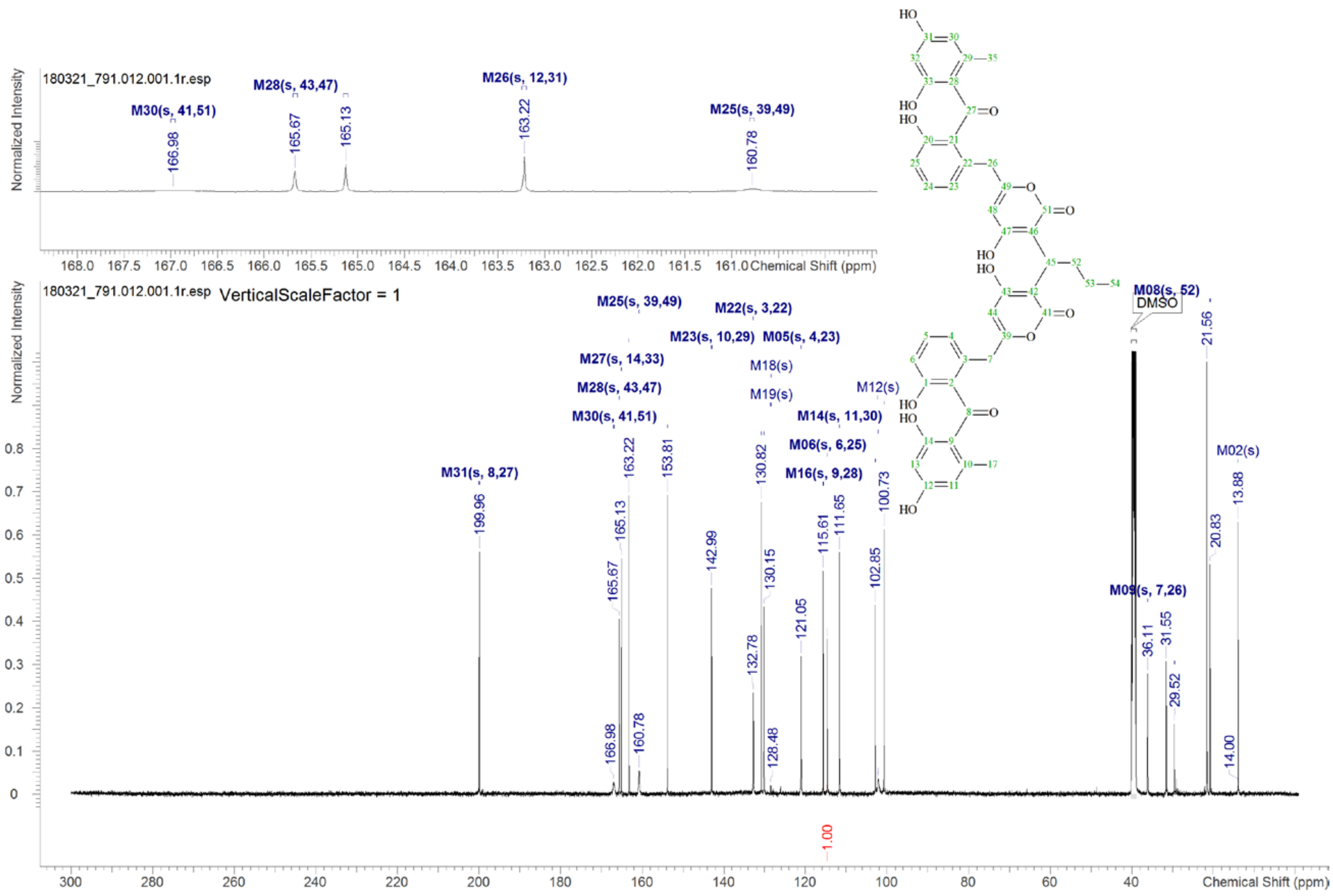

Figure S 34 ^{13}C-NMR spectrum (125 MHz, DMSO-d$_6$) of SEK90; complete Spectrum and zoom from 168 to 60 ppm

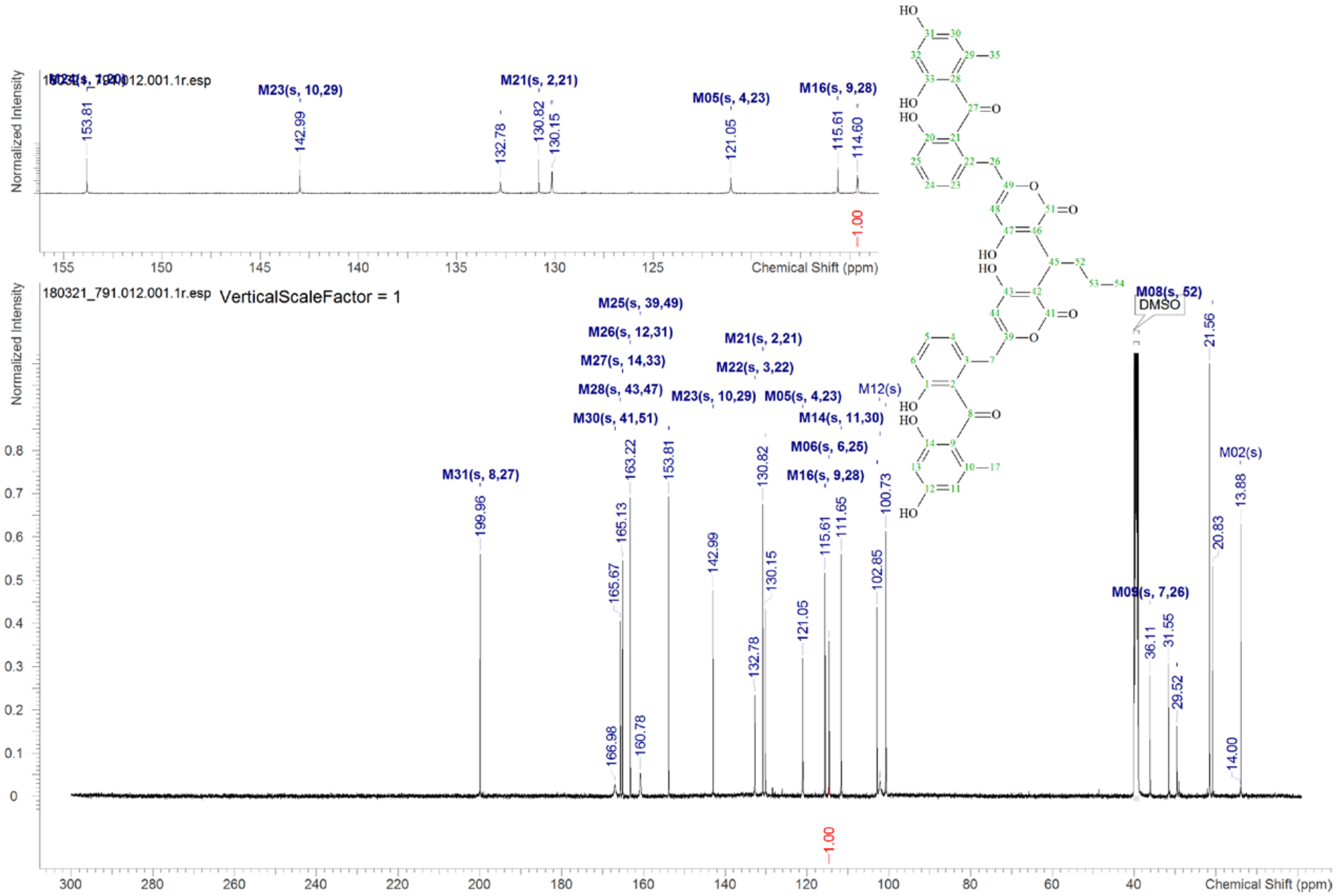

Figure S 35 13C-NMR spectrum (125 MHz, DMSO-d6) of SEK90; complete Spectrum and zoom from 155 to 113 ppm

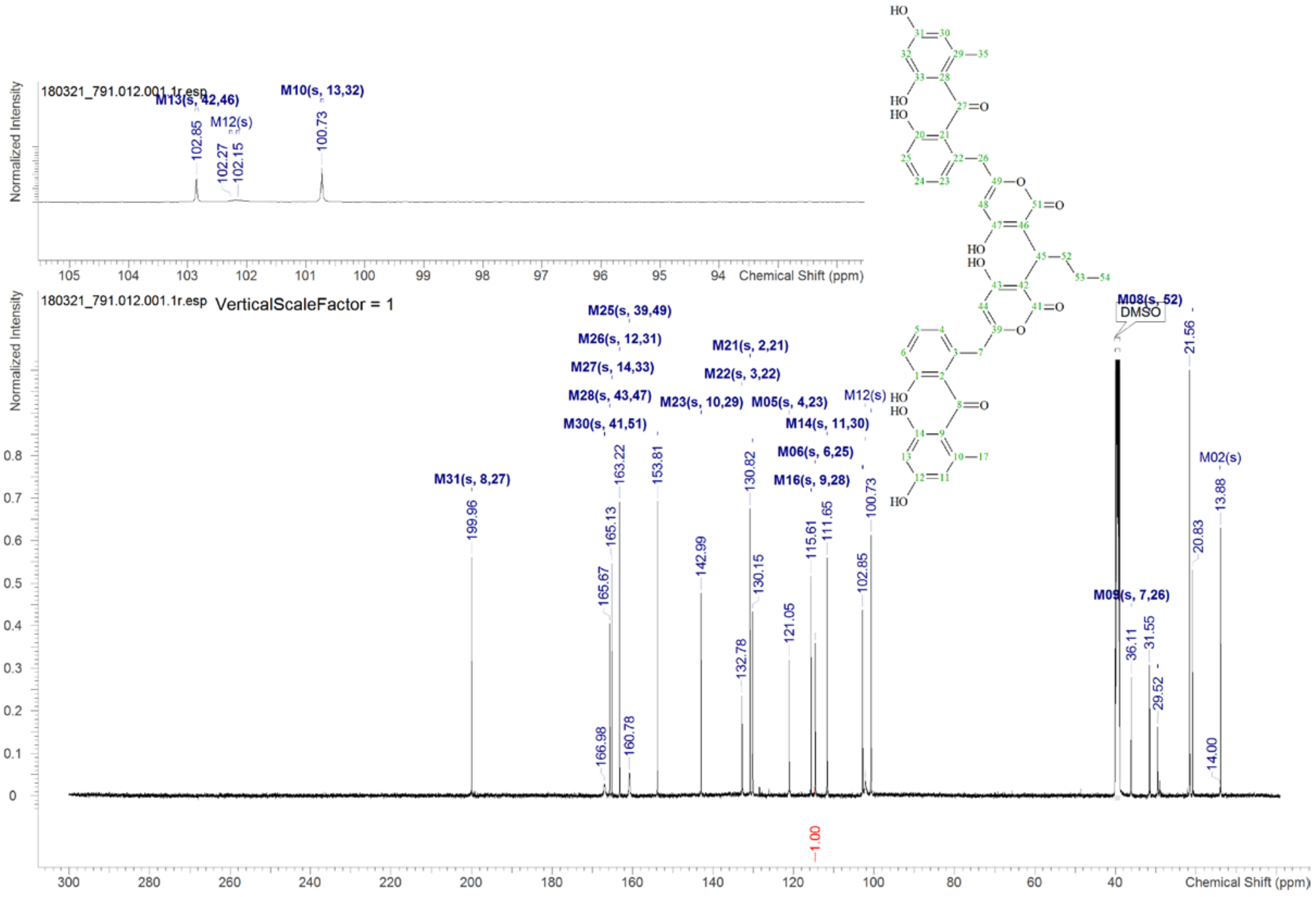

Figure S 36 ^{13}C-NMR spectrum (125 MHz, DMSO-d_6) of SEK90; complete Spectrum and zoom from 105 to 92 ppm

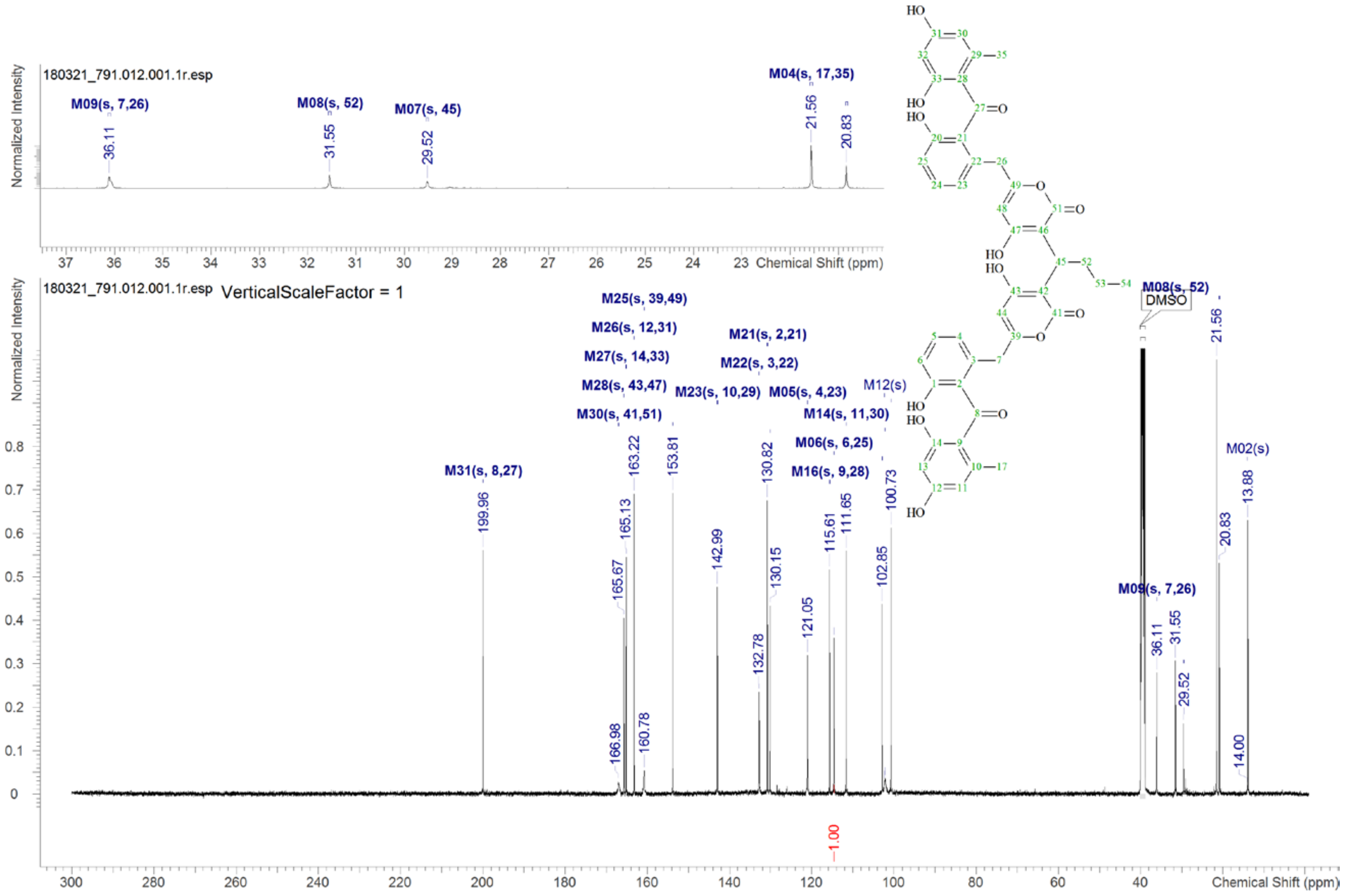

Figure S 37 ^{13}C-NMR spectrum (125 MHz, DMSO-d_6) of SEK90; complete Spectrum and zoom from 37 to 20 ppm

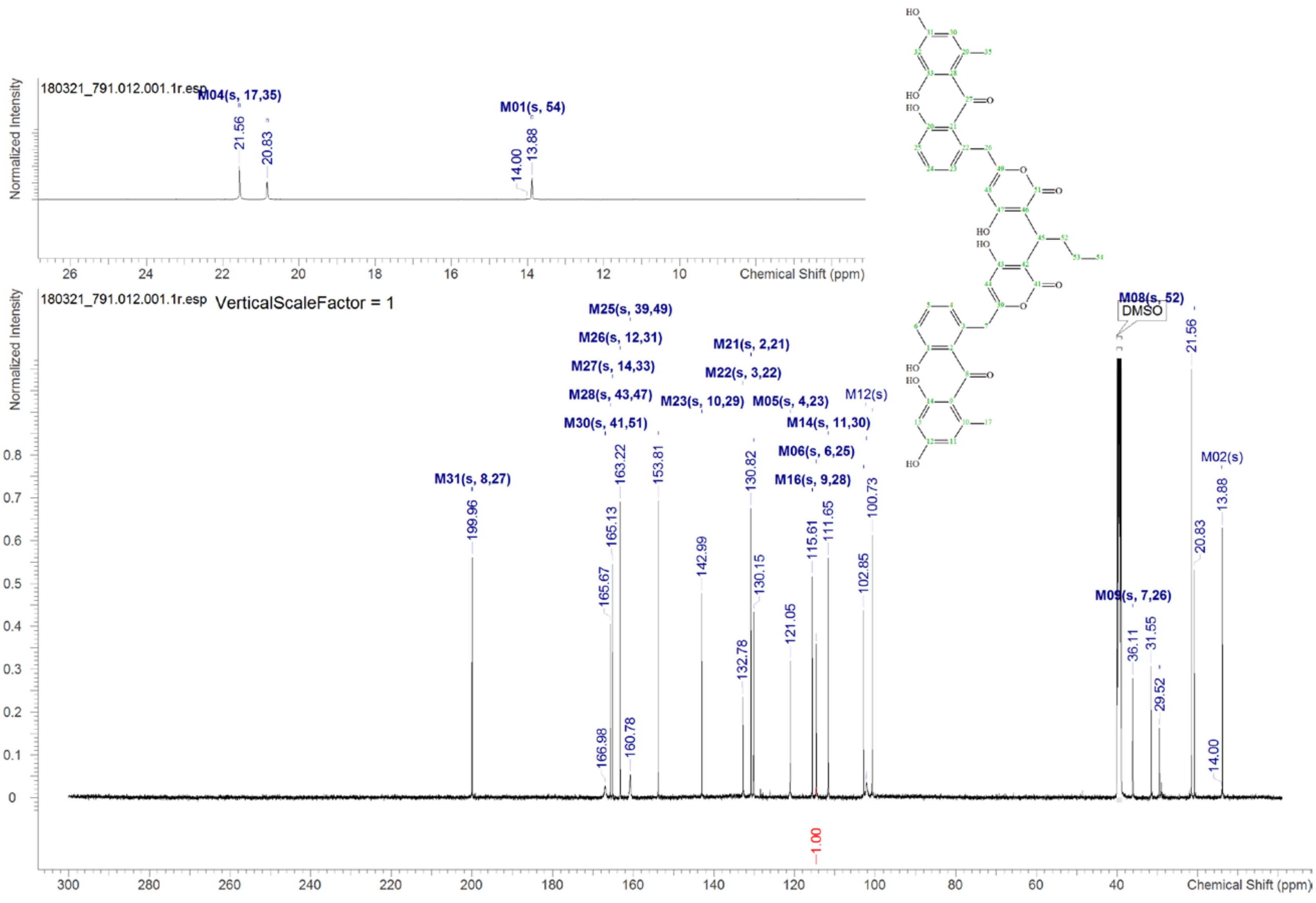

Figure S 38 ^{13}C-NMR spectrum (125 MHz, DMSO-d$_6$) of SEK90; complete Spectrum and zoom from 26 to 6 ppm

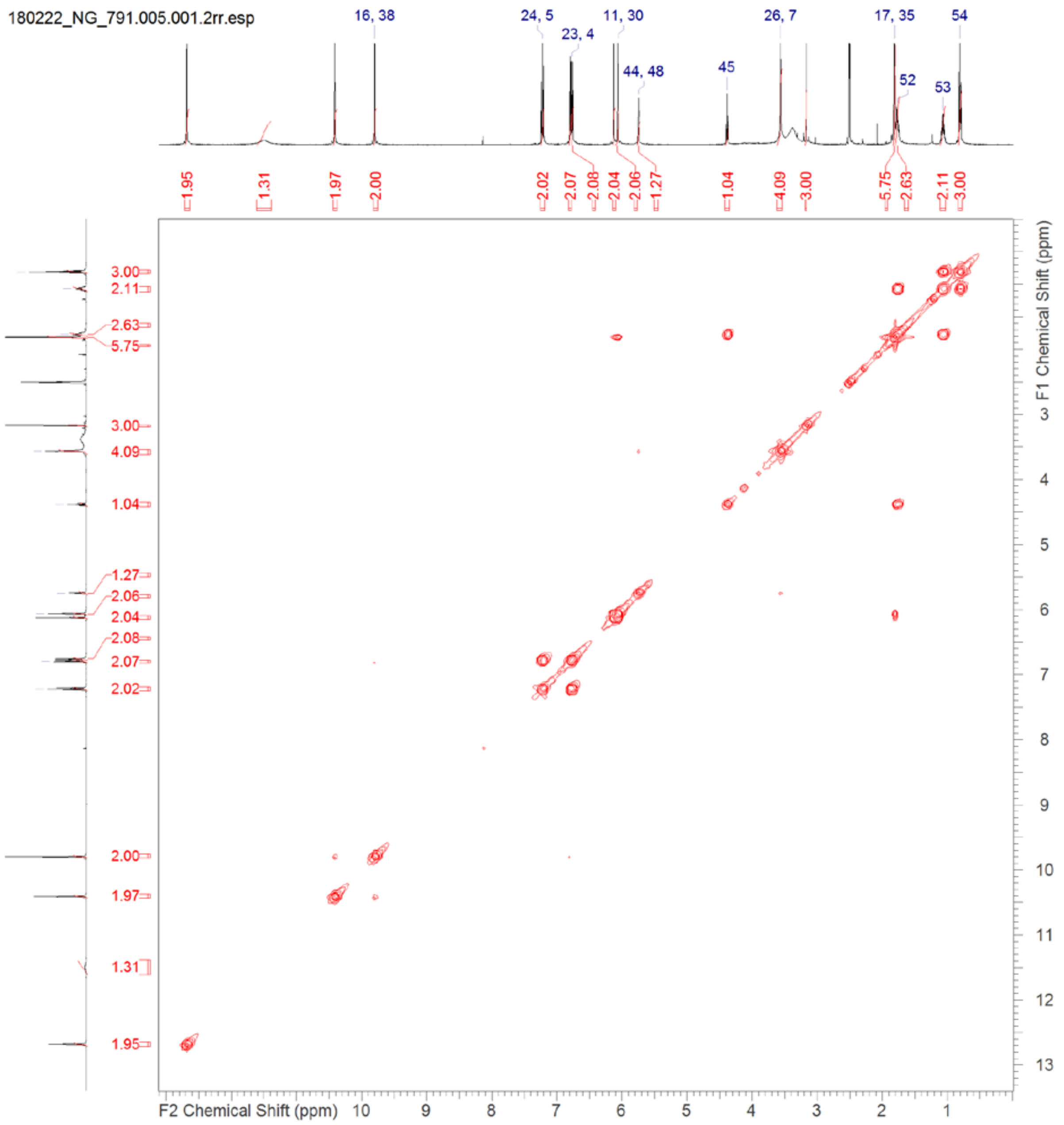

Figure S 39 ^{1}H-^{1}H- COSY spectrum (500 MHz, DMSO-d_6) of SEK90

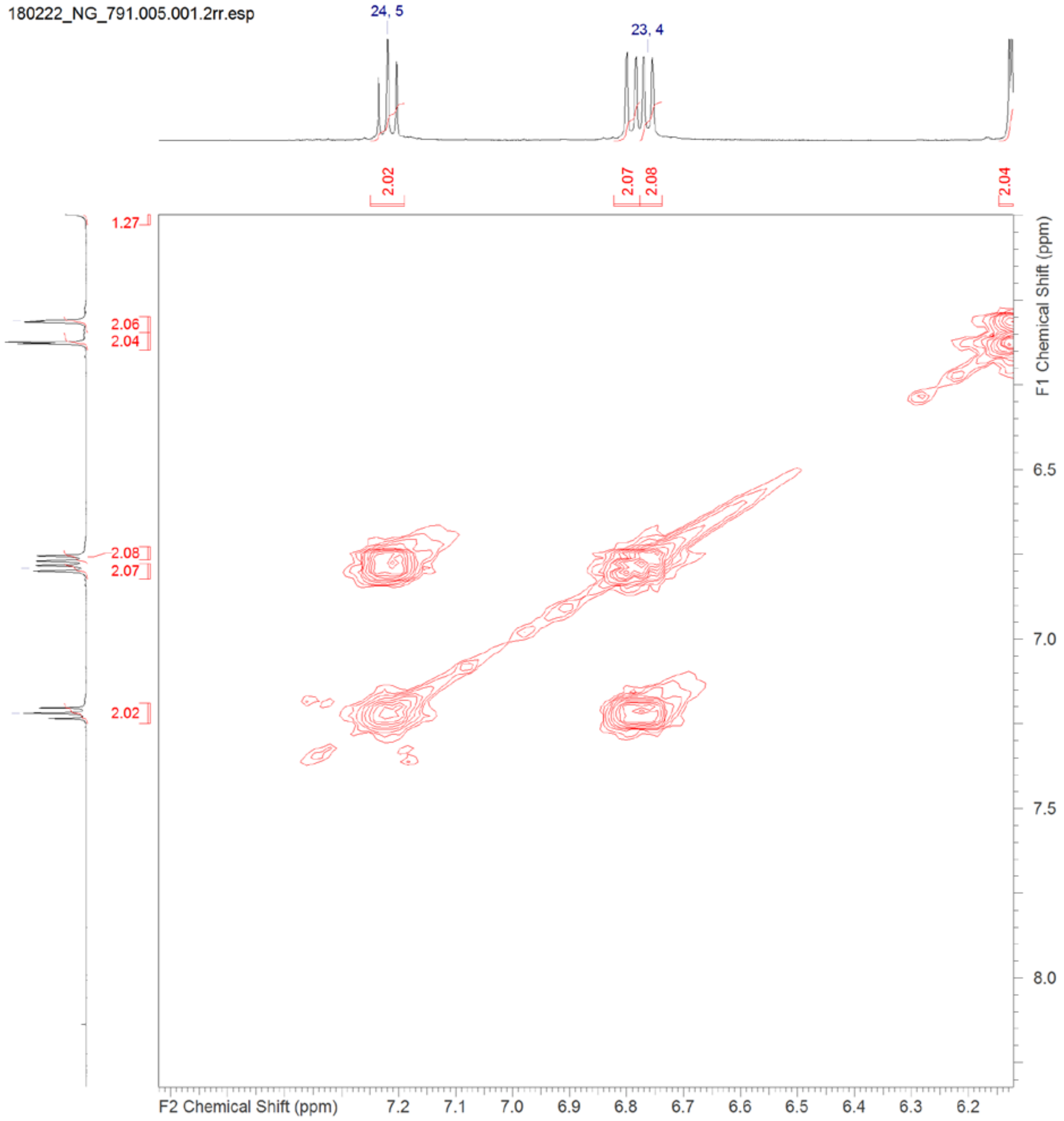

Figure S 40 ^{1}H-^{1}H- COSY spectrum (500 MHz, DMSO-d_6) of SEK90; zoom from 8 to 6.2 ppm

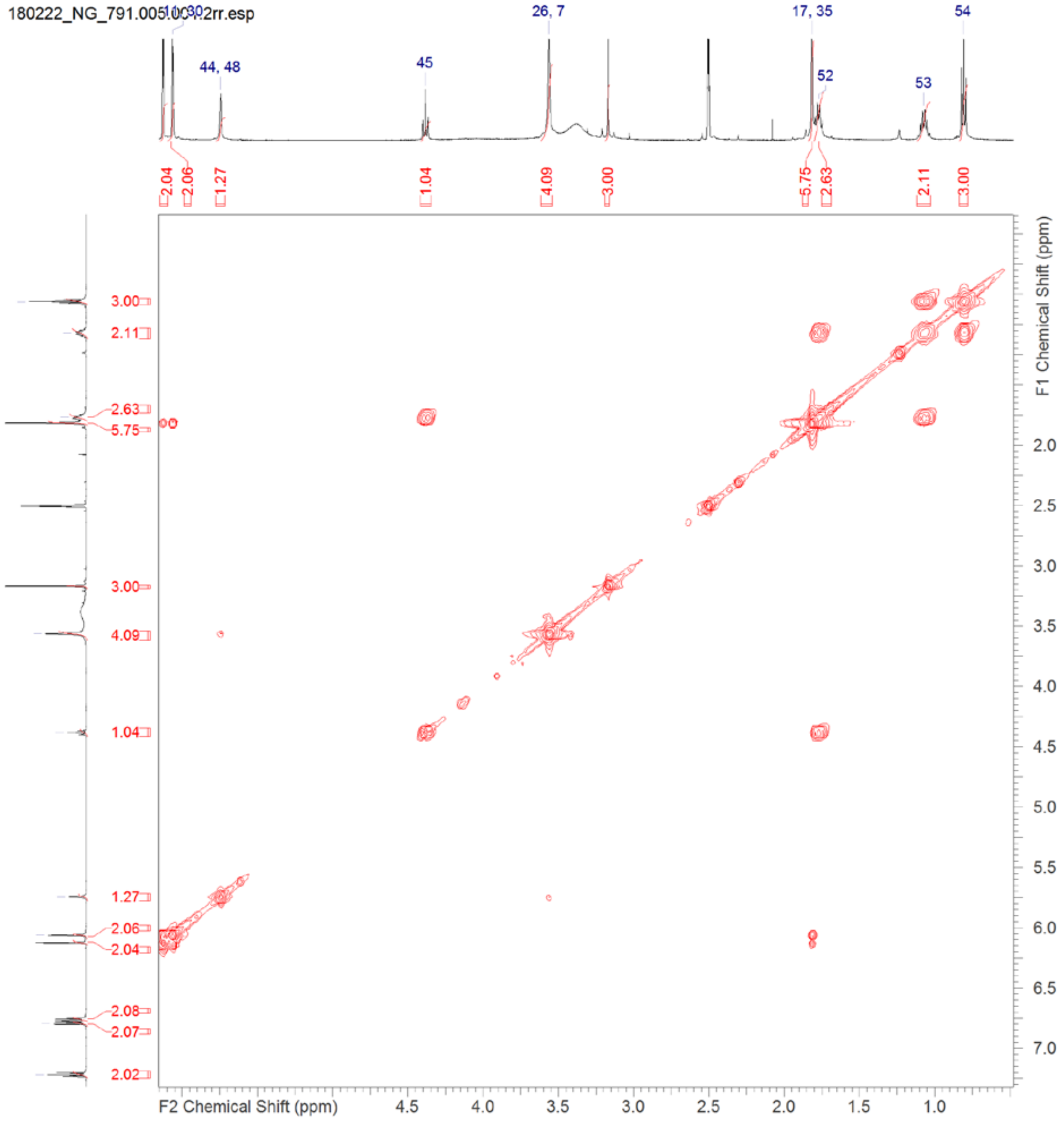

Figure S 41 ^{1}H-^{1}H- COSY spectrum (500 MHz, DMSO-d_6) of SEK90; zoom from 7 to 0.5 ppm

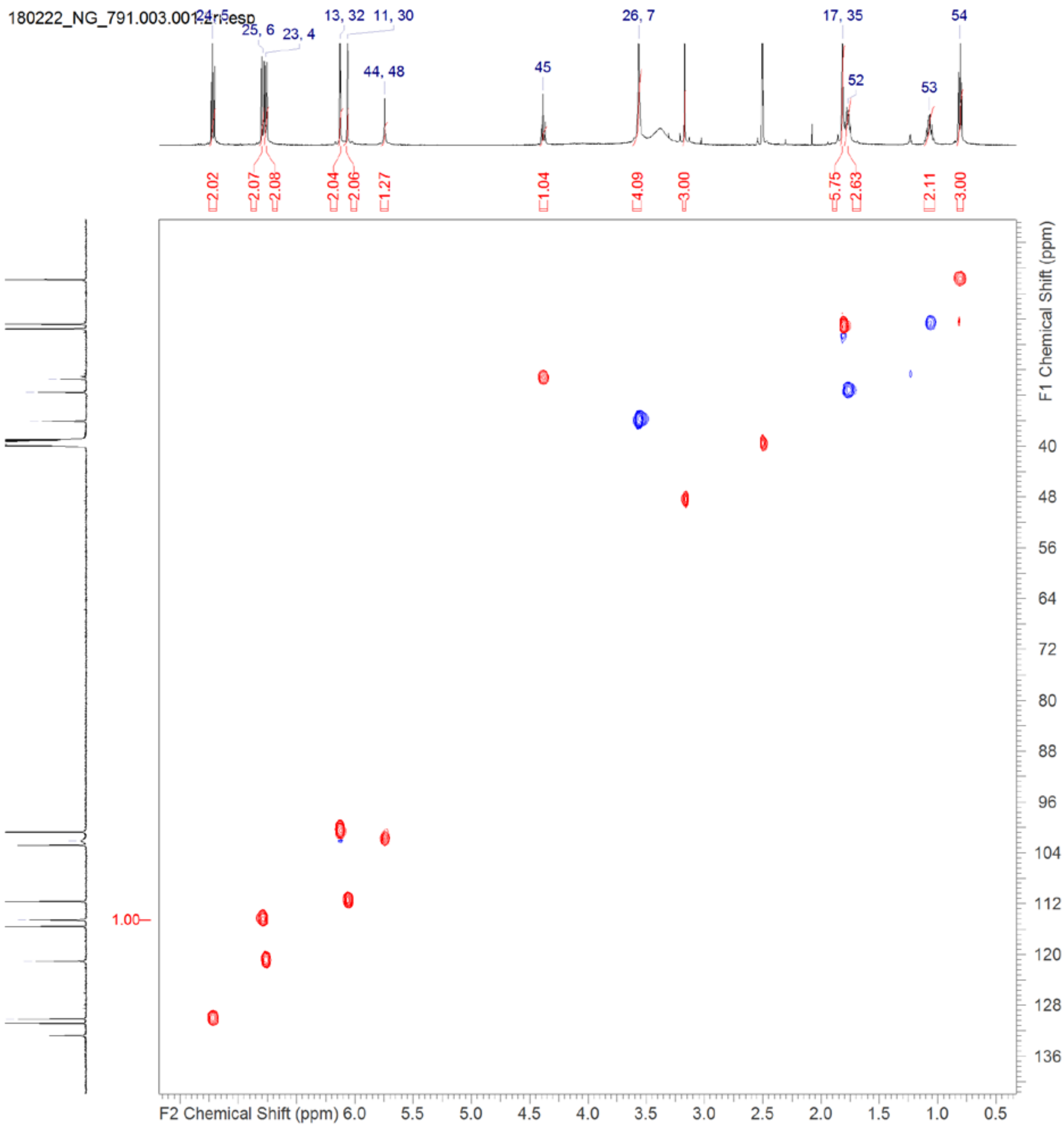

Figure S 42 HSQC-spectrum (500 MHz; 125 MHz, DMSO-d_6) of SEK90

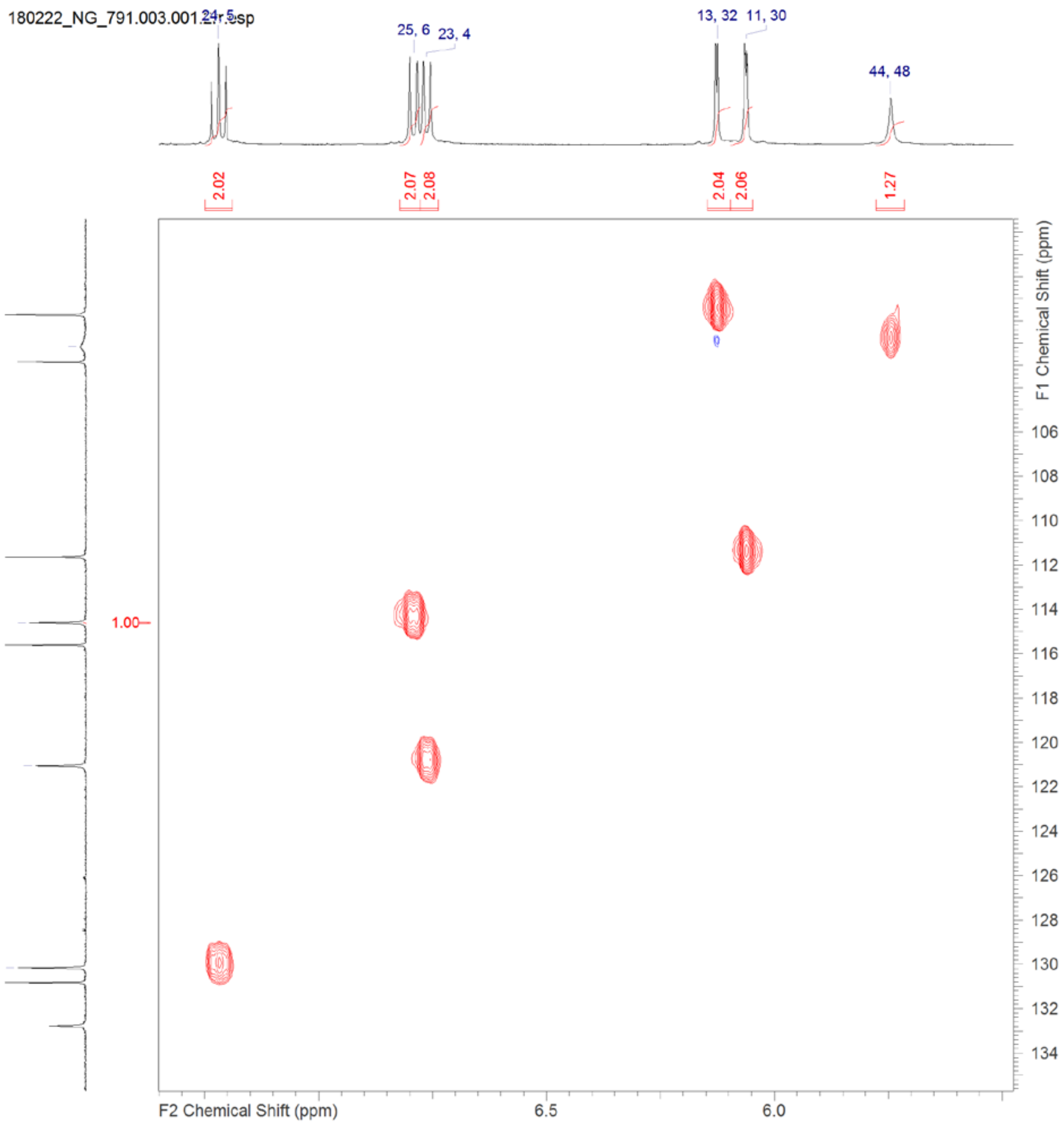

Figure S 43 HSQC-spectrum (500 MHz; 125 MHz, DMSO-d$_6$) of SEK90; zoom from 7.5 to 5.5 ppm and 135 to 99 ppm

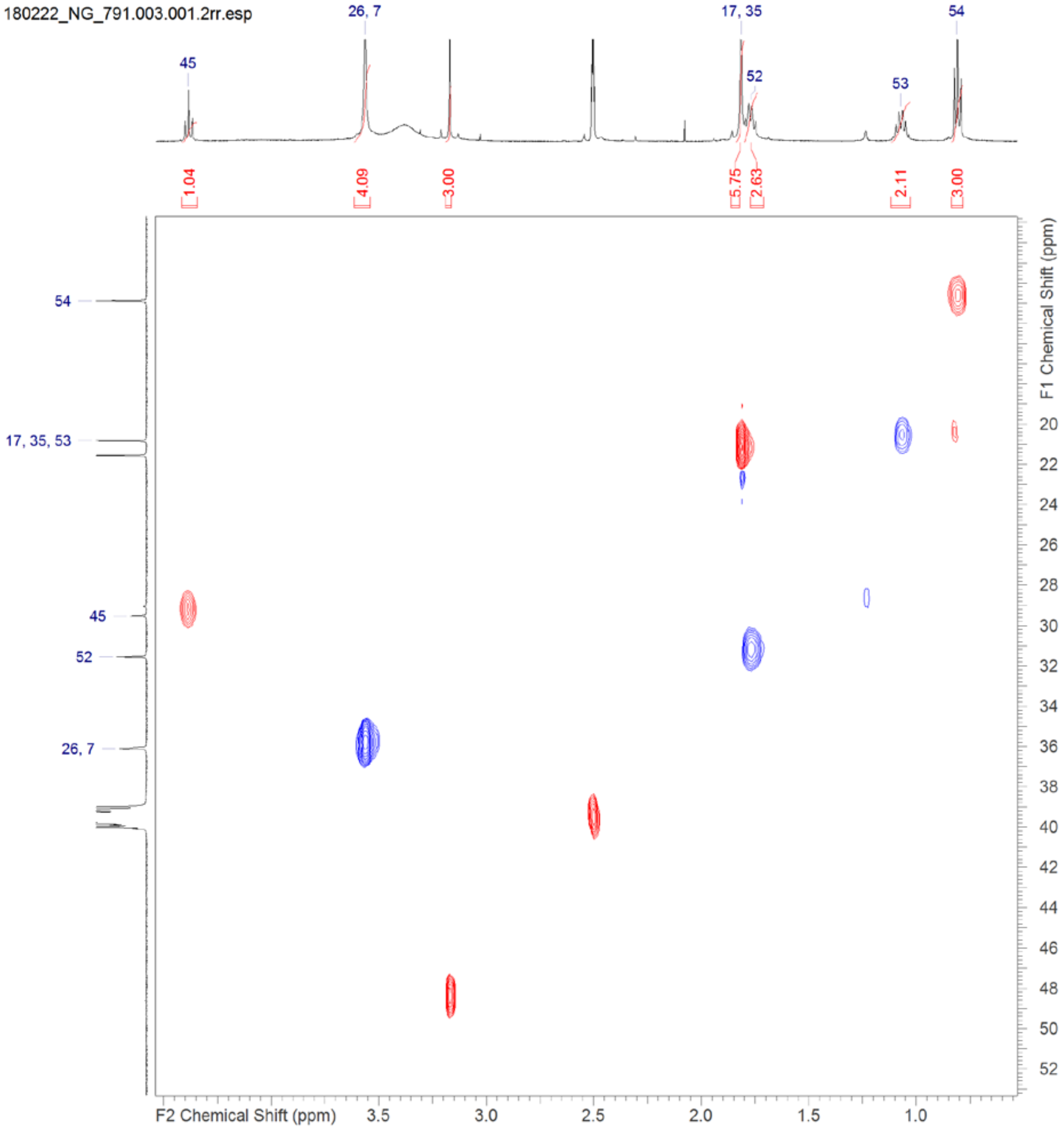

Figure S 44 HSQC-spectrum (500 MHz; 125 MHz, DMSO-d$_6$) of SEK90; zoom from 4 to 0,5 ppm and 53 to 16 ppm

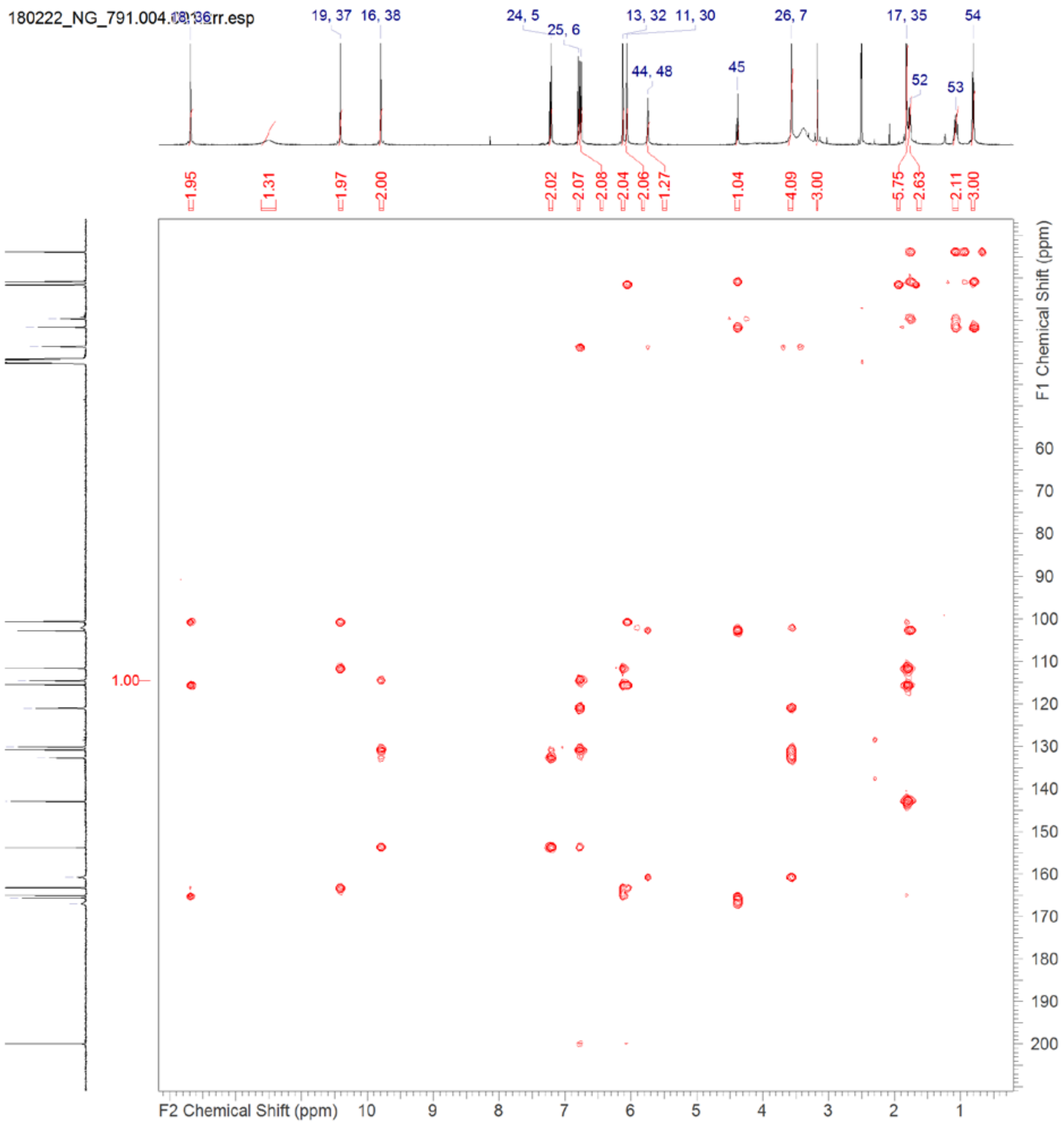

Figure S 45 HSQC-spectrum (500 MHz; 125 MHz, DMSO-d_6) of SEK90; zoom from 4 to 0.5 ppm and 53 to 16 ppm

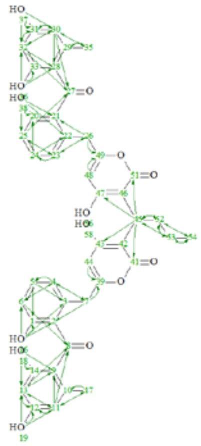

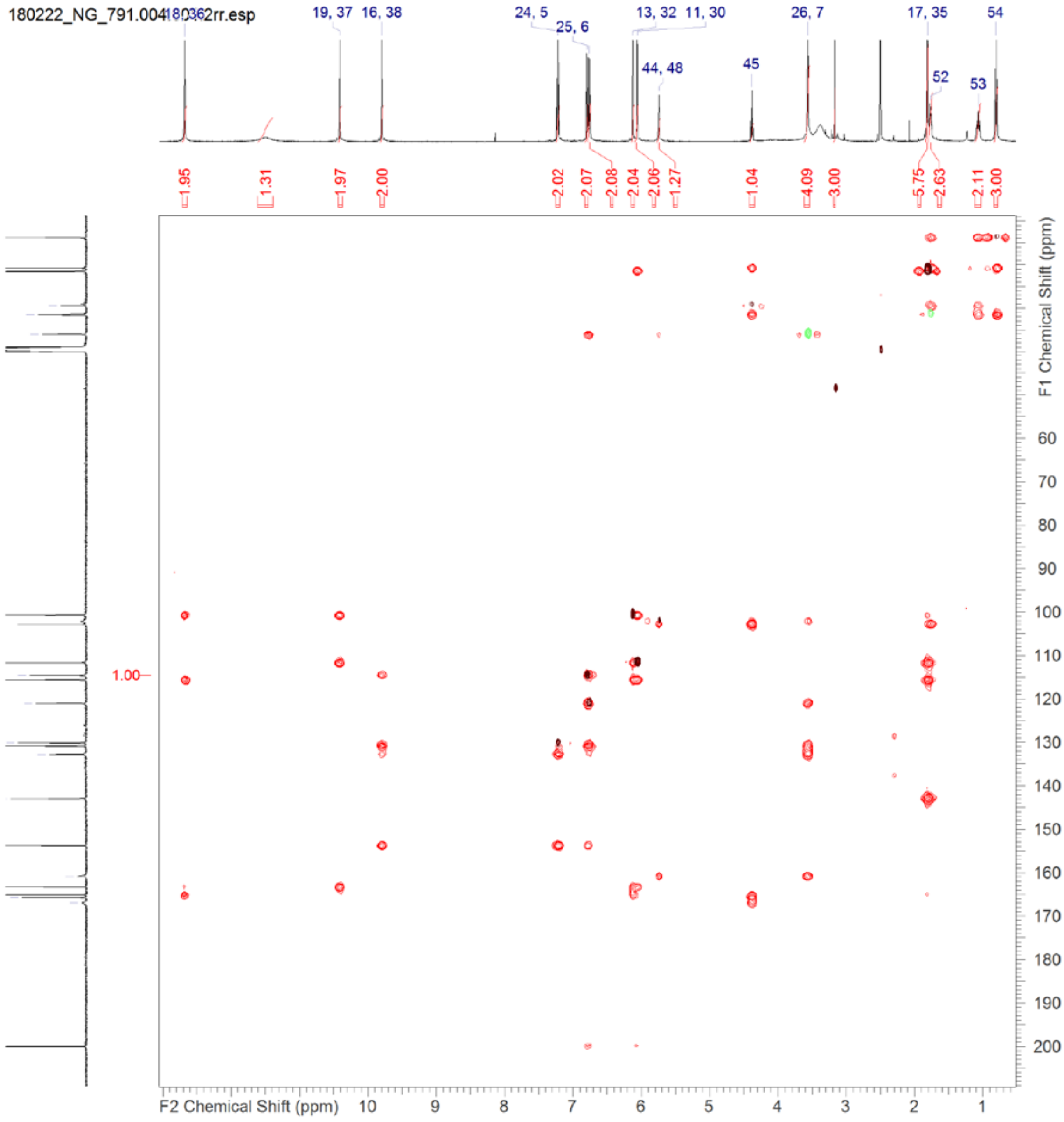

Figure S 46 HMBC - HSQC-Overlay spectrum (500 MHz; 125 MHz, DMSO-d$_6$) of SEK90 (red: HMBC; black/green: HSQC)

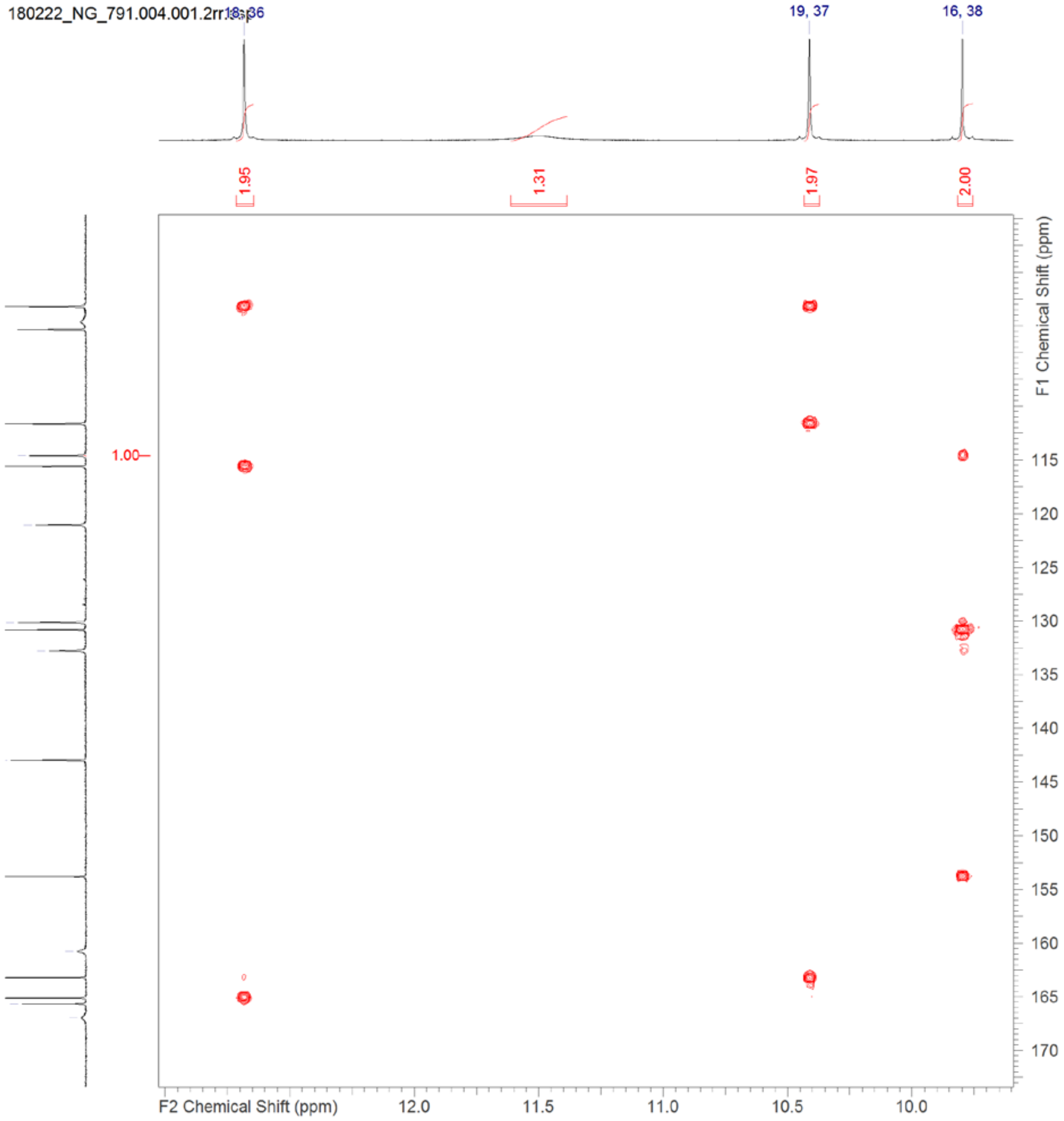

Figure S 47 HMBC - HSQC-Overlay spectrum (500 MHz; 125 MHz, DMSO-d_6) of SEK90 (black: HMBC; red/blue: HSQC); zoom from 13 to 10 ppm and 170 to 105 ppm

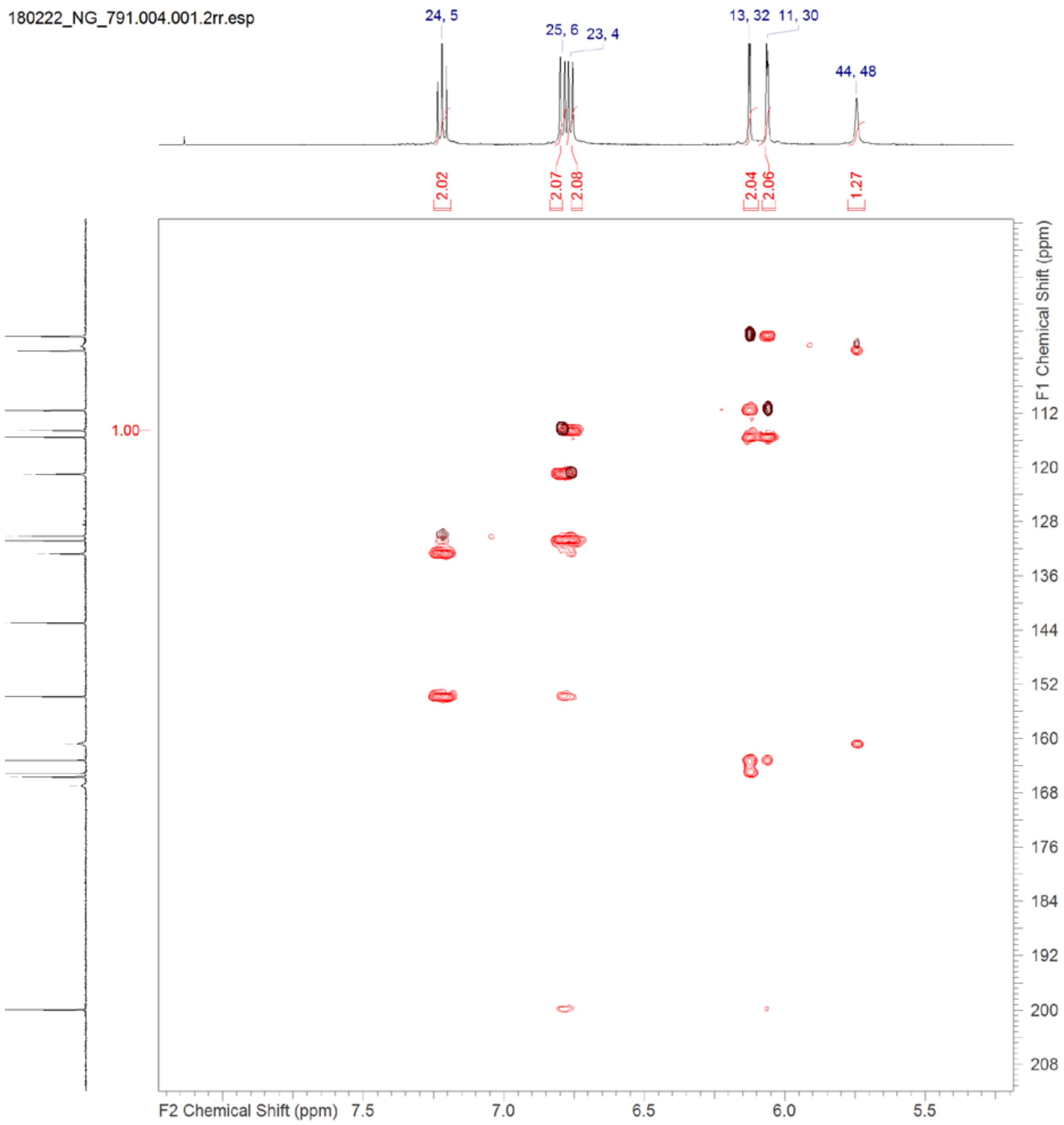

Figure S 48 HMBC - HSQC-Overlay spectrum (500 MHz; 125 MHz, DMSO-d$_6$) of SEK90 (black: HMBC; red/blue: HSQC); zoom from 13 to 10 ppm and 170 to 105 ppm

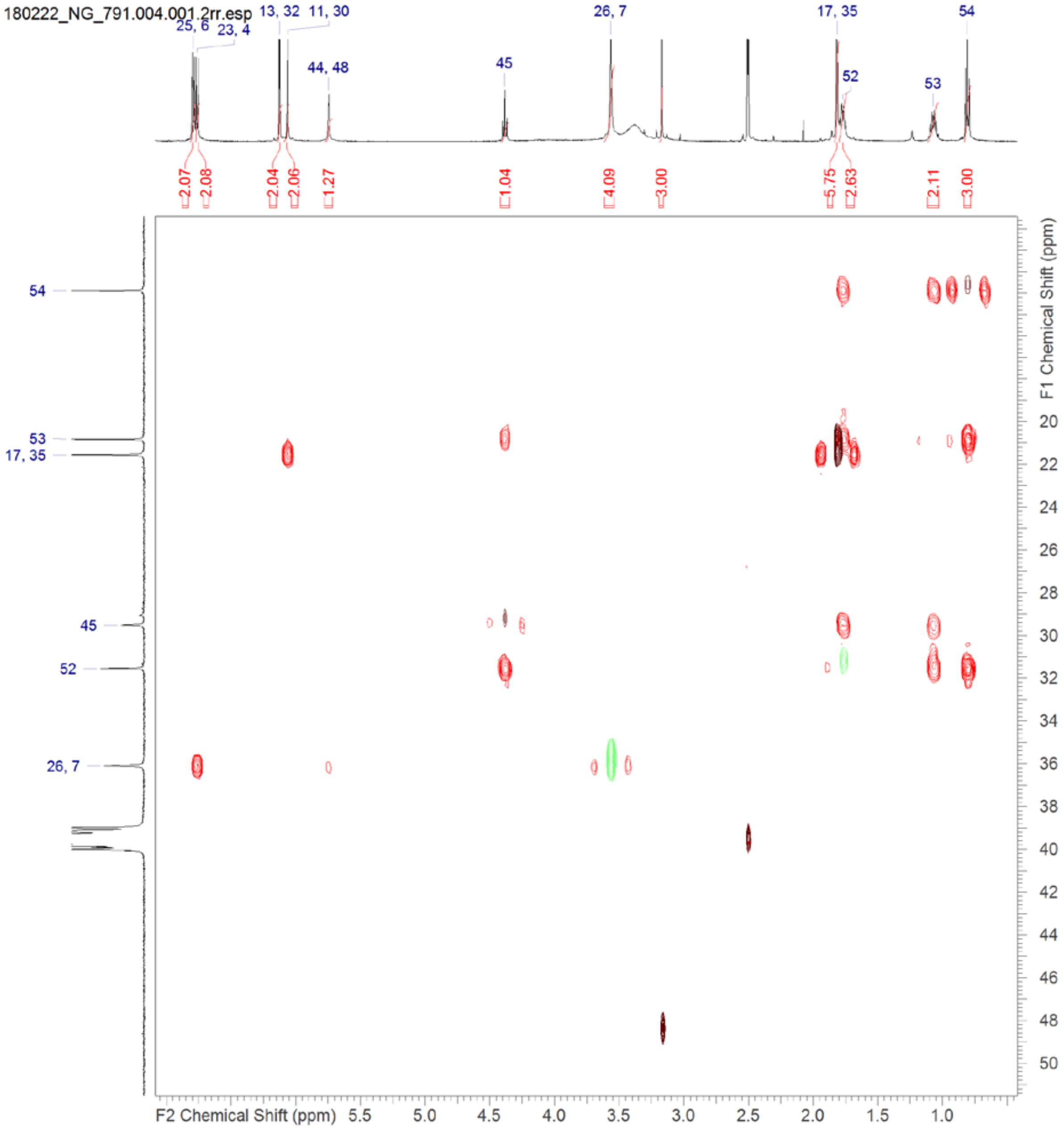

Figure S 49 HMBC - HSQC-Overlay spectrum (500 MHz; 125 MHz, DMSO-d_6) of SEK90 (black: HMBC; red/blue: HSQC); zoom from 6.5 to 0.5 ppm and 51 to 10 ppm

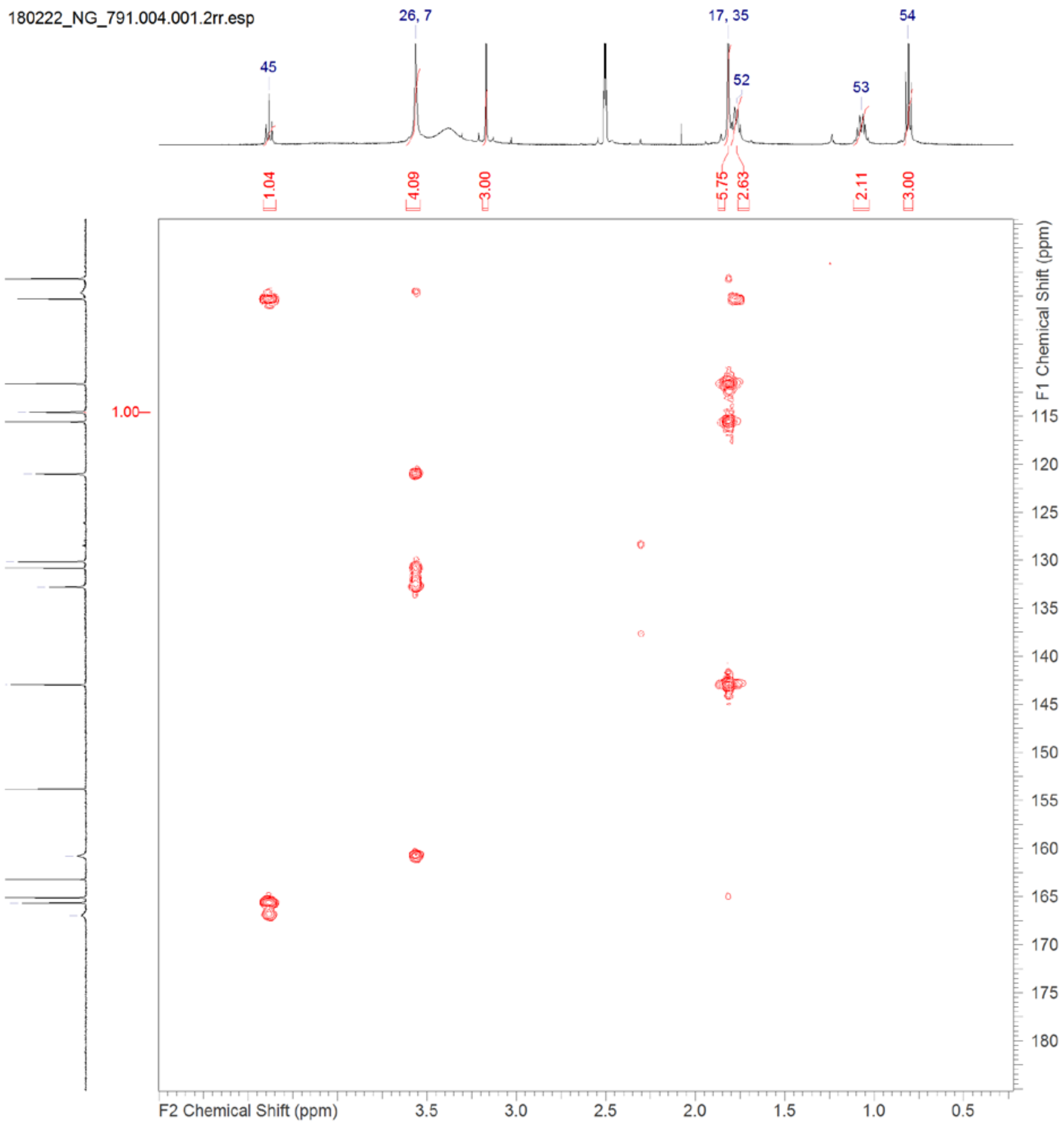

Figure S 50 HMBC - HSQC-Overlay spectrum (500 MHz; 125 MHz, DMSO-d_6) of SEK90 (black: HMBC; red/blue: HSQC); zoom from 4.5 to 0.5 ppm and 185 to 100 ppm

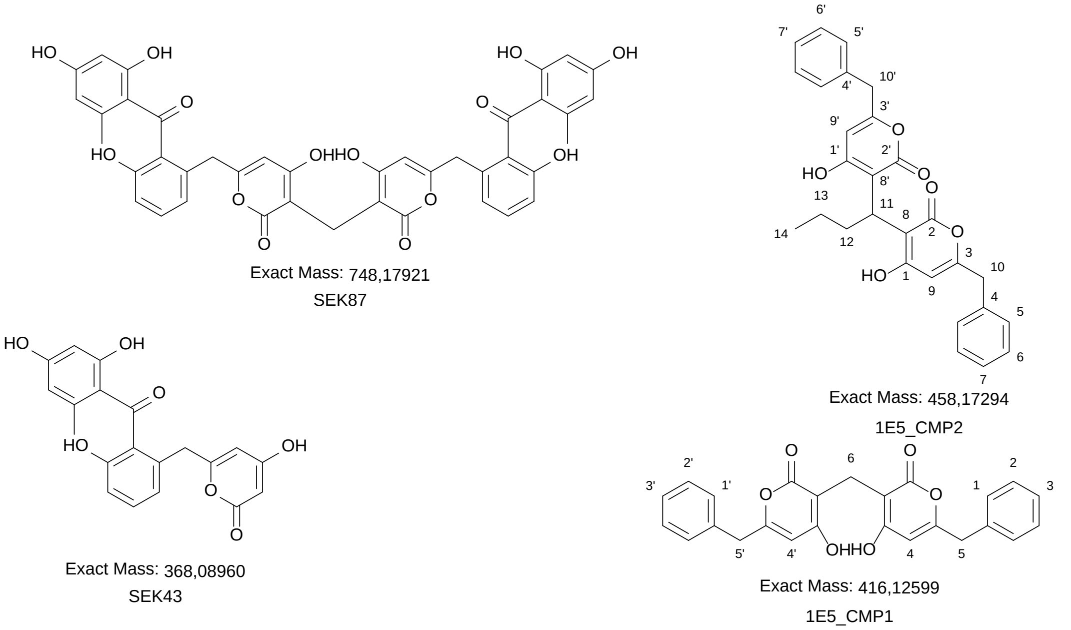

Figure S 51 Structures of SEK87, SEK43 and 1E5_CMP1 & 1E5_CMP2

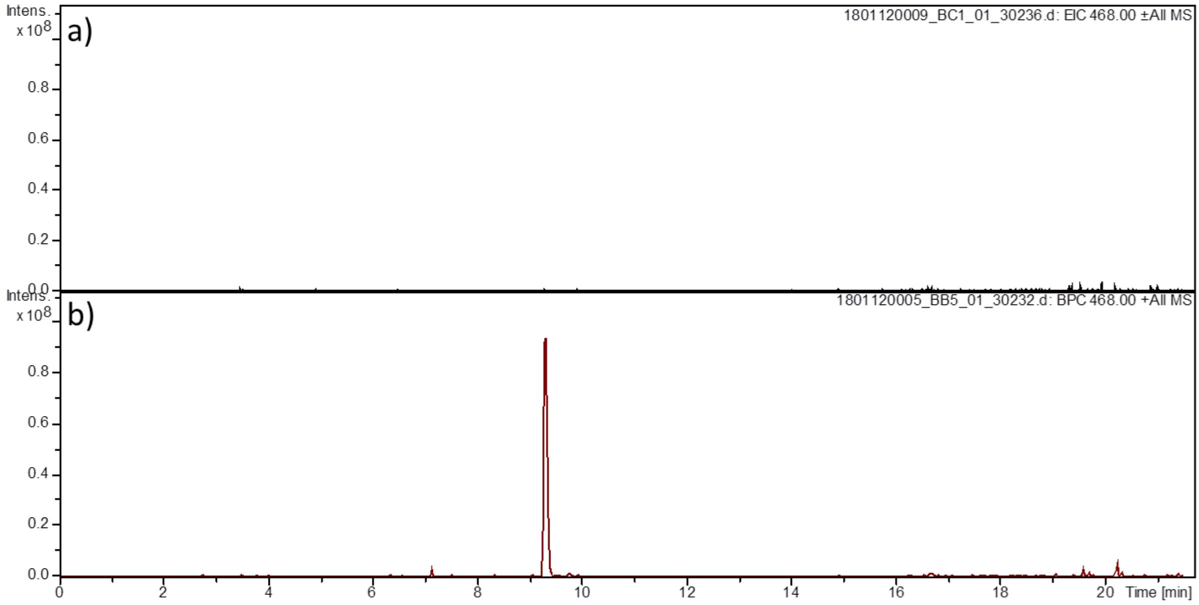

Figure S 52 HPLC-MS Extracted ion chromatogram (Extracted mass 468±0.5) of a) S. lividans ΔYA6_1E5Δ54860 and b) S. lividans ΔYA6_1E5Δ55110; The production is unchanged in the deletion of the NRPS/PKSI hybrid core gene, while the production is abolished in the created mutant lacking the type II PKS gene

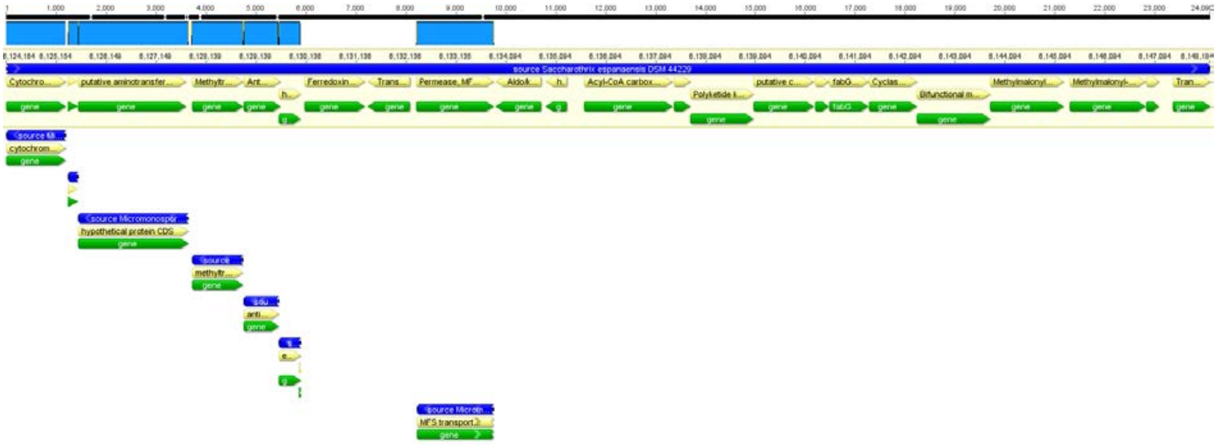

Figure S 53 Genes (WP_124773686 & WP_124773691-WP124773694) of Micromonospora sp. LB39 mapped to Pentangumycins biosynthetic gene cluster

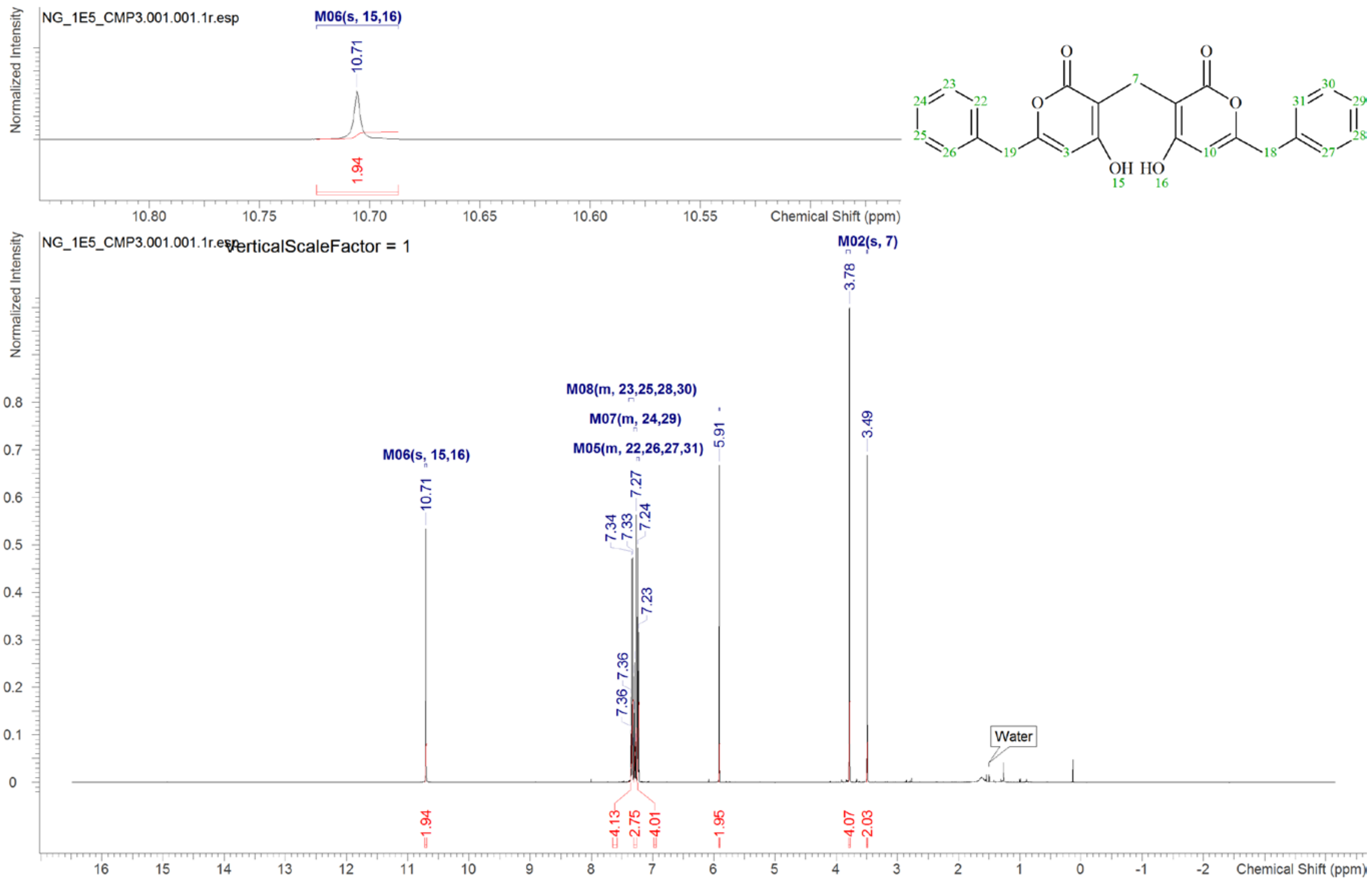

Figure S 54 ¹H-NMR spectrum (500 MHz, CDCL₃) of 1E5_CMP1; zoom from 10.8 ppm to 10.5 ppm

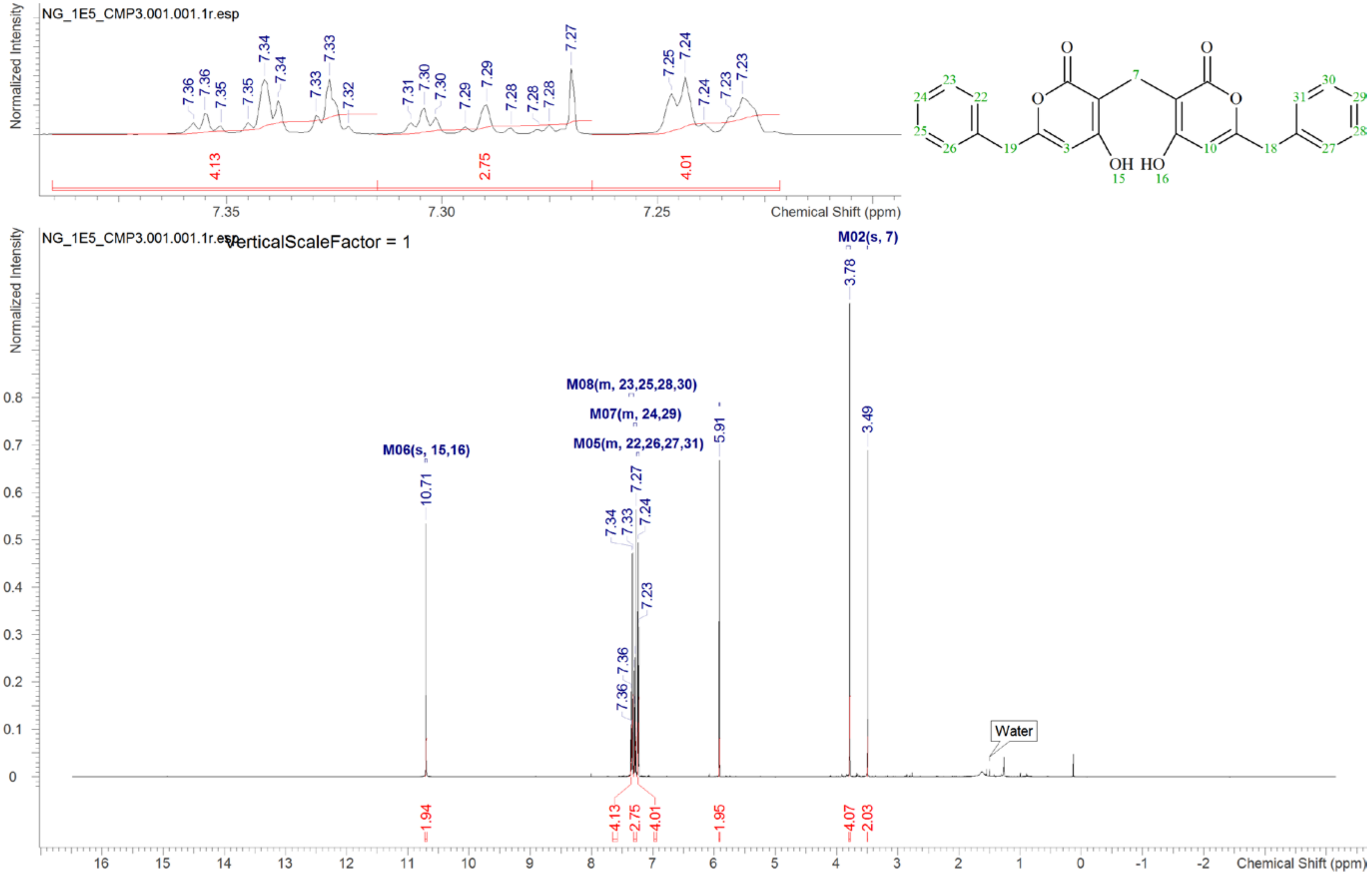

Figure S 55 ¹H-NMR spectrum (500 MHz, CDCL₃) of 1E5_CMP1; zoom from 7.4 ppm to 7.2 ppm

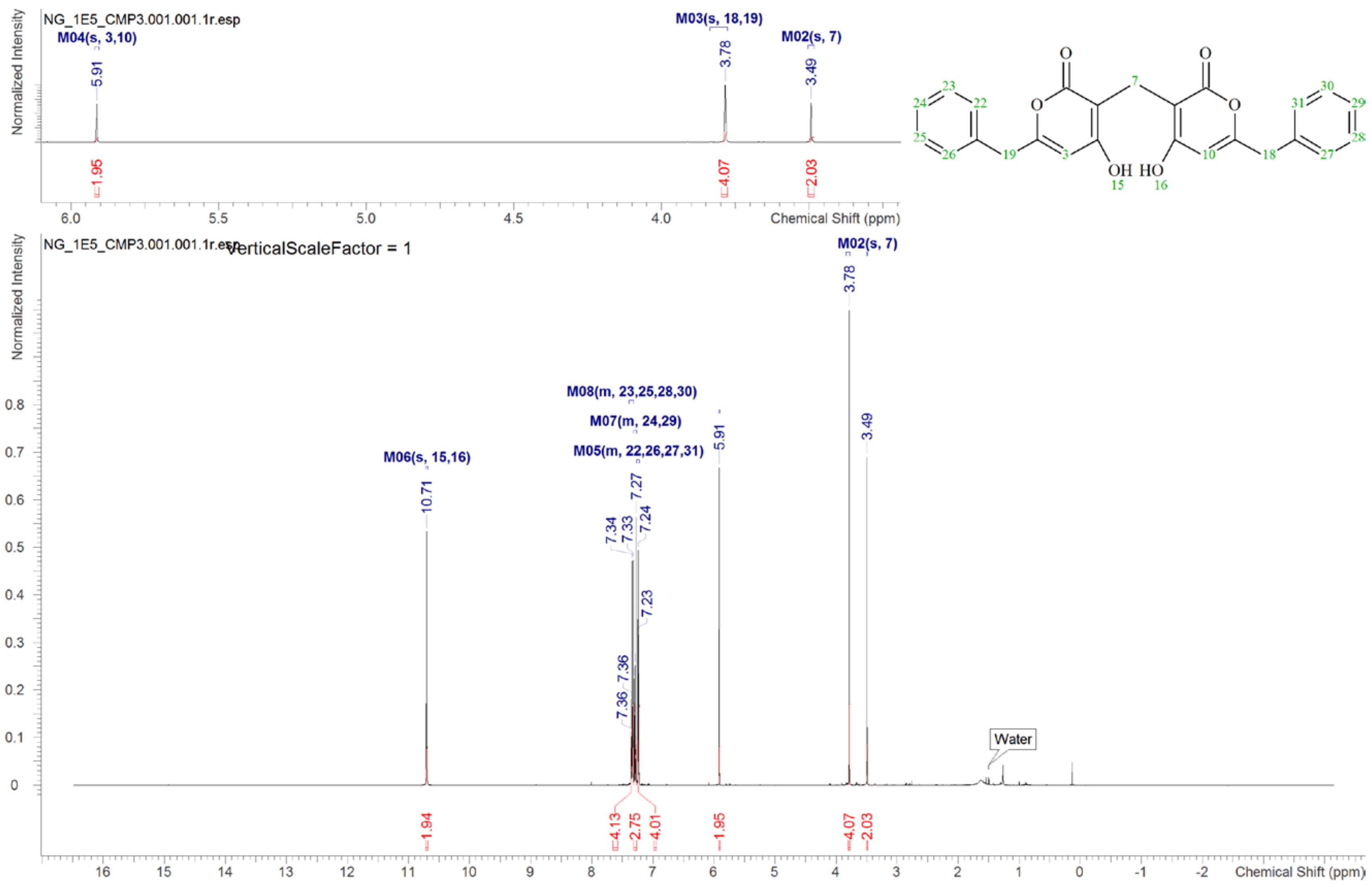

Figure S 56 ¹H-NMR spectrum (500 MHz, CDCL₃) of 1E5_CMP1; zoom from 6 ppm to 3.5 ppm

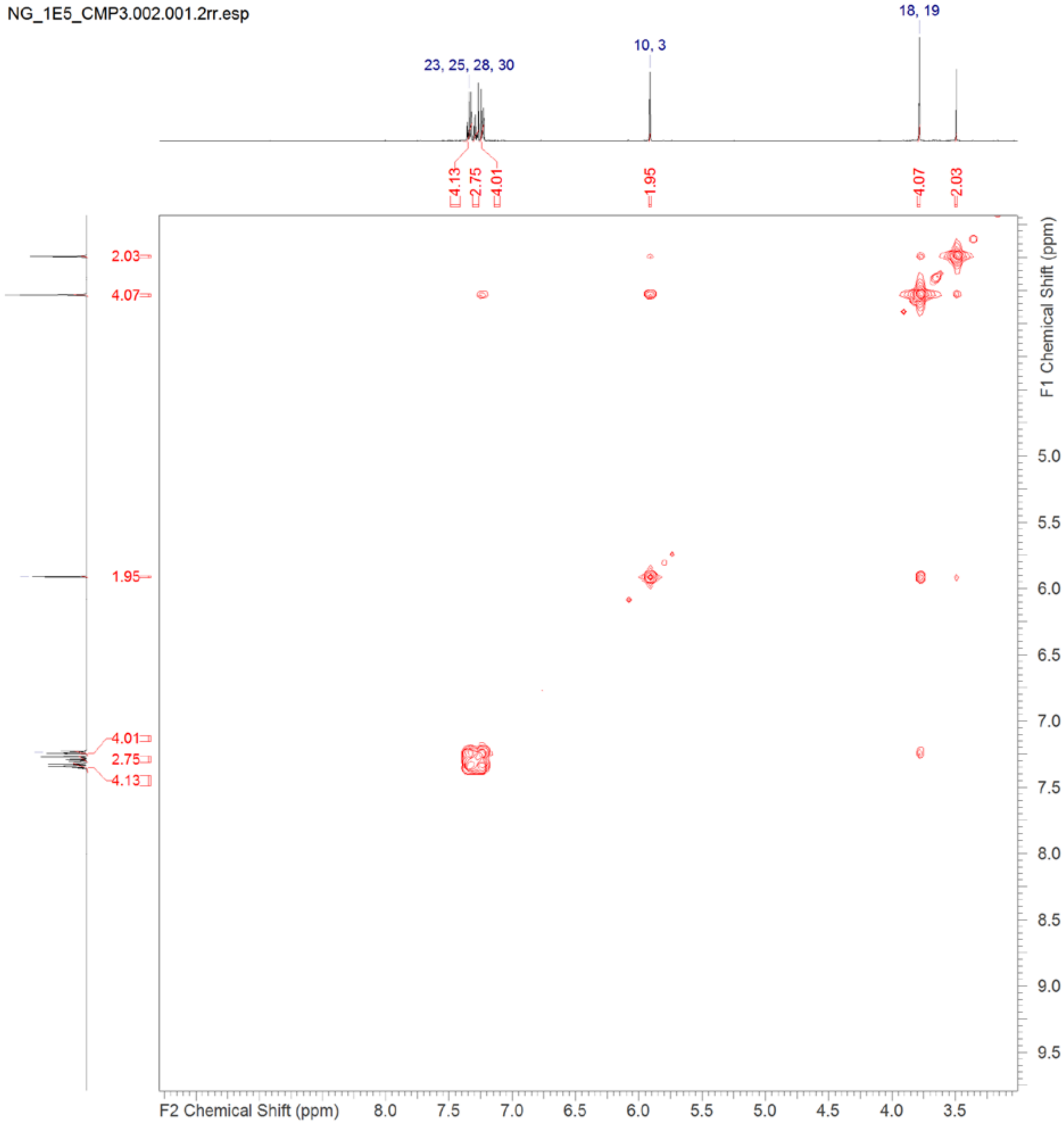

Figure S 57 ^{1}H-^{1}H- COSY spectrum (500 MHz, $CDCL_3$) of 1E5_CMP1

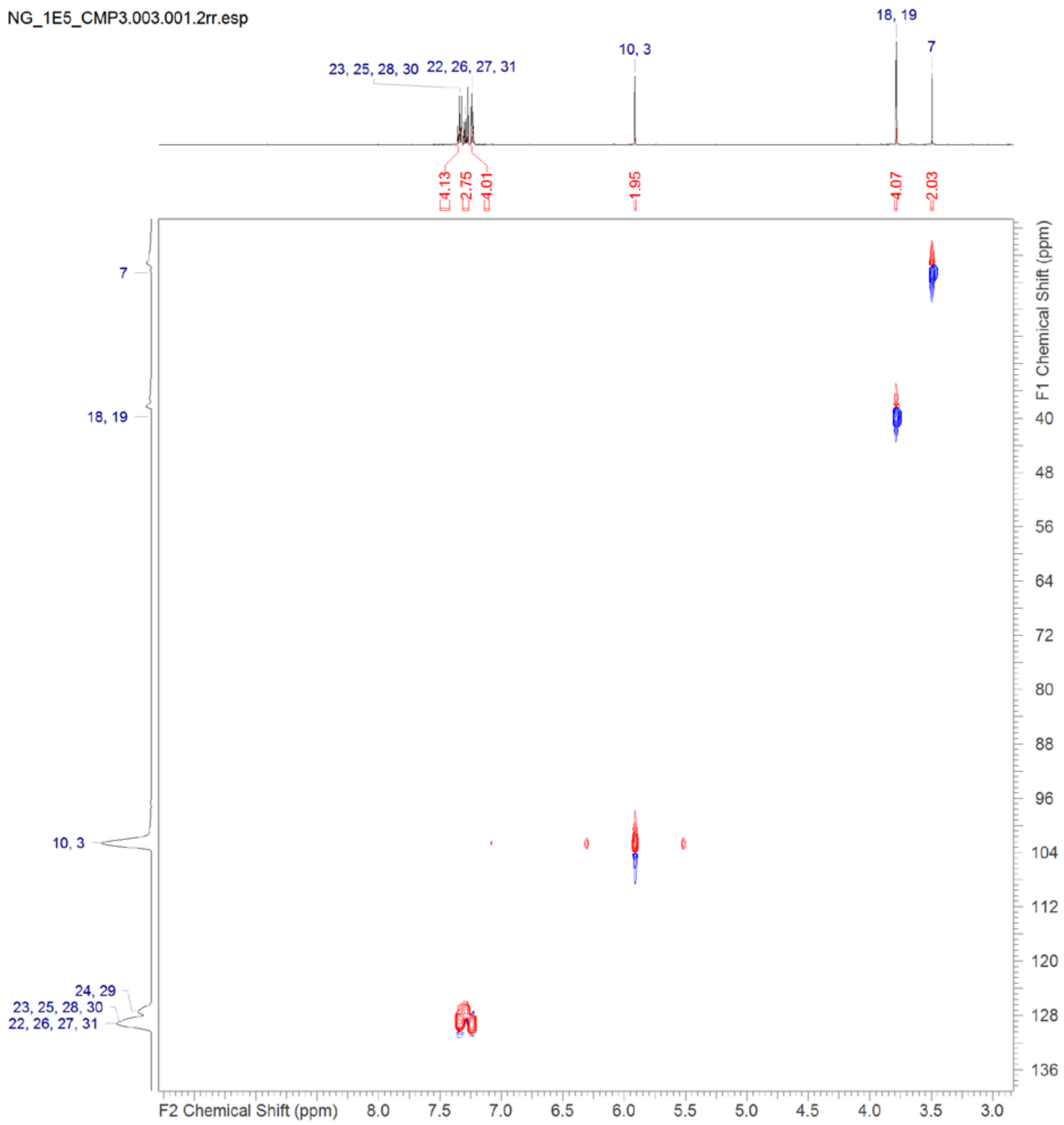

Figure S 58 HSQC-spectrum (500 MHz; 125 MHz, CDCL3) of 1E5_CMP1

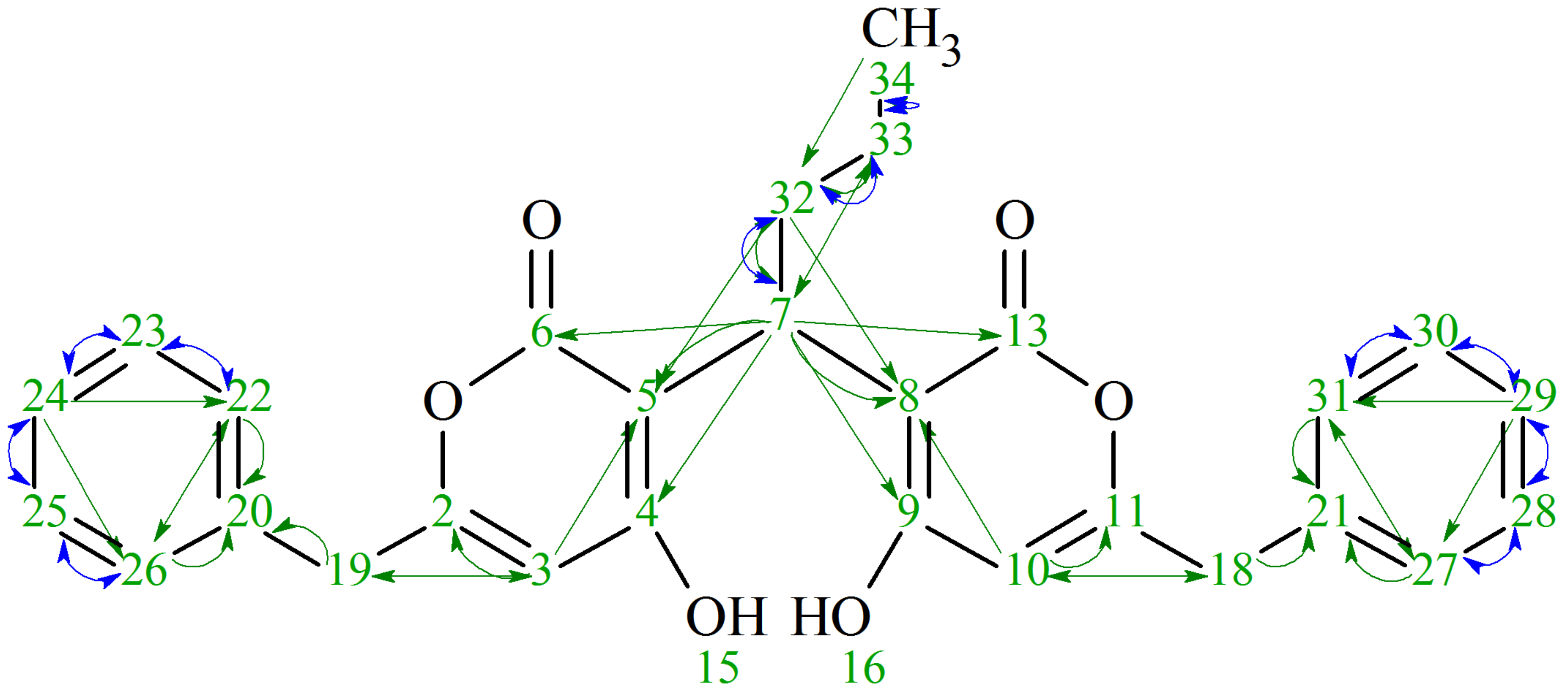

Figure S 59 Structure of 1E5_CMP2 with all obsevered correlations (HMBC: green; ^{1}H-^{1}H-Cosy: blue)

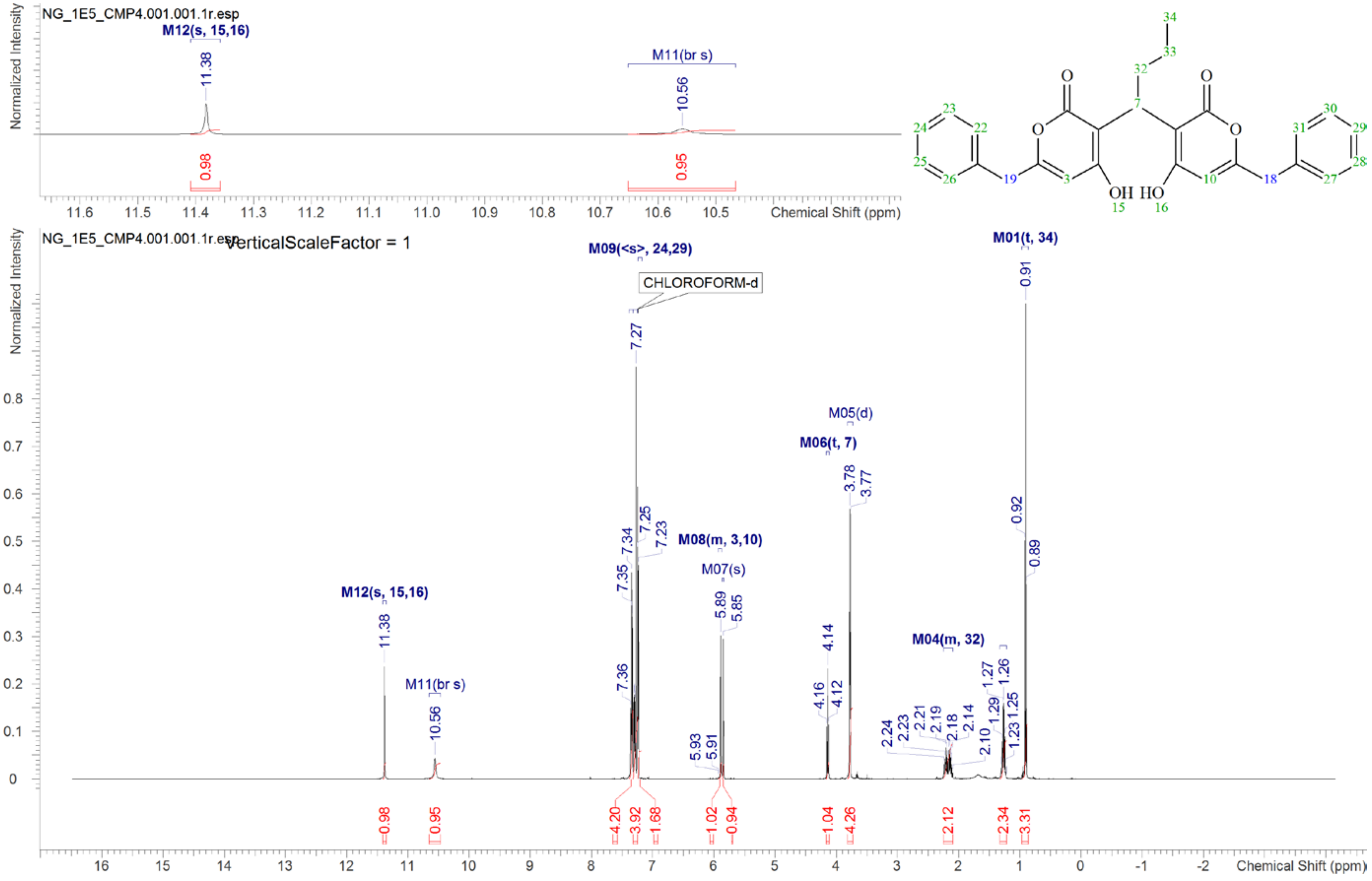

Figure S 60 ^{1}H-NMR spectrum (500 MHz, CDCL₃) of 1E5_CMP2; zoom from 11.6 to 9.5 ppm

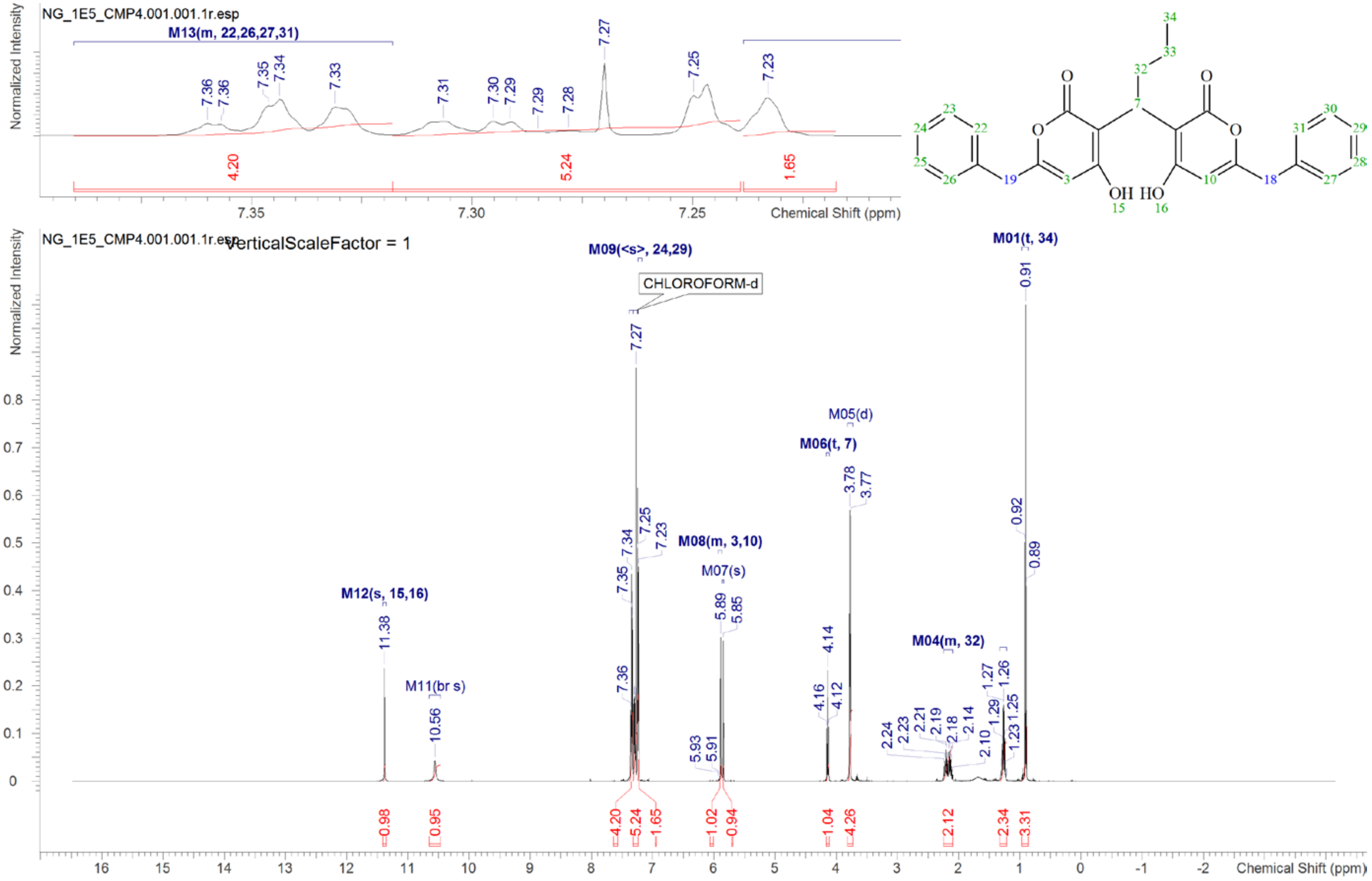

Figure S 61 ^{1}H-NMR spectrum (500 MHz, $CDCL_3$) of 1E5_CMP2; zoom from 7.4 to 7.2 ppm

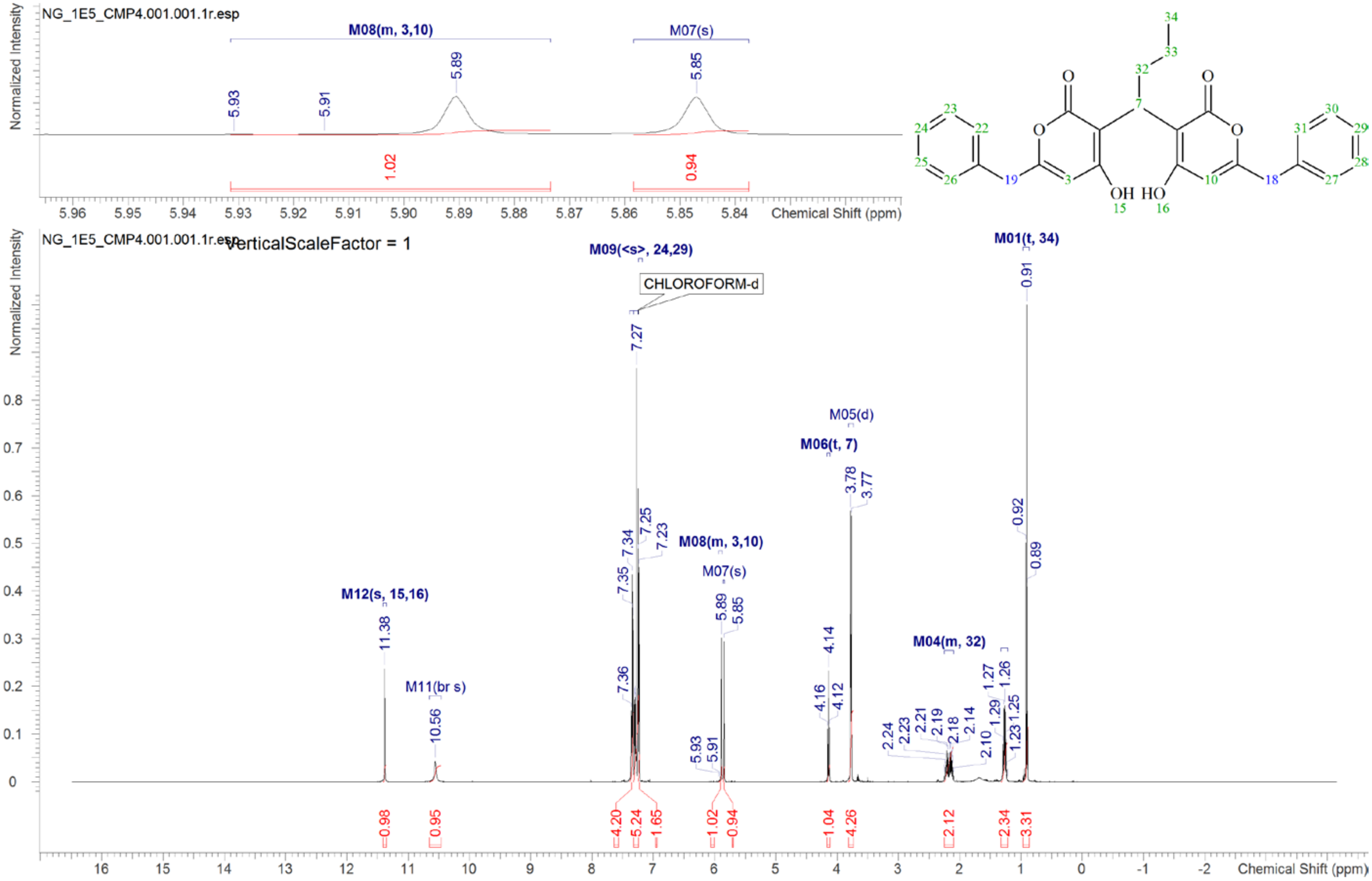

Figure S 62 ^{1}H-NMR spectrum (500 MHz, $CDCL_3$) of 1E5_CMP2; zoom from 5.96 to 5.8 ppm

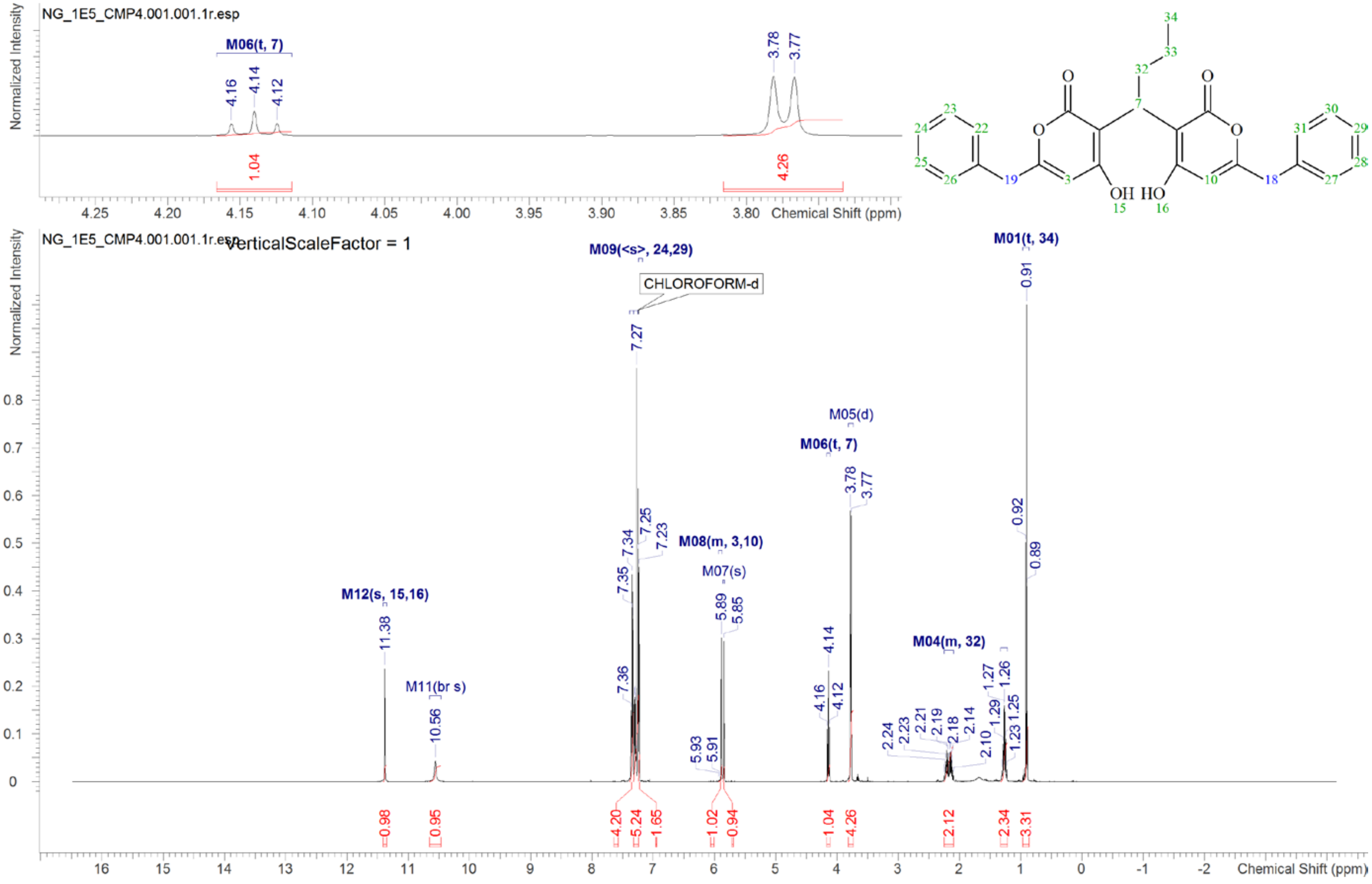

Figure S 63 ^{1}H-NMR spectrum (500 MHz, $CDCL_3$) of 1E5_CMP2; zoom from 4.25 to 3.75 ppm

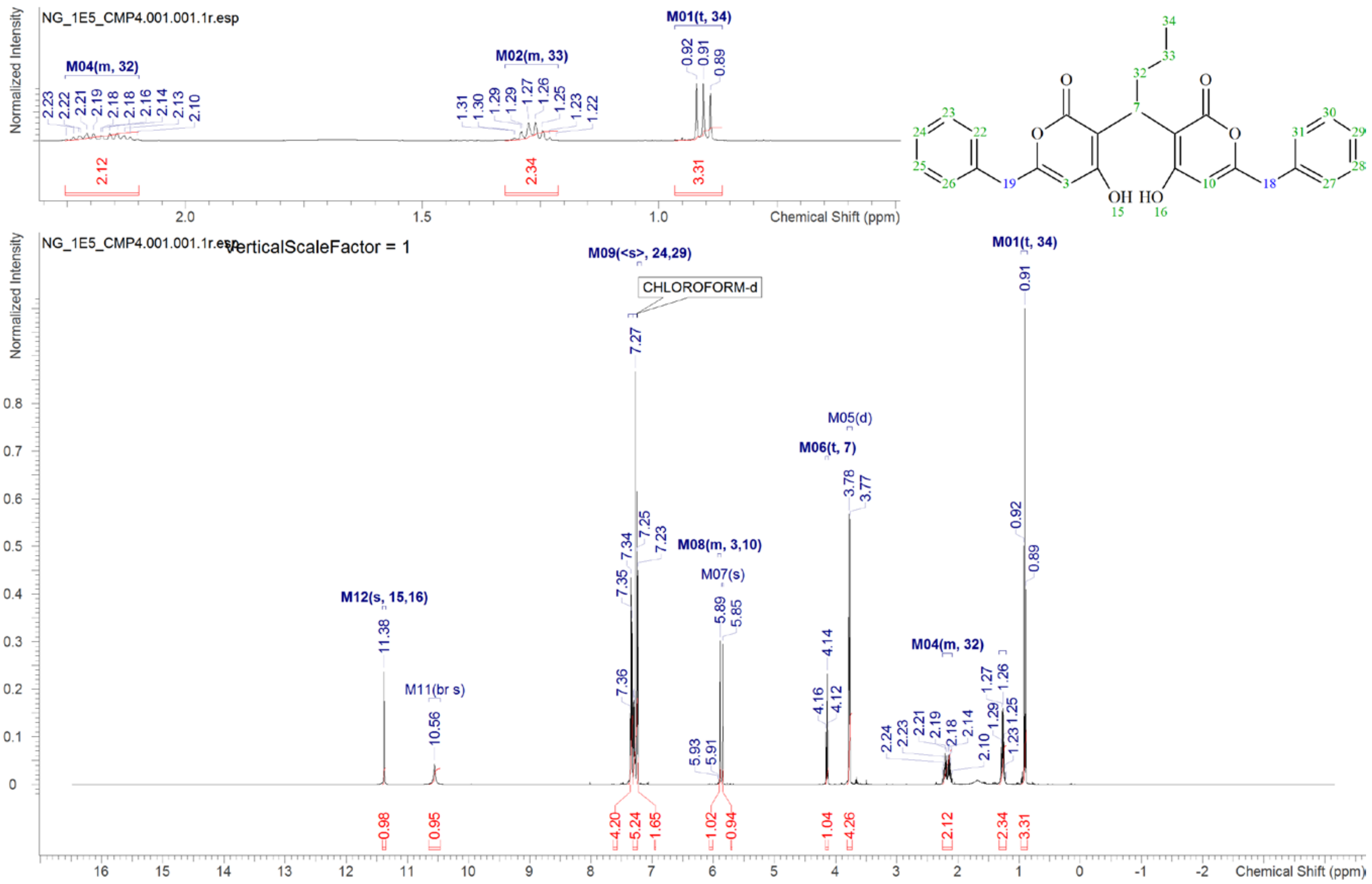

Figure S 64 ¹H-NMR spectrum (500 MHz, CDCL₃) of 1E5_CMP2; zoom from 2.5 to 0.5 ppm

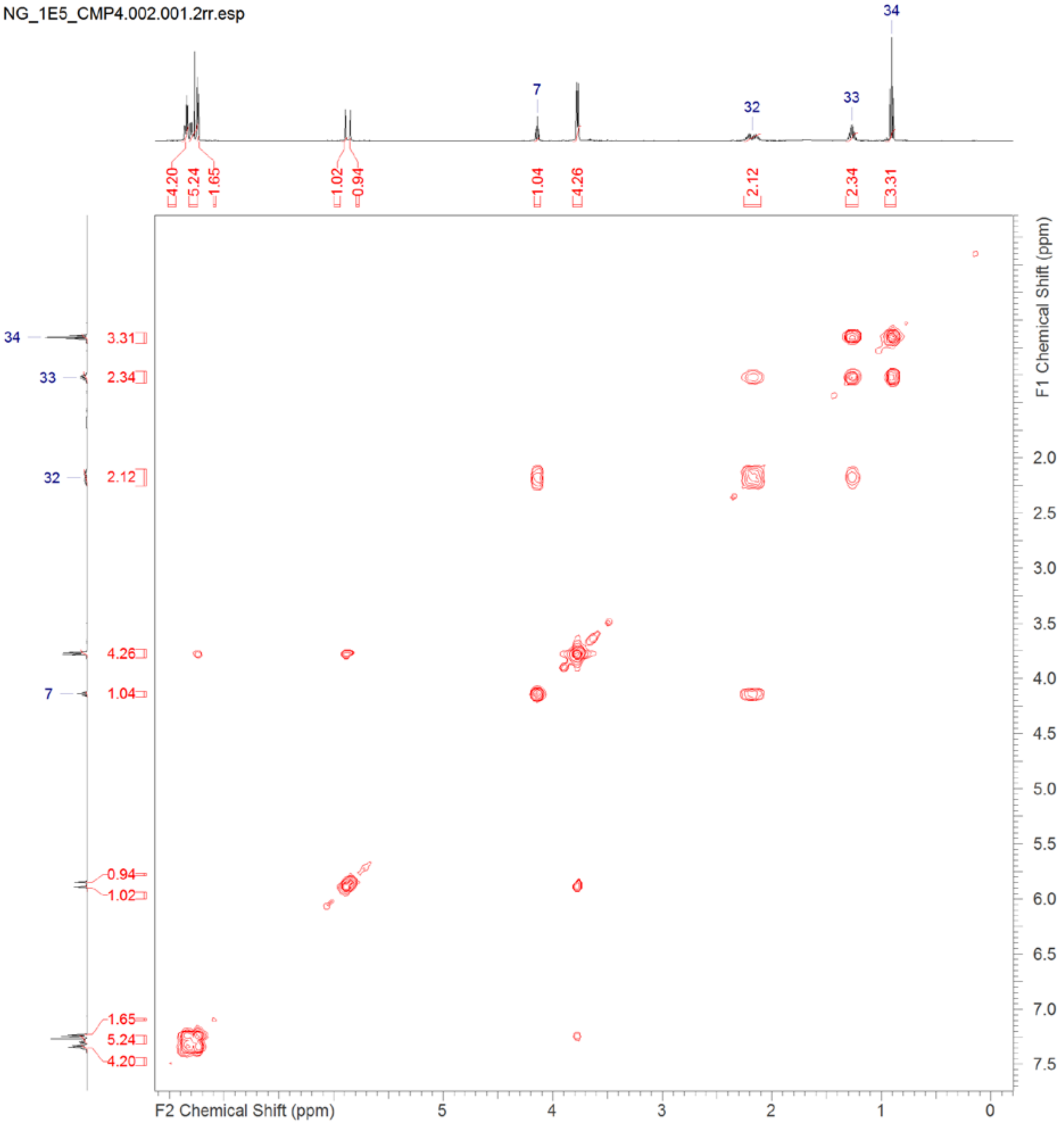

Figure S 65 ^{1}H-^{1}H- COSY spectrum (500 MHz, $CDCL_3$) of 1E5_CMP2

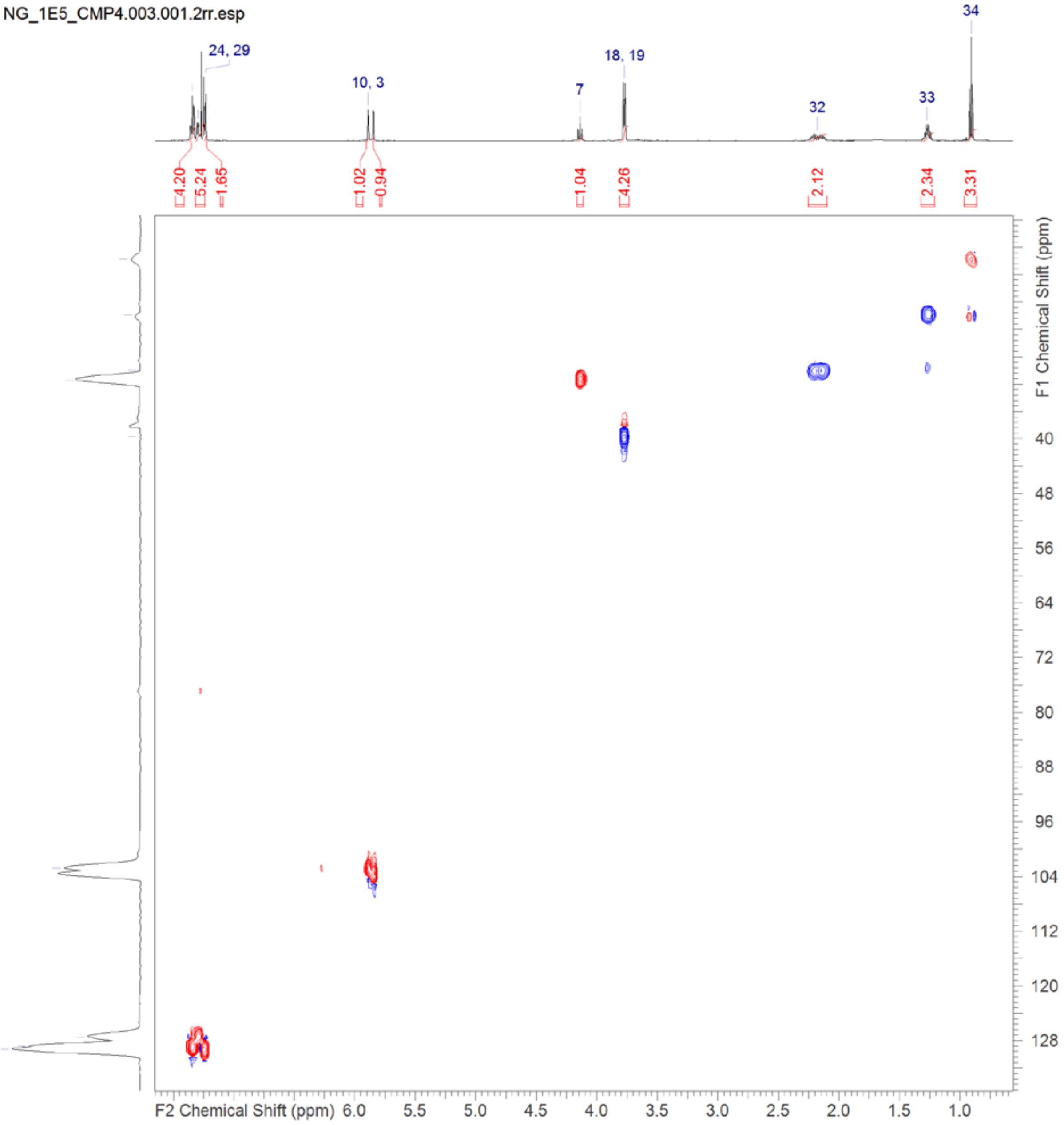

Figure S 66 HSQC-spectrum (500 MHz; 125 MHz, CDCL3) of 1E5_CMP2

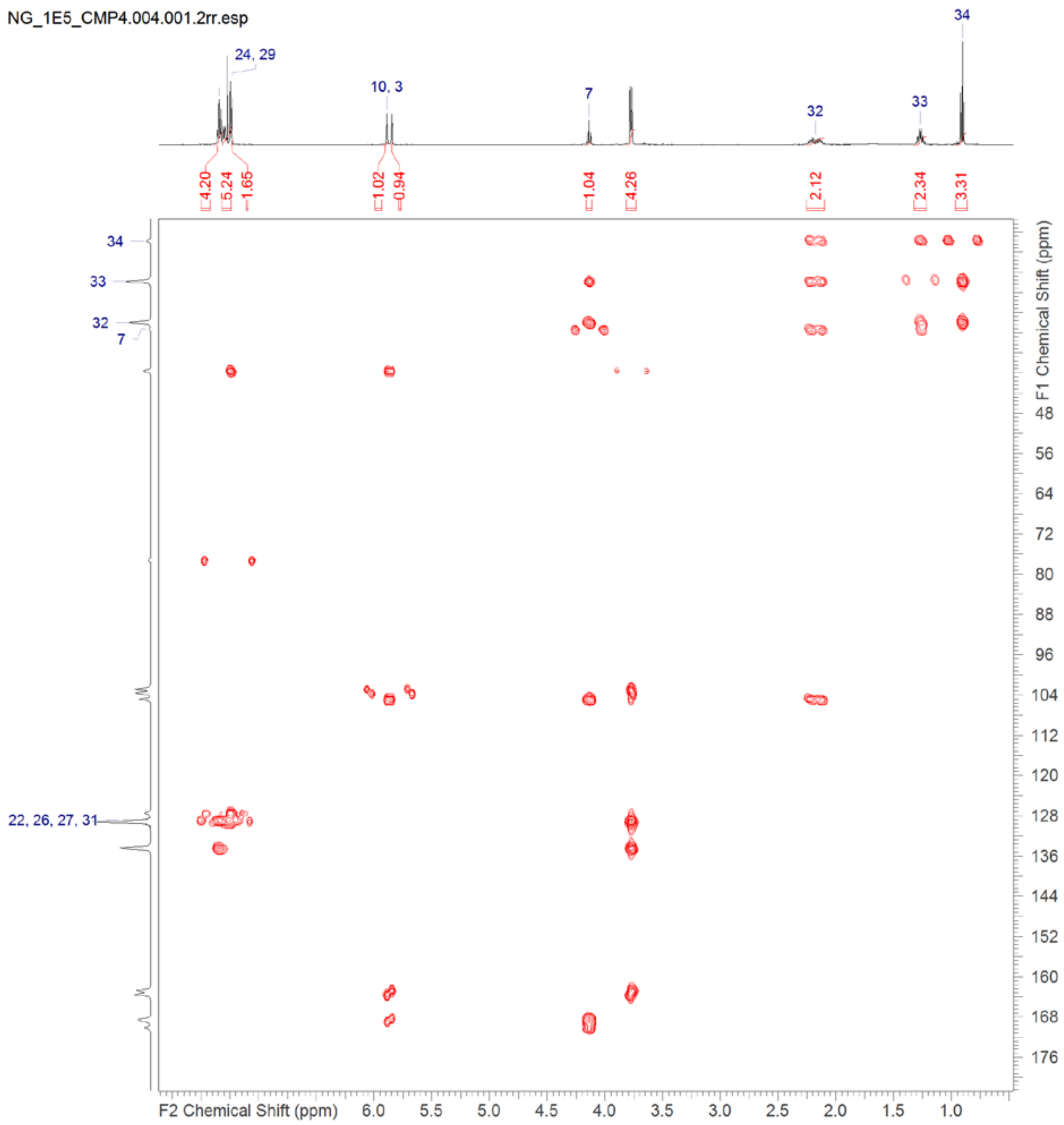

Figure S 67 HMBC-spectrum (500 MHz; 125 MHz, CDCL3) of 1E5_CMP2

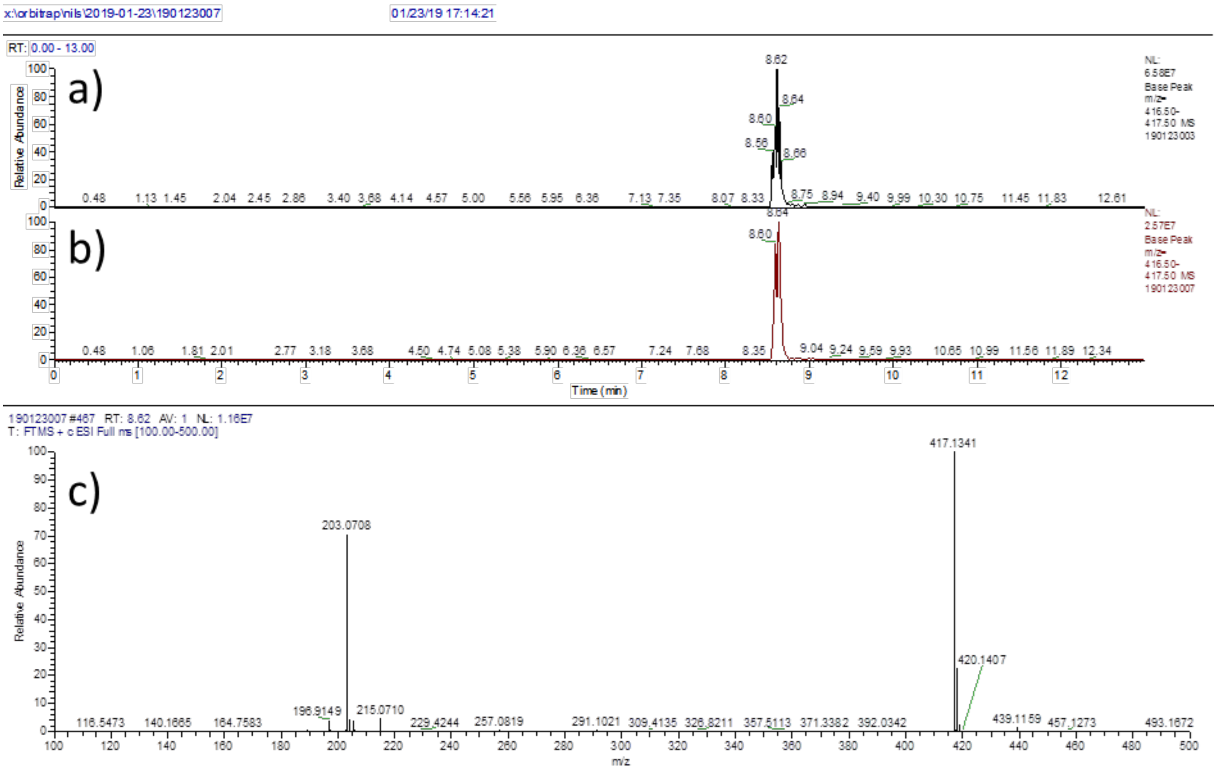

Figure S 68 HPLC-MS Extracted ion chromatogram (Extracted mass 417±0.5) of a) Spontaneously formed 8; b) synthesized 8; c) mass chromatogram of a)

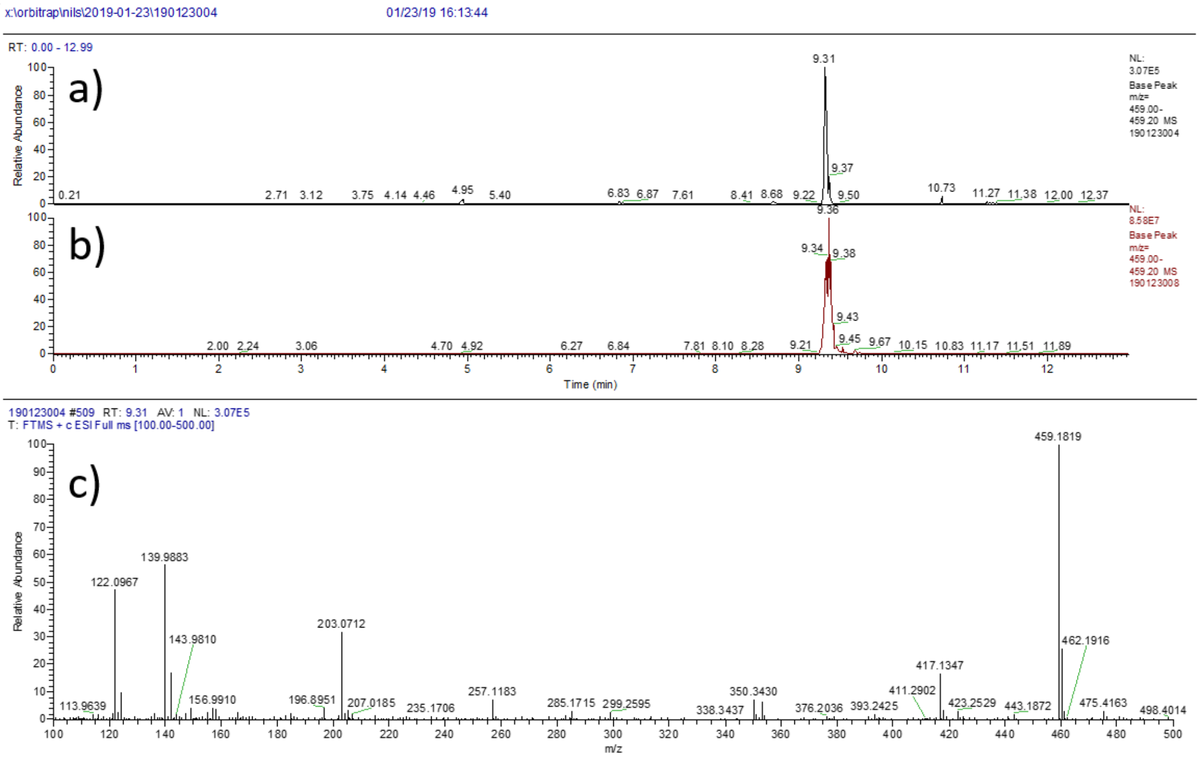

Figure S 69 HPLC-MS Extracted ion chromatogram (Extracted mass 459.1±0.1) of a) Spontaneously formed 9; b) synthesized 9; c) mass chromatogram of a)

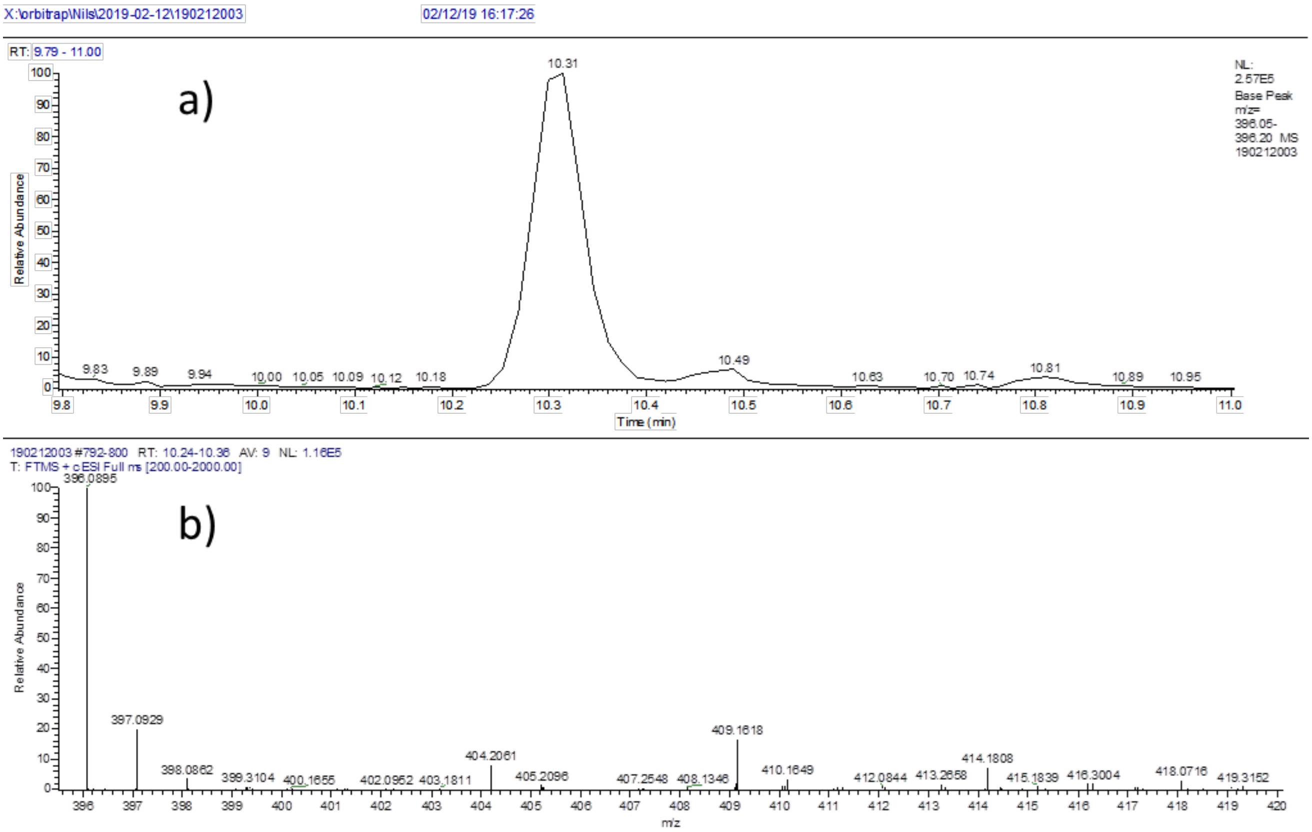

Figure S 70 HPLC Extracted ion chromatogram (Extracted mass 396.05-696.20) of *S. lividans* ΔYA6 1E5 b) mass of SEK43

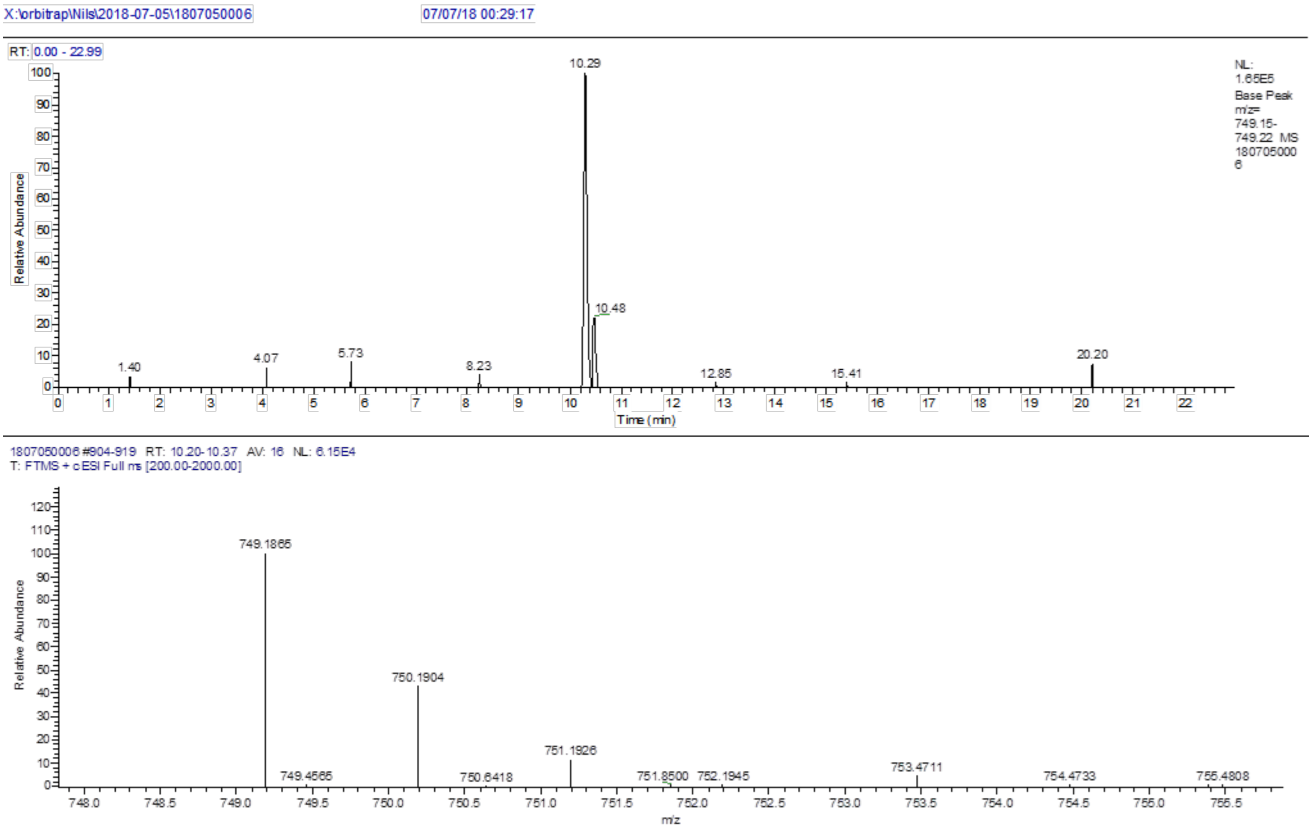

Figure S 71 HPLC Extracted ion chromatogram (Extracted mass 749.15-749.22) of *S. lividans* ΔYA6 1E5 b) mass of SEK87

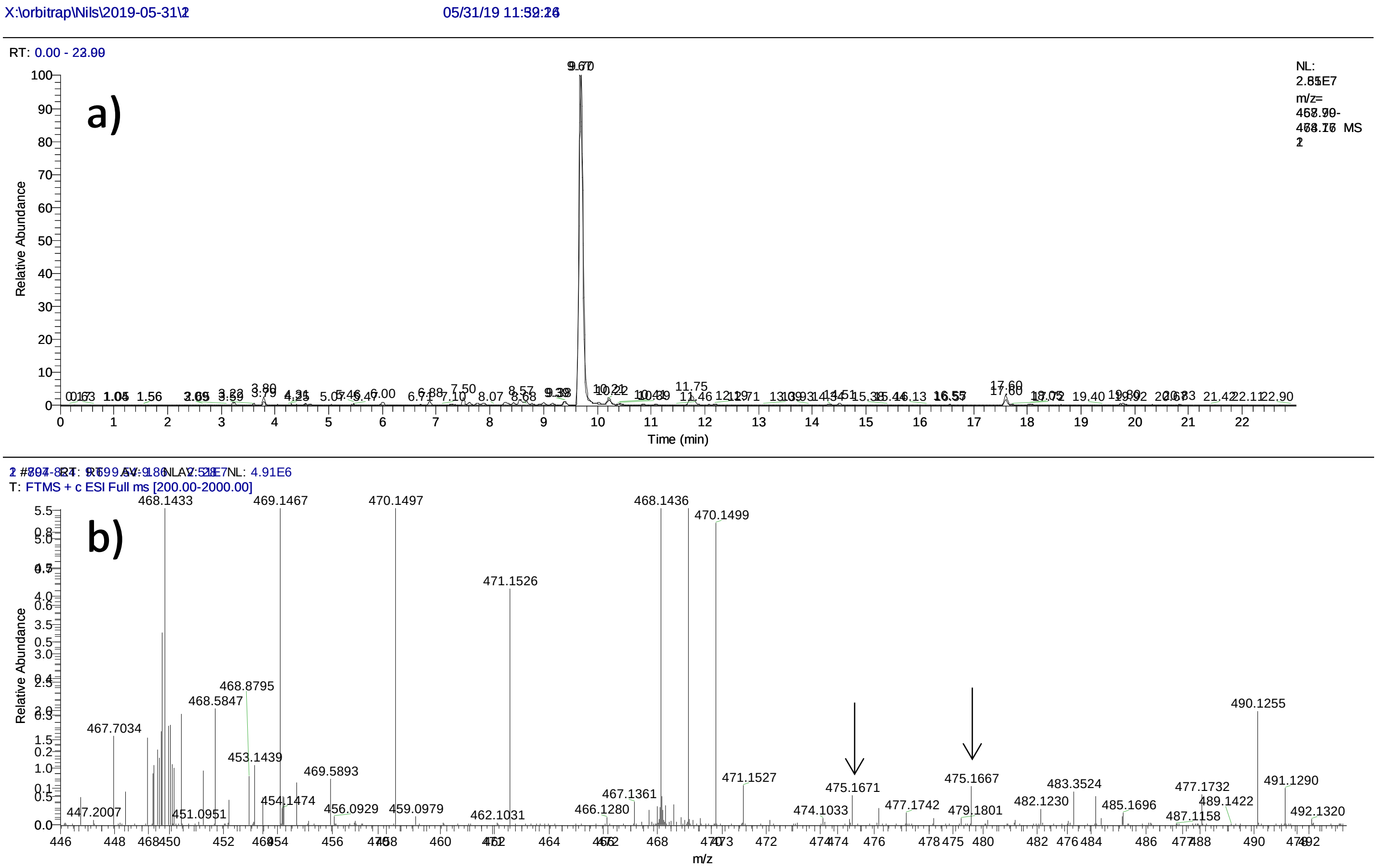

Figure S 72 HPLC-MS Extracted ion chromatogram (Extracted mass 468±0.5) of a) *S. lividans* ΔYA6_1E5 with 13C-9-15N-1-labelled L-tyrosine added to the medium b) MS-Chromatogram of a), incorporation of 13C-9-15N-1-labelled L-tyrosine visible; First repeat of a triplicate

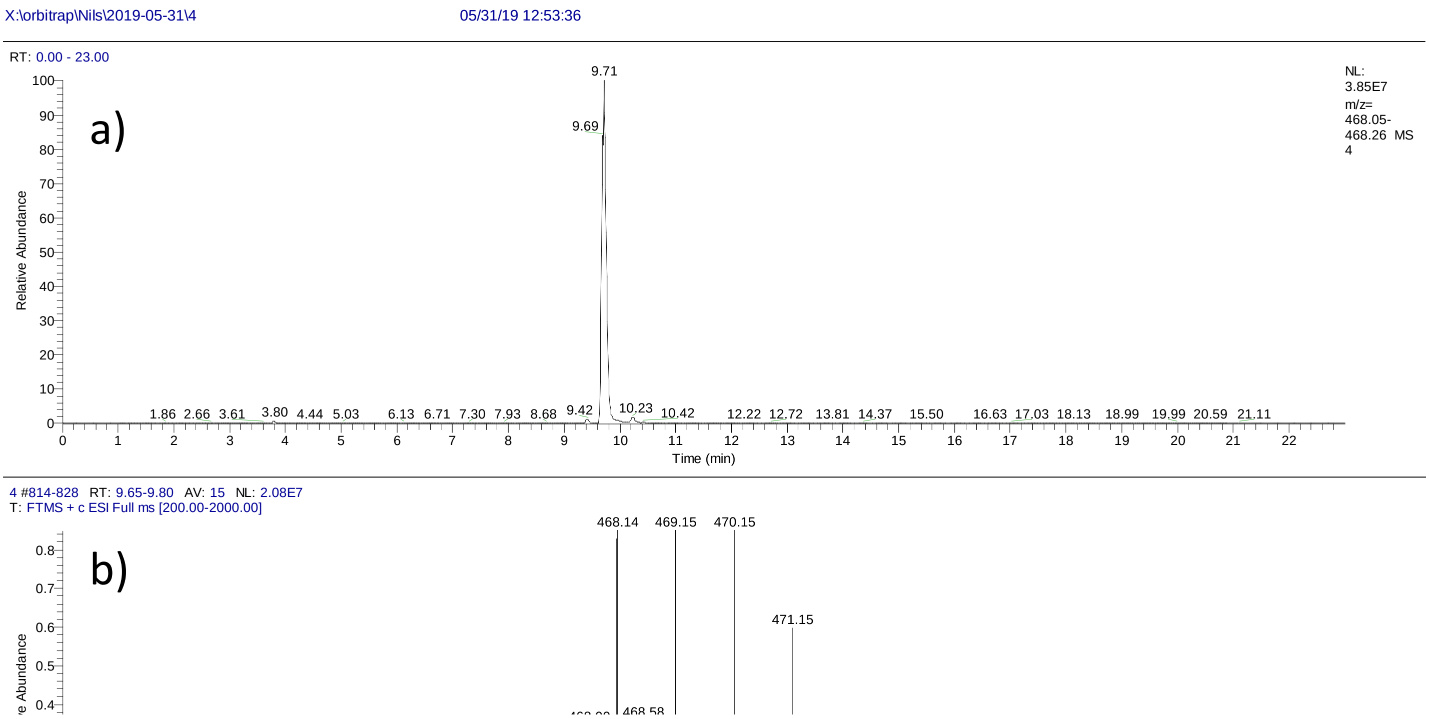

Figure S 73 HPLC-MS Extracted ion chromatogram (Extracted mass 468±0.5) of a) *S. lividans* ΔYA6_1E5 with 13C-9-15N-1-labelled L-tyrosine added to the medium b) MS-Chromatogram of a), incorporation of 13C-9-15N-1-labelled L-tyrosine visible; second repeat of a triplicate

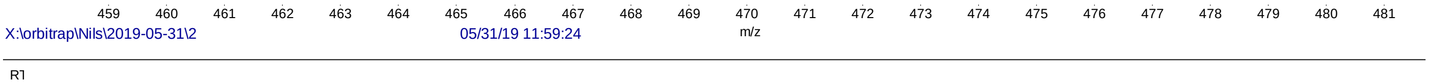

Figure S 75 HPLC-MS Extracted ion chromatogram (Extracted mass 468±0.5) of a) *S. lividans* ΔYA6_1E5 without 13C-9-15N-1-labelled L-tyrosine added to the medium b) MS-Chromatogram of a) no incorporation of 13C-9-15N-1-labelled L-tyrosine visible; first repeat of a triplicate

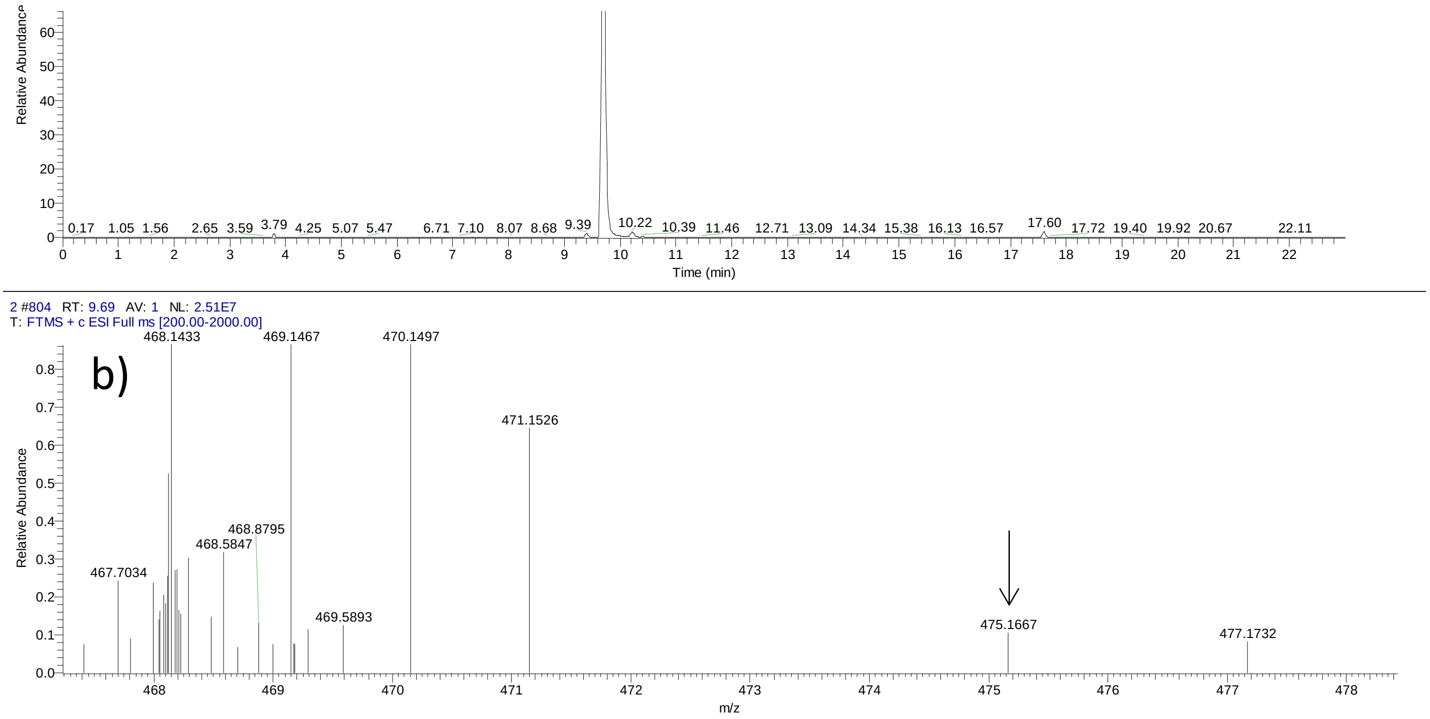

Figure S 74 HPLC-MS Extracted ion chromatogram (Extracted mass 468±0.5) of a) *S. lividans* ΔYA6_1E5 with 13C-9-15N-1-labelled L-tyrosine added to the medium b) MS-Chromatogram of a), incorporation of 13C-9-15N-1-labelled L-tyrosine visible; Third repeat of a triplicate

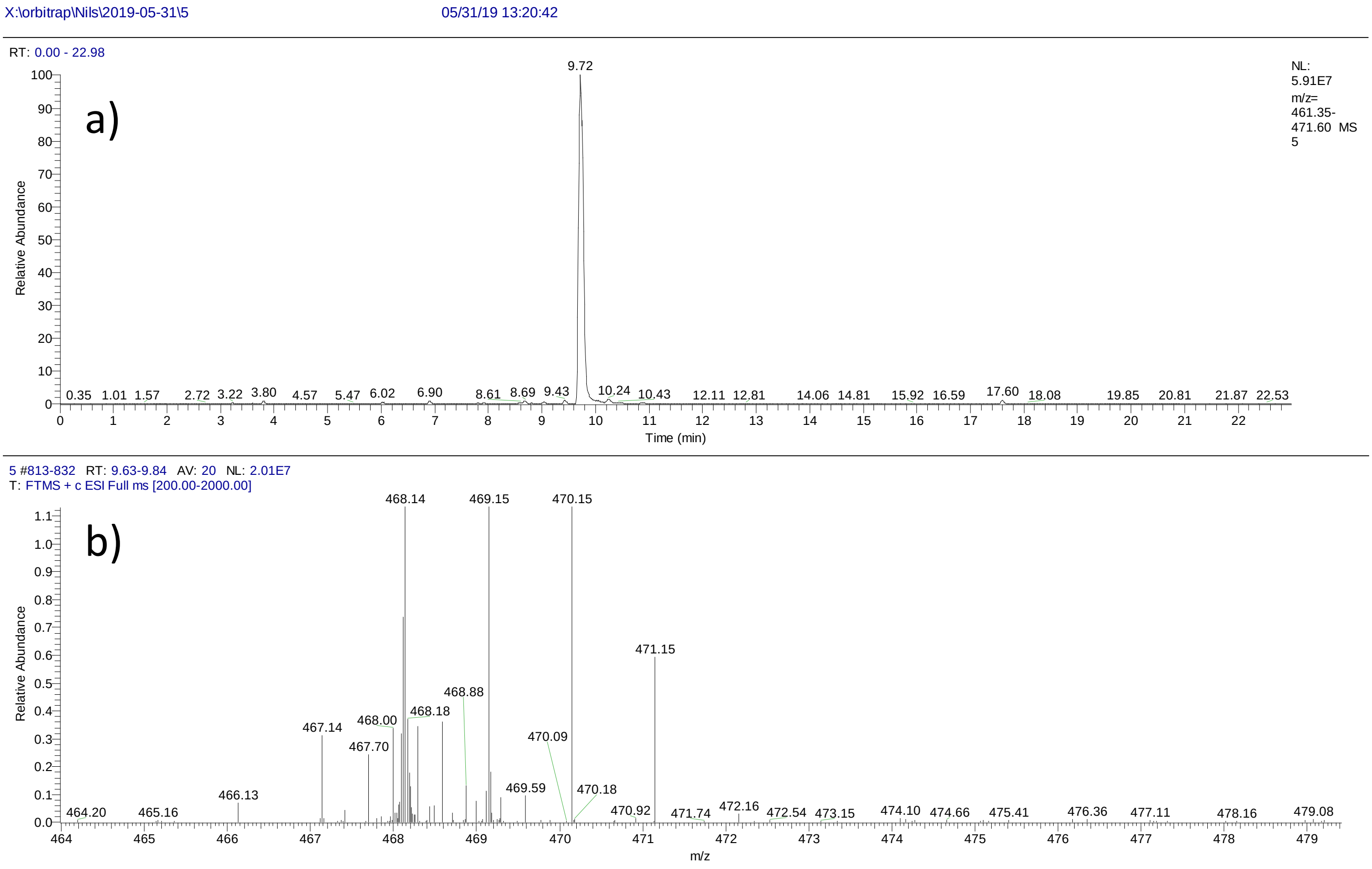

Figure S 76 HPLC-MS Extracted ion chromatogram (Extracted mass 468±0.5) of a) *S. lividans* ΔYA6_1E5 without 13C-9-15N-1-labelled L-tyrosine added to the medium b) MS-Chromatogram of a) no incorporation of 13C-9-15N-1-labelled L-tyrosine visible; second repeat of a triplicate

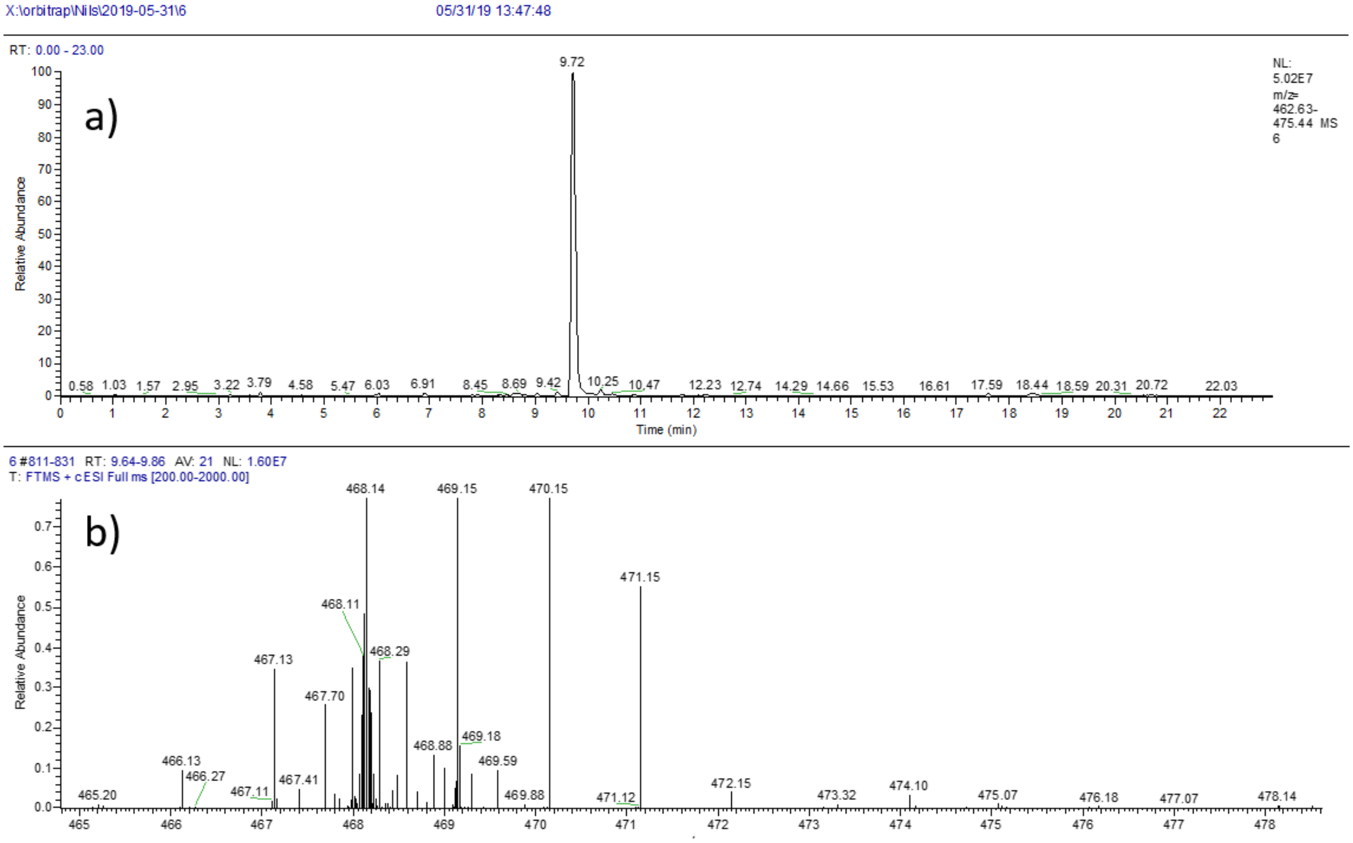

Figure S 77 HPLC-MS Extracted ion chromatogram (Extracted mass 468±0.5) of a) *S. lividans* ΔYA6_1E5 without 13C-9-15N-1-labelled L-tyrosine added to the medium b) MS-Chromatogram of a) no incorporation of 13C-9-15N-1-labelled L-tyrosine visible; third repeat of a triplicate

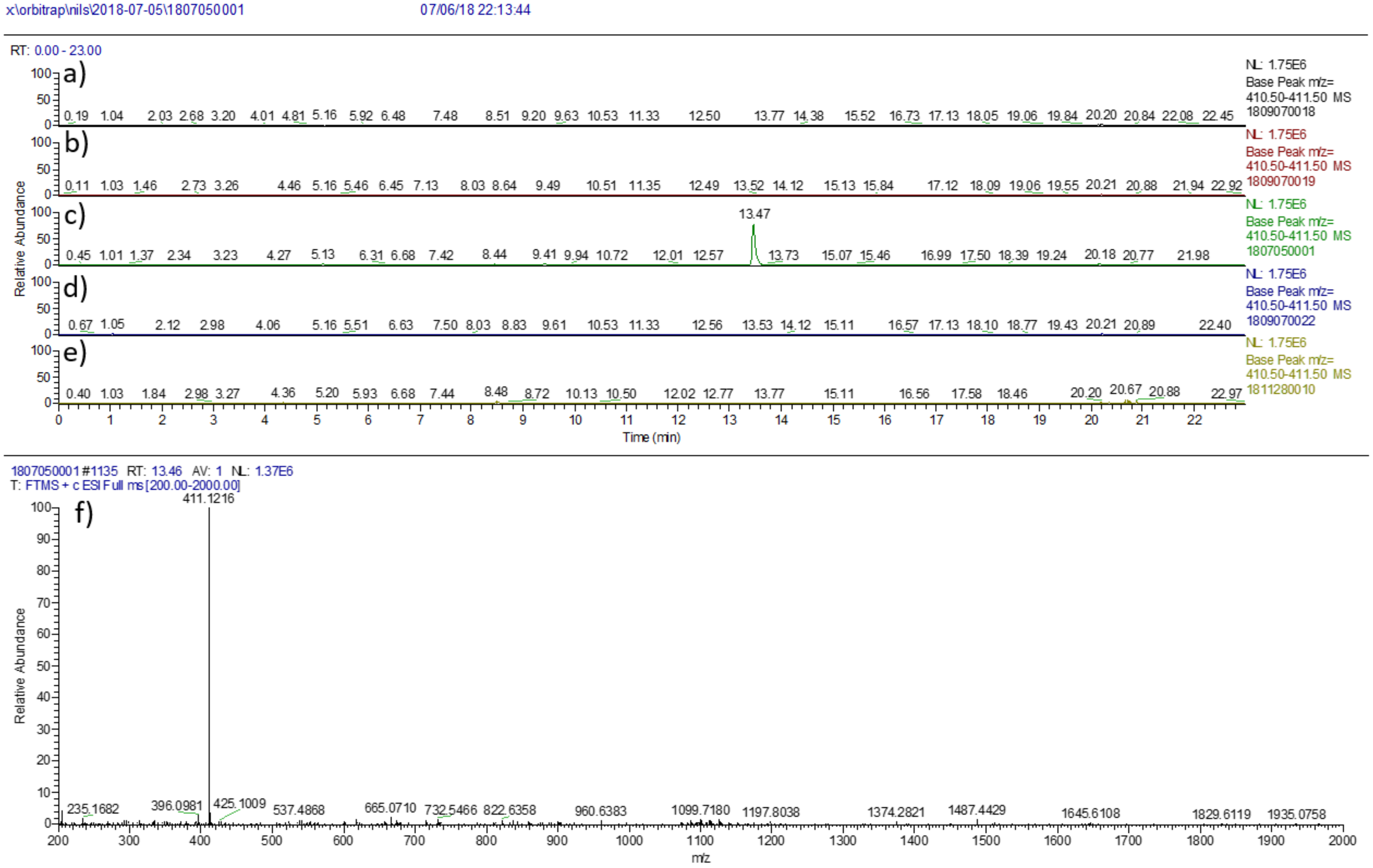

Figure S 78 HPLC-MS Extracted ion chromatogram (Extracted mass 411±0.5; corresponding mass to (3)) of a) *S. lividans* ΔYA6; b) *S. lividans* ΔYA6_1E5; c) *S. lividans* ΔYA6Δ*penA*; d) *S. lividans* ΔYA6Δ*penC; e) S. lividans* ΔYA6Δ*penD;* peak visible in c); f) ESI Full MS chromatogram of the peak visible in c) R_t=13.47

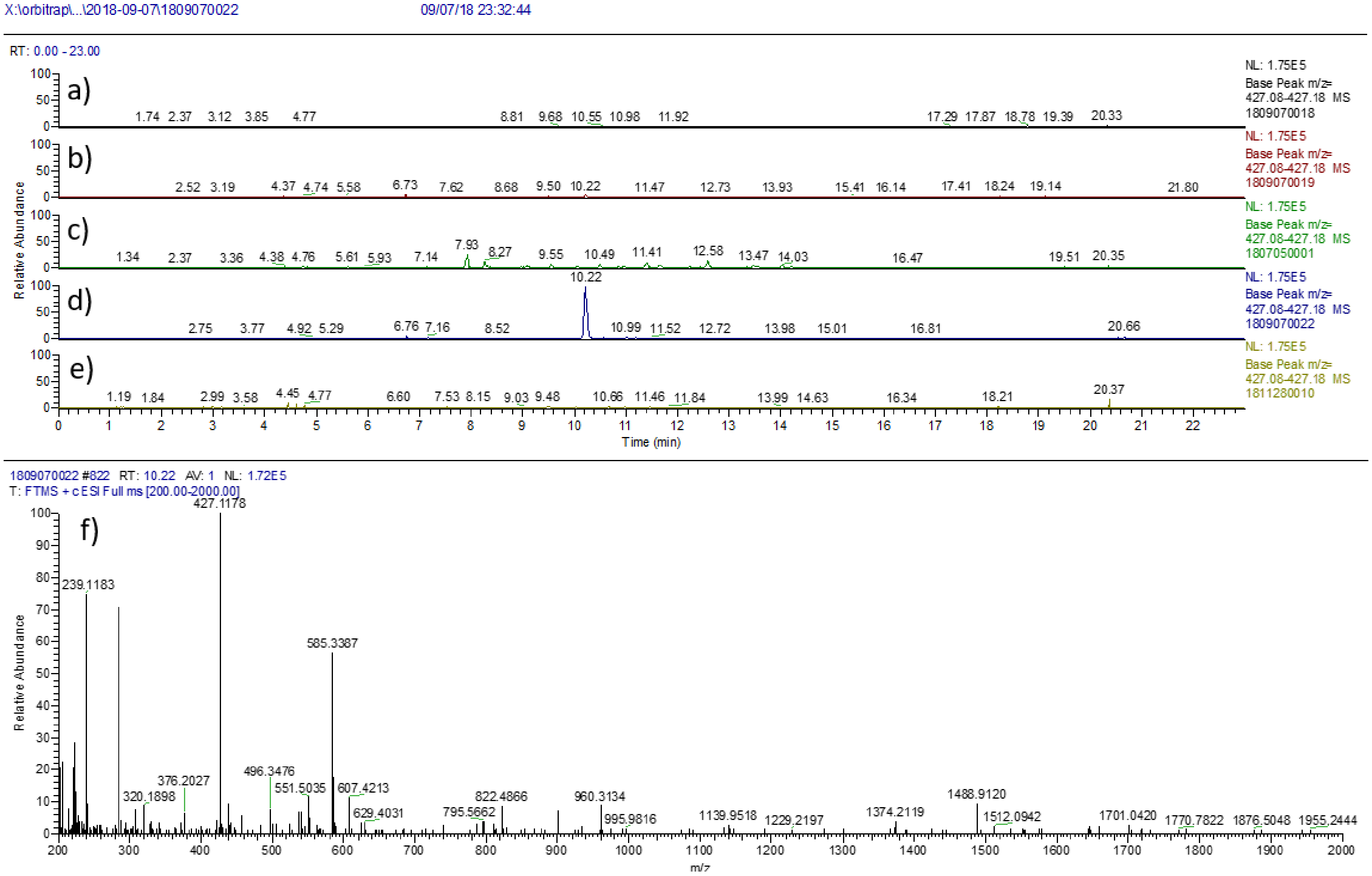

Figure S 79 HPLC-MS Extracted ion chromatogram (Extracted mass 427.09-427.18; corresponding mass to (4)) of a) *S. lividans* ΔYA6; b) *S. lividans* ΔYA6_1E5; c) *S. lividans* ΔYA6Δ*penA*; d) *S. lividans* ΔYA6Δ*penC; e) S. lividans* ΔYA6Δ*penD*; peak visible in d); f) ESI full MS of the peak visible in d) (R_t=10.22)

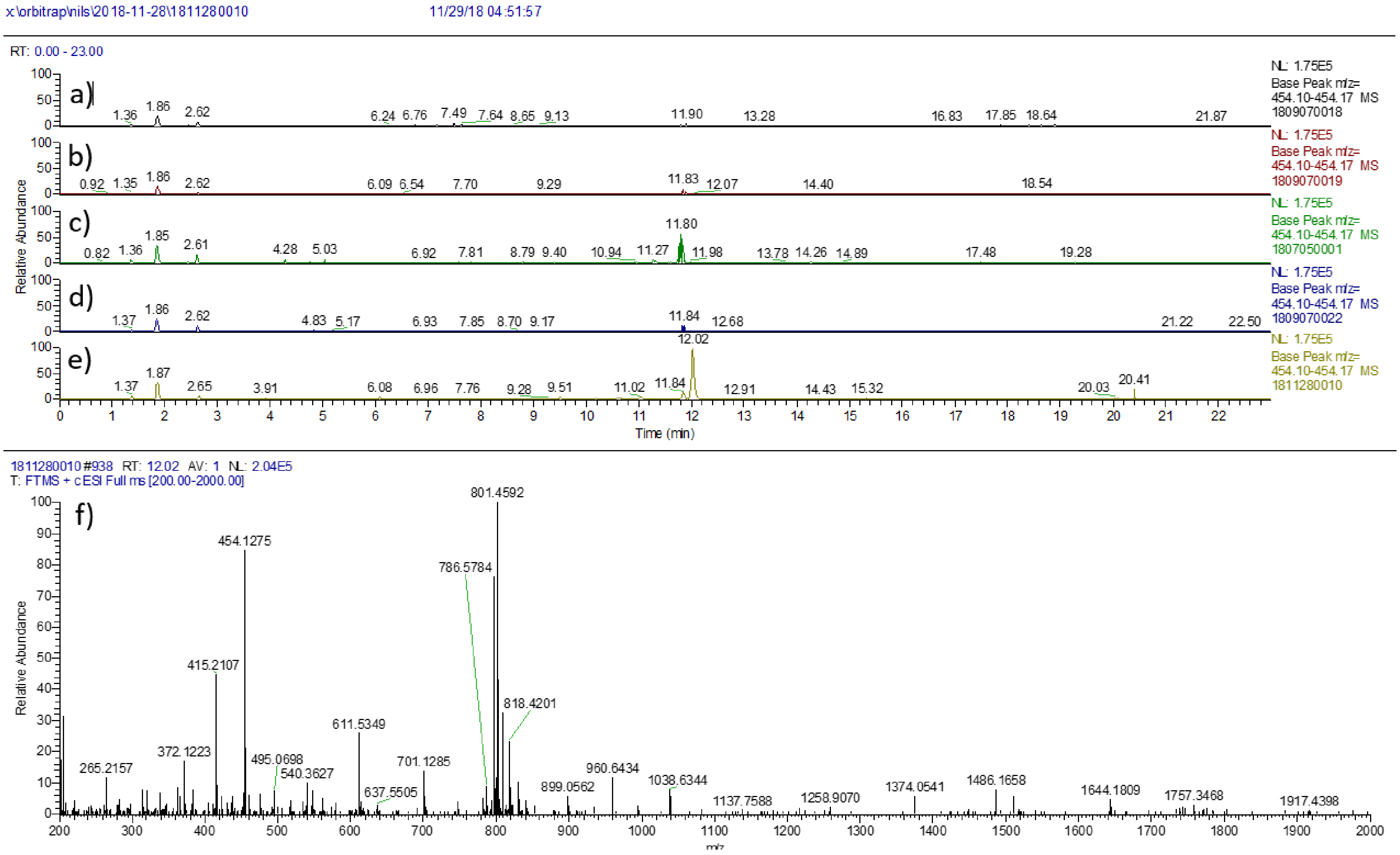

Figure S 80 HPLC-MS Extracted ion chromatogram (Extracted mass 454.10-454.17; corresponding mass to (5)) of a) *S. lividans* ΔYA6; b) *S. lividans* ΔYA6_1E5; c) *S. lividans* ΔYA6Δ*penA*; d) *S. lividans* ΔYA6Δ*penC; e) S. lividans* ΔYA6Δ*penD*; peak visible in e); f) f) ESI full MS of the peak visible in e) (R_t=12.02)

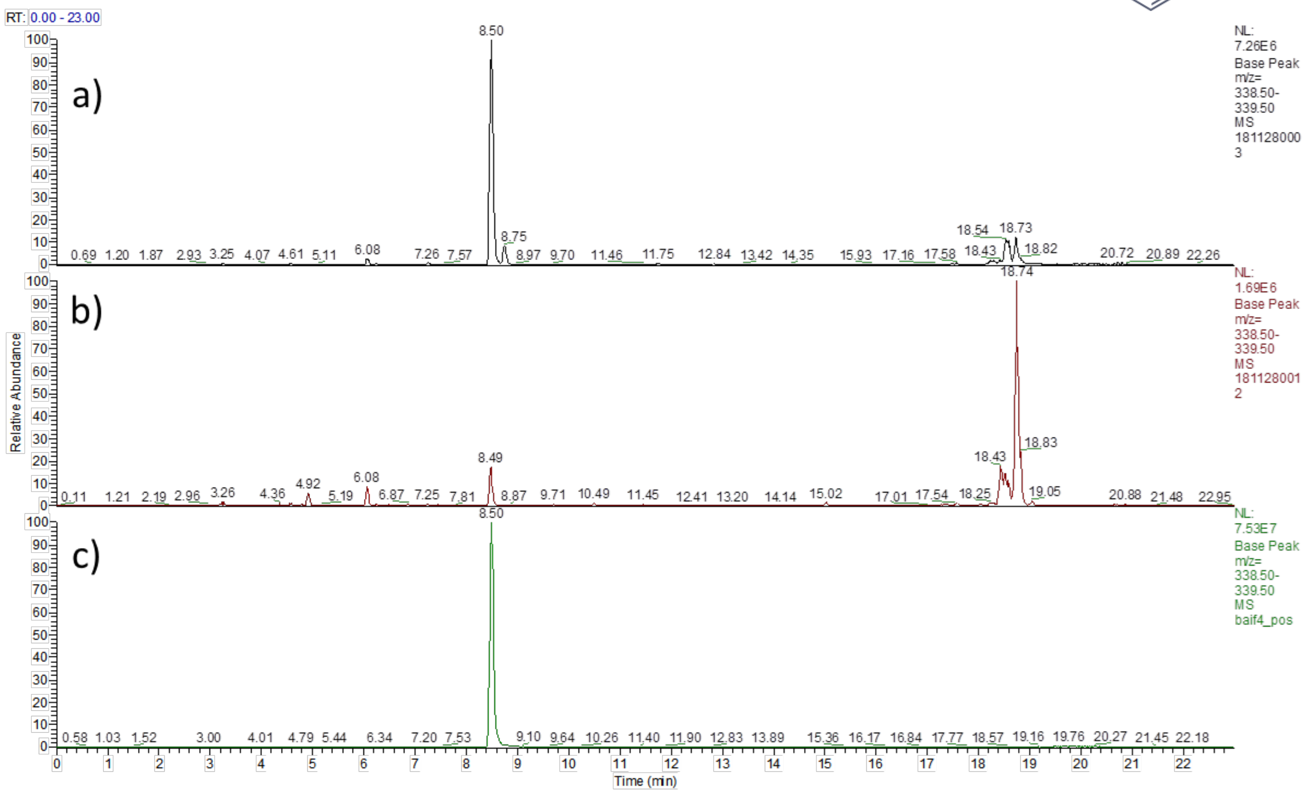

Figure S 81 HPLC-MS Extracted ion chromatogram (Extracted mass 339±0.5, Rabelomycin) of a) *S. lividans* ΔYA6 1E5, b) *S. lividans* ΔYA6 1E5ΔpenE c) pure rabelomycin as external standard

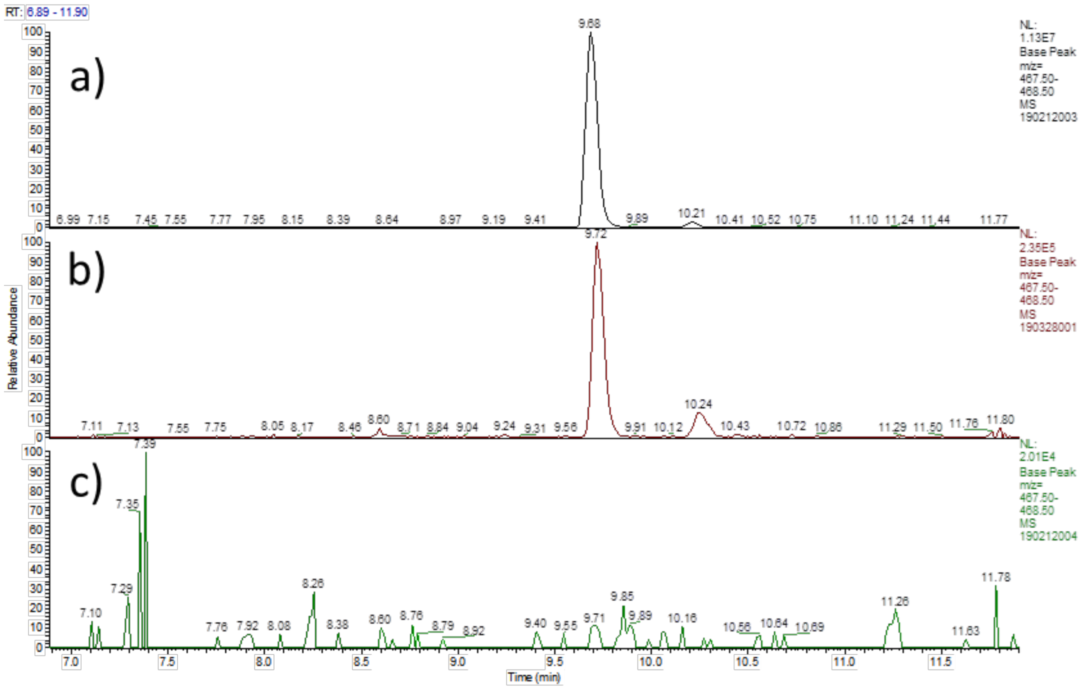

Figure S 82 HPLC-MS Extracted ion chromatogram (Extracted mass 468±0.5, (1)) of a) *S. lividans* ΔYA6 1E5, b) *S. lividans* ΔYA6 1E5ΔpenR1 and c) *S. lividans* ΔYA6 1E5ΔpenR2

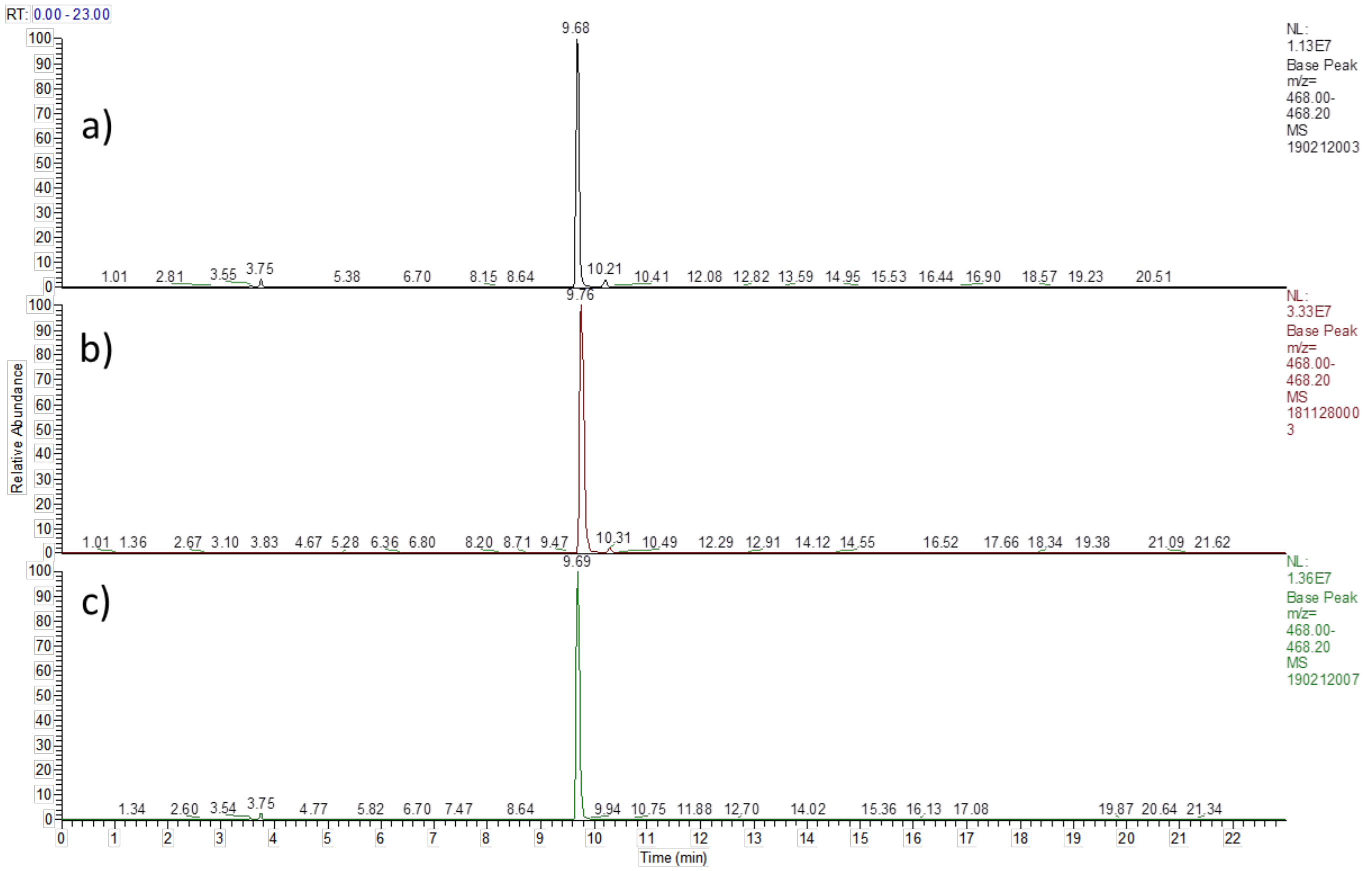

Figure S 83 HPLC-MS Extracted ion chromatogram (Extracted mass 468±0.5 (1)) of a) *S. lividans* ΔYA6 1E5, b) *S. lividans* ΔYA6 A3_penR1 1E5 and c) *S. lividans* ΔYA6 A3_penR2 1E5

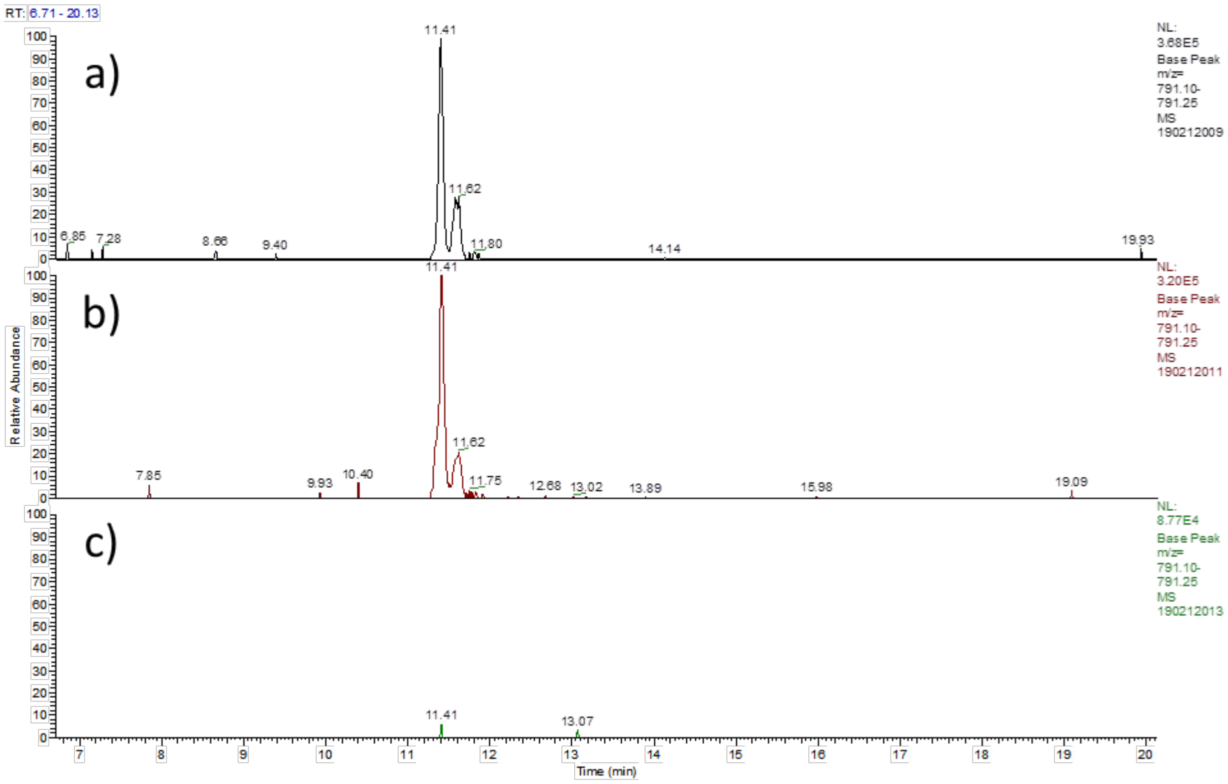

Figure S 84 HPLC-MS Extracted ion chromatogram (Extracted mass 791.10-791.25 (2)) of a) *S. lividans* ΔYA6 1E5, b) *S. lividans* ΔYA6 A3_penR1 1E5 and c) *S. lividans* ΔYA6 A3_penR2 1E5

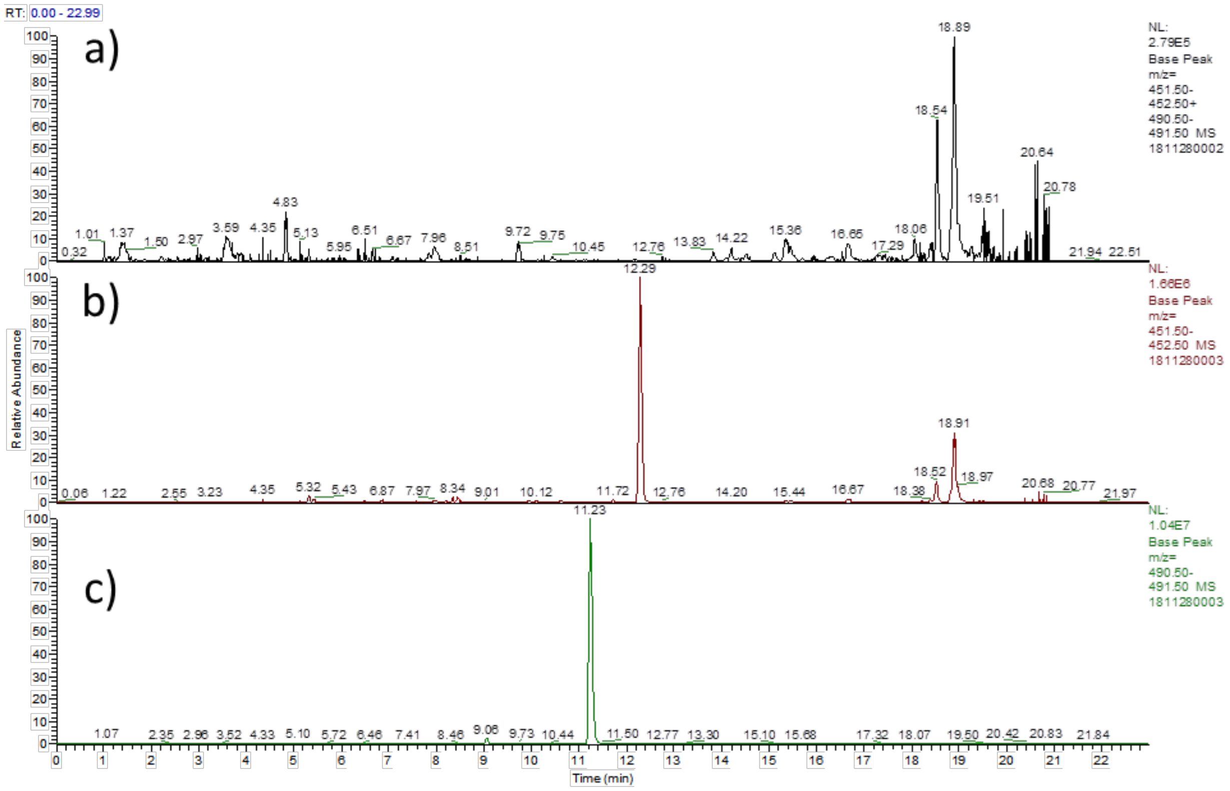

Figure S 85 HPLC-MS Extracted ion chromatogram a) *S. lividans* ΔYA6 A3_penR1 (Extracted mass 491±0.5 (6) and 452±0.5 (7)), b) *S. lividans* ΔYA6 A3_penR1 1E5 (Extracted mass 452±0.5 (7)) c) *S. lividans* ΔYA6 A3_penR1 1E5 (Extracted mass 491±0.5 (6))

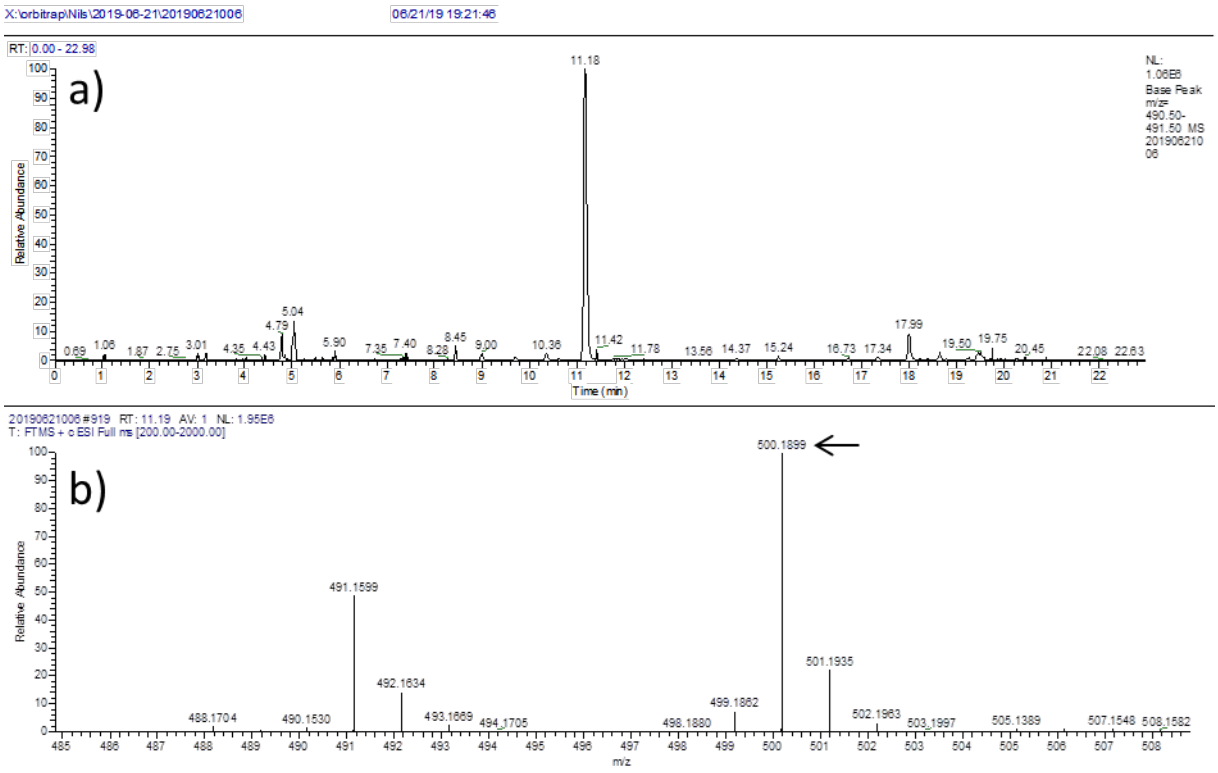

Figure S 86 HPLC-MS Extracted ion chromatogram (Extracted mass 491±0.5) of a) *S. lividans* ΔYA6_A3_penR1 1E5 with L-tryptophan 13C-11 added to the medium b) MS-Chromatogram of a), incorporation of L-tryptophan 13C-11 visible

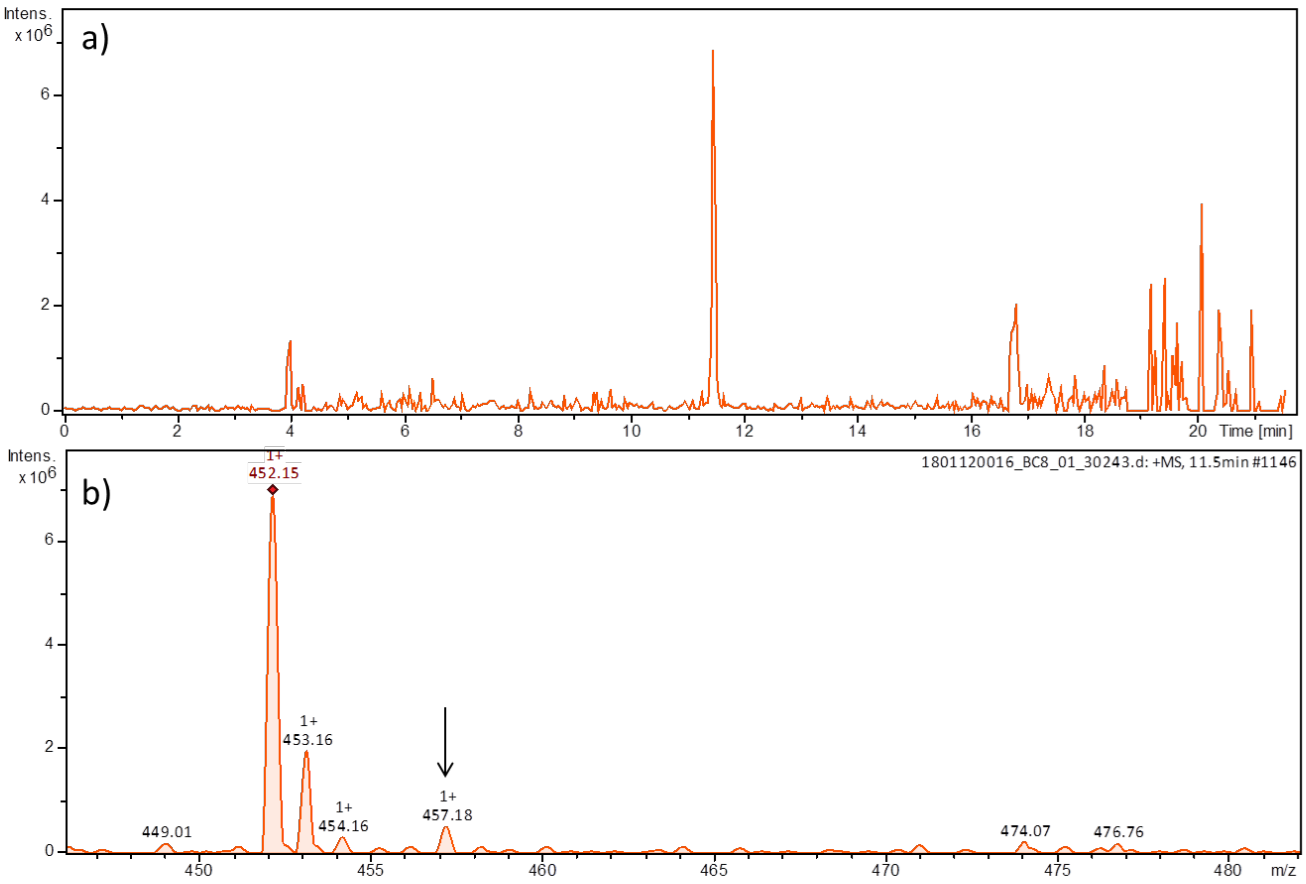

Figure S 87 HPLC-MS Extracted ion chromatogram (Extracted mass 452±0.5) of a) *S. lividans* ΔYA6_A3_penR1 1E5 with 2D-5-labelled L-Phenylalanine added to the medium b) MS-Chromatogram of a), incorporation of 2D-5-labelled L-Phenylalanine visible

1. (1) Chater, K. F.; Wilde, L. C. *J Gen Microbiol* 1980, *116*, 323-334.
2. (2) Labeda, D. P.; Lechevalier, M. P. *International Journal of Systematic and Evolutionary Microbiology* 1989, *39*, 420-423.
3. (3) Wang, J.; Sarov, M.; Rientjes, J.; Fu, J.; Hollak, H.; Kranz, H.; Xie, W.; Stewart, A. F.; Zhang, Y. *Mol Biotechnol* 2006, *32*, 43-53.
4. (4) Kieser, T.; Bibb, M. J.; Buttner, M. J.; Chater, K. F.; Hopwood, D. A. 2010.
5. (5) Strochlic, T. I.; Viaud, J.; Rennefahrt, U. E.; Anastassiadis, T.; Peterson, J. R. *Mol Cell* 2010, *40*, 493-500.
6. (6) Herrmann, S.; Siegl, T.; Luzhetska, M.; Petzke, L.; Jilg, C.; Welle, E.; Erb, A.; Leadlay, P. F.; Bechthold, A.; Luzhetskyy, A. *Appl Environ Microbiol* 2012, *78*, 1804-1812.
7. (7) Myronovskyi, M.; Rosenkranzer, B.; Luzhetskyy, A. *Appl Microbiol Biotechnol* 2014, *98*, 4557-4570.
8.

Publisher's Note: MDPI stays neutral with regard to jurisdictional claims in published maps and institutional affiliations.

© 2020 by the authors. Submitted for possible open access publication under the terms and conditions of the Creative Commons Attribution (CC BY) license (http://creativecommons.org/licenses/by/4.0/).
